# Topological abnormalities of the morphometric similarity network of the cerebral cortex in schizophrenia

**DOI:** 10.1038/s41537-024-00477-x

**Published:** 2024-06-17

**Authors:** Sung Woo Joo, Young Tak Jo, Woohyeok Choi, Sun Min Kim, So Young Yoo, Soohyun Joe, Jungsun Lee

**Affiliations:** 1 Department of Psychiatry, Asan Medical Center, University of Ulsan College of Medicine, Seoul, Republic of Korea; 2 Department of Psychiatry, Kangdong Sacred Heart Hospital, Hallym University College of Medicine, Seoul, Republic of Korea; 3 Department of Psychiatry, SMG-SNU Boramae Medical Center, Seoul National University College of Medicine, Seoul, Republic of Korea; 4 Brain Laboratory, Department of Psychiatry, University of California San Diego, School of Medicine, San Diego, California, USA

**Keywords:** Schizophrenia, Schizophrenia, Biomarkers

## Abstract

A morphometric similarity (MS) network can be constructed using multiple magnetic resonance imaging parameters of each cortical region. An MS network can be used to assess the similarity between cortical regions. Although MS networks can detect microstructural alterations and capture connections between histologically similar cortical areas, the influence of schizophrenia on the topological characteristics of MS networks remains unclear. We obtained T1- and diffusion-weighted images of 239 healthy controls and 190 individuals with schizophrenia to construct the MS network. Group comparisons of the mean MS of the cortical regions and subnetworks were performed. The strengths of the connections between the cortical regions and the global and nodal network indices were compared between the groups. Clinical associations with the network indices were tested using Spearman’s rho. Compared with healthy controls, individuals with schizophrenia had significant group differences in the mean MS of several cortical regions and subnetworks. Individuals with schizophrenia had both superior and inferior strengths of connections between cortical regions compared with those of healthy controls. We observed regional abnormalities of the MS network in individuals with schizophrenia regarding lower centrality values of the pars opercularis, superior frontal, and superior temporal areas. Specific nodal network measures of the right pars opercularis and left superior temporal areas were associated with illness duration in individuals with schizophrenia. We identified regional abnormalities of the MS network in schizophrenia with the left superior temporal area possibly being a key region in topological organization and cortical connections.

## Introduction

Despite the enormous socioeconomic burden of schizophrenia^1^, the neurobiological background of the etiology and pathophysiology of schizophrenia is still elusive. Structural abnormalities of brain regions in individuals with schizophrenia have been consistently reported; however, the individual results vary^2^. Dysconnectivity among brain regions has been hypothesized to be the primary pathophysiology for schizophrenia, suggesting that abnormal connections among cortical and subcortical regions contribute to clinical symptoms of schizophrenia, together with structural and functional changes in individual brain regions. Accordingly, abnormalities in intra- and inter-networks in individuals with schizophrenia have drawn increasing attention^3^. A meta-analysis of resting-state functional connectivity in individuals with schizophrenia reported an imbalance in connections among large-scale brain networks and revealed the core role of the ventral attention network in a disconnected brain network model^4^. Consequently, the abnormalities in individual brain regions and their connections should be considered in tandem to clarify the neural mechanisms involved in schizophrenia.

The brain network can be conceptualized as a graph in which nodes are brain regions, and edges are their connections. Graph-based network analysis has the advantage of resulting in several network properties reflective of the segregation and integration of individual brain regions^5^. The topological characteristics of brain networks in individuals with schizophrenia include less efficient wiring networks and a low prevalence of local clustering and hierarchical organization^6^. The results indicate disrupted network organization with widespread and local network disturbances in individuals with schizophrenia. However, the findings are inconclusive. The issues of topological changes in the brain networks in schizophrenia may be related to which morphometric features of the brain are utilized to construct the brain networks and which network indices are adopted to investigate changes in the topological characteristics of the brain networks. Therefore, further studies with various brain network models are needed to deepen the understanding of the topological characteristics of brain networks in individuals with schizophrenia.

Several methods for constructing cortical brain networks have been proposed, including structural covariance and tractography-based structural networks. Despite the advantages of previous methods, structural covariance networks, which are based on the principle that connected brain regions covary in their morphological properties, usually rely on single morphometric features such as cortical thickness and gray matter volume^7^, and tractography-based structural networks exhibit limited ability to capture a few network measures, possibly due to false positives in tractography^8^. Seidlitz et al.^9^. proposed an alternative cortical brain network based on multiple magnetic resonance imaging (MRI) parameters of each cortical region and similarity between cortical regions, namely, the morphometric similarity (MS) network. The MS network is compatible with structural covariance and tractography-based structural networks regarding recapitulating the topological organization of the cerebral cortex, including several modules and high-degree hubs. The MS network captures more connections between cortical regions with the same cytoarchitectonic class, which supports the biological validity of the MS network and aligns with growing evidence that cytoarchitectonic similarity between cortical regions is associated with a greater probability of axonal connectivity^10,11^.

Additionally, in healthy participants, individual variations in regional mean MSs of the cortical regions account for approximately 40% of inter-individual variability in general intelligence. Given the limitations of conventional MRI indices as surrogate markers and the advantages of utilizing several MRI parameters to precisely estimate the structural integrity of cortical regions^12–14^, MS networks based on multiple MRI morphometric features may be more beneficial than conventional methods. MS networks could aid in revealing microstructural changes in cortical regions and topological characteristics of brain networks in individuals with schizophrenia.

Regarding abnormalities in MS networks in individuals with psychosis, Morgan et al.^15^. estimated regional MS as the mean MS of cortical regions and used these values to represent hub function. They reported globally reduced MS in individuals with psychosis and significant case‒control differences in regional MS in several cortical regions. In individuals with psychosis, the regional MS decreased in the frontal and temporal regions and increased in the parietal areas. The cortical pattern of case‒control differences in regional MS was spatially correlated with the expression of genes associated with schizophrenia and antipsychotic treatments, which suggested the biological relevance of abnormalities in regional MS in individuals with psychosis. However, it is uncertain whether individuals with psychosis exhibit alterations in topological characteristics of the MS network except for regional MS, and abnormalities in topological characteristics in individuals with psychosis have clinical implications.

In this study, we comprehensively investigated the abnormalities of the MS network at the node and network level in individuals with schizophrenia compared with healthy controls (HCs), the mean MS of cortical regions and subnetworks, and the strength of the connections between the cortical regions in the MS network and the topological abnormalities of the MS network. We hypothesized that individuals with schizophrenia have abnormalities in topological characteristics at different levels of the MS network and significant differences in the strengths of connections between cortical regions compared with HCs.

## Results

### Abnormal morphometric similarity at the node level

**Fig. 1:**
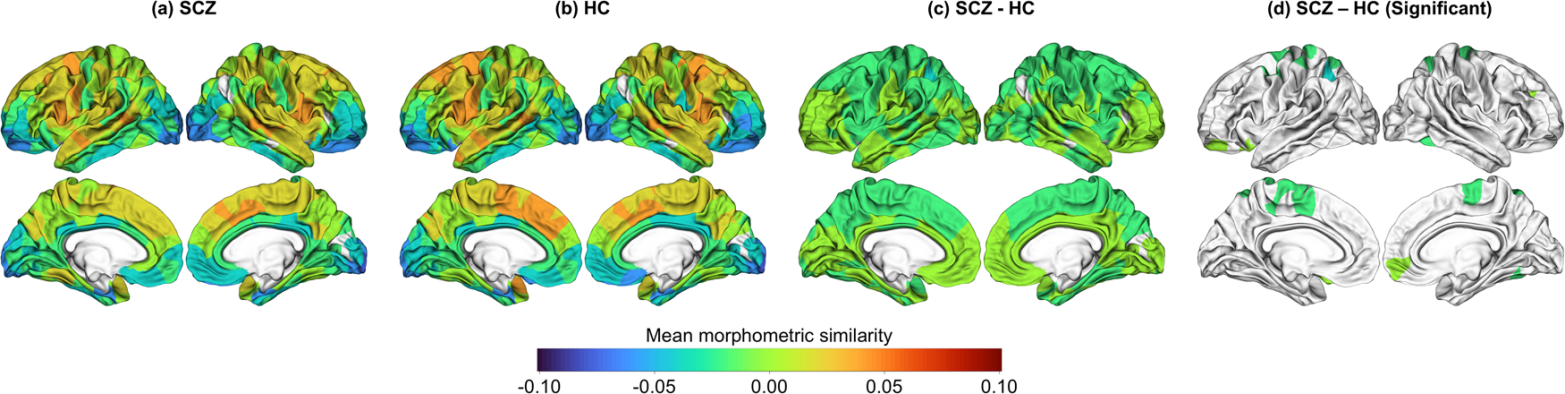
Group comparisons of the mean morphometric similarity of cortical regions. Red and blue colors reflect higher and lower mean morphometric similarity of cortical regions, respectively. The mean morphometric similarity of cortical regions in the patient and control groups is shown in (**a**, **b**), respectively. Group differences in the mean morphometric similarity are presented in (**c**), and cortical regions with statistical significance based on FDR *q* < 0.05 are shown in (**d**). HC healthy control, SCZ schizophrenia.

Among the 308 cortical regions, 18 cortical regions exhibited significant group differences in mean MS between individuals with schizophrenia and HCs (Fig. 1 and Supplementary Table 2). Fourteen cortical regions had a significantly lower mean MS in individuals with schizophrenia than in HCs. Of the 14 subnetworks, five had a lower mean MS in the patient group than in the control group, namely, the somatosensory, dorsal attention, and limbic subnetworks in the Yeo atlas and the primary motor cortex and association cortex 1 in the Von Economo atlas (Supplementary Figure 1 and Supplementary Table 3).

### Abnormal morphometric similarity at the network level

**Fig. 2:**
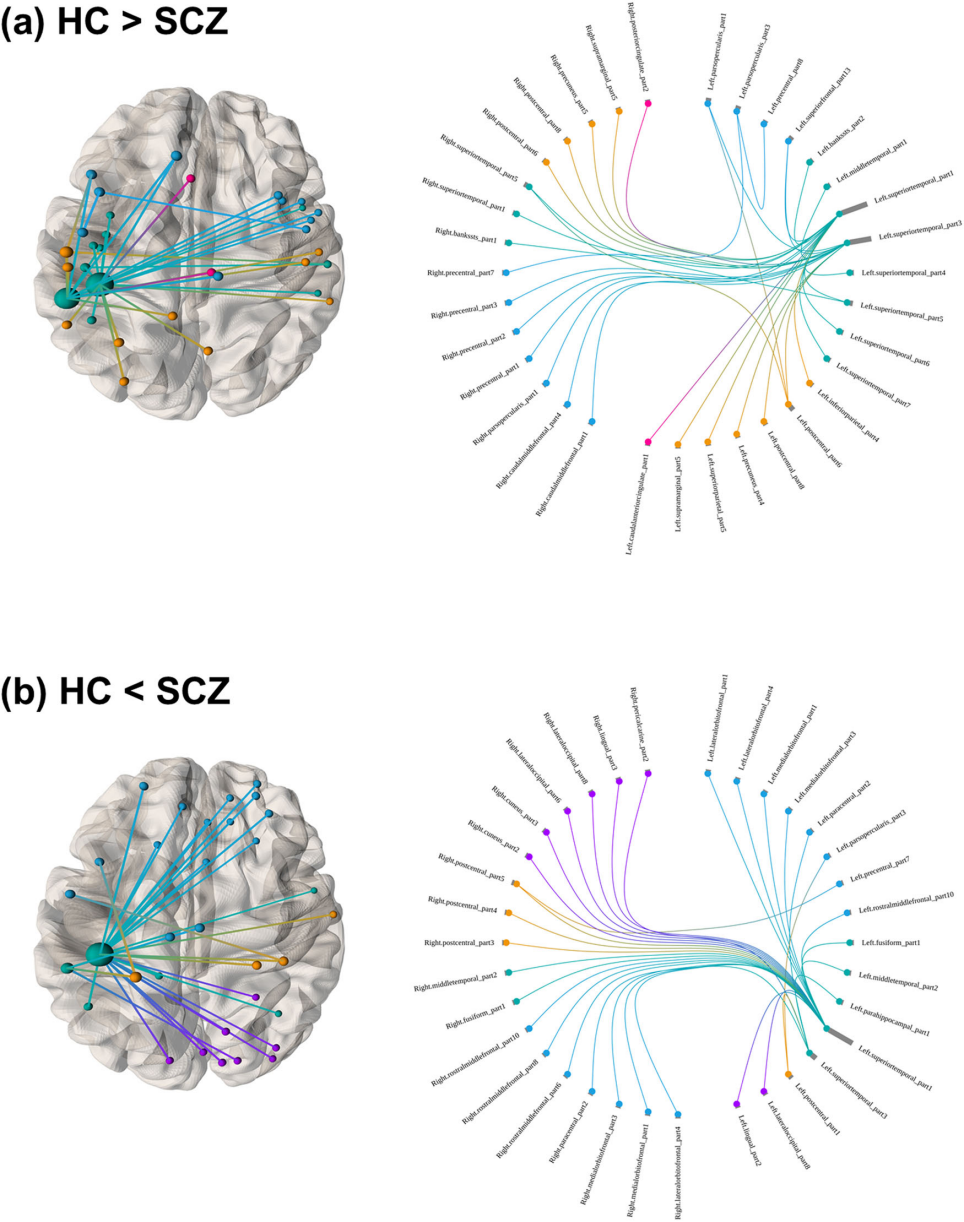
Group comparisons of the connections of the morphometric similarity network between individuals with schizophrenia and healthy controls. The figures exhibit the significant connections after the adjustment with the network-based statistic; (**a**) healthy controls > individuals with schizophrenia and (**b**) healthy controls < individuals with schizophrenia. The connectogram circumference demonstrates that the left hemisphere on the right side and brain regions are grouped (frontal [blue], temporal [green], parietal [orange], occipital [purple], and cingulate [pink]). The size of the nodes in the brain maps represents the number of significant connections. HC healthy control; SCZ schizophrenia.

Figure 2 displays the connections among the cortical regions that were significantly stronger or weaker in individuals with schizophrenia than in HCs. Among all the cortical region connections (*n* = 47,228), 32 and 34 connections were stronger and weaker, respectively, in the patient group than in the control group. The left superior temporal area was involved in most of the connections that were either significantly stronger or weaker in the patient group than in the control group.

**Fig. 3:**
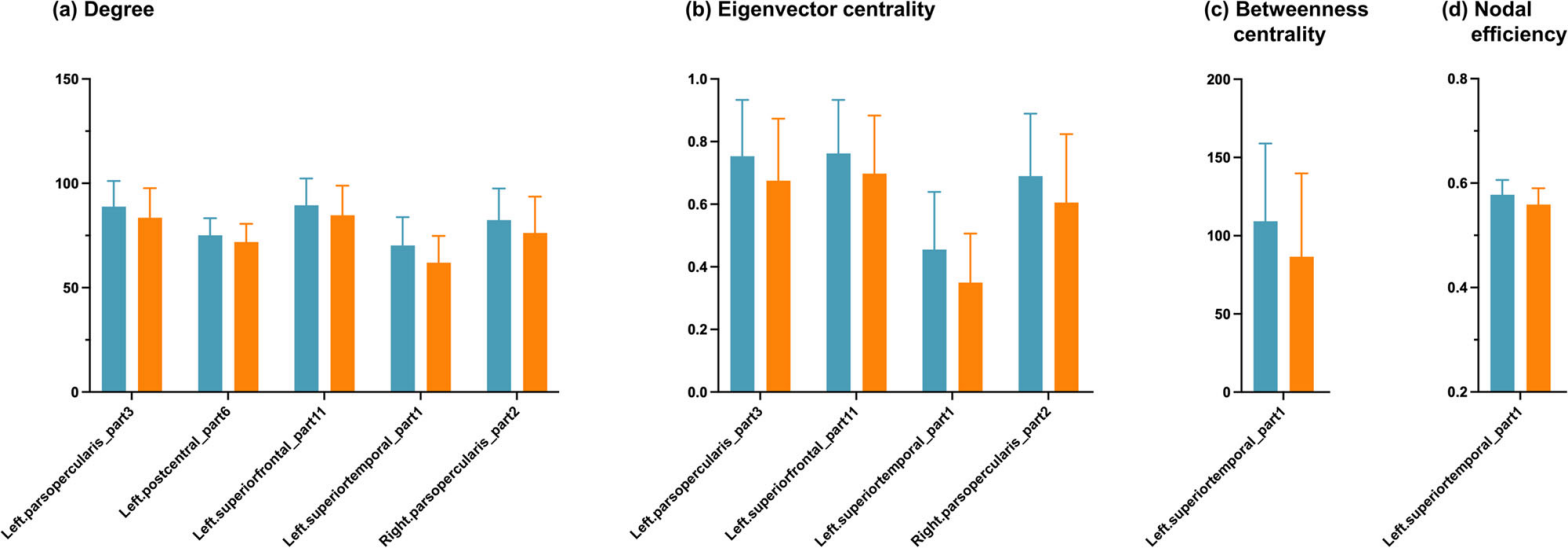
Group comparisons of nodal network measures of the morphometric similarity network. The blue and orange bars indicate the healthy control and schizophrenia patient groups, respectively. Group differences in **a** degree, **b** eigenvector centrality, **c** betweenness centrality, and **d** nodal efficiency are presented.

While the global efficiency significantly increased in the patient group, the local efficiency, modularity, and transitivity significantly decreased (Table 1). After correcting for multiple testing accounting for the number of cortical regions and nodal network measures, we identified 11 nodal network measures with significant group differences in individuals with schizophrenia compared with HCs (Fig. 3 and Supplementary Table 4). Individuals with schizophrenia had a significantly lower degree and eigenvector centrality in the left superior frontal (degree, t = −3.582, FDR *p* = 0.043; eigenvector centrality, t = −3.611, FDR *p* = 0.042), superior temporal (degree, t = −6.599, FDR *p* = < 0.001; eigenvector centrality, t = −6.713, FDR *p* = < 0.001), pars opercularis (degree, t = −4.050, FDR *p* = 0.013; eigenvector centrality, t = −4.144, FDR *p* = 0.010), and right pars opercularis areas (degree, t = −3.692, FDR *p* = 0.034; eigenvector centrality, t = −3.988, FDR *p* = 0.014) than HCs. The left postcentral area exhibited a lower degree (t = −3.859, FDR *p* = 0.020) in the patient group than in the control group. The left superior temporal area showed a lower betweenness centrality (t = −4.585, FDR *p* = 0.002) and nodal efficiency (t = −6.870, FDR *p* = <0.001) in the patient group than in the control group.

**Table 1:** Group comparisons of global network measures of morphometric similarity network.

Measure	Healthy control		Schizophrenia			
	Mean	SD	Mean	SD	*t*	FDR p
Global efficiency	0.590	0.003	0.591	0.003	2.649	0.017
Local efficiency	0.784	0.006	0.782	0.005	−2.719	0.017
Modularity	0.383	0.016	0.380	0.016	−1.981	0.048
Transivity	0.579	0.014	0.576	0.014	−2.305	0.029

*FDR* False discovery rate, *SD* Standard deviation.

### Associations with clinical symptoms, illness duration, antipsychotic dose, and cognitive function

**Fig. 4:**
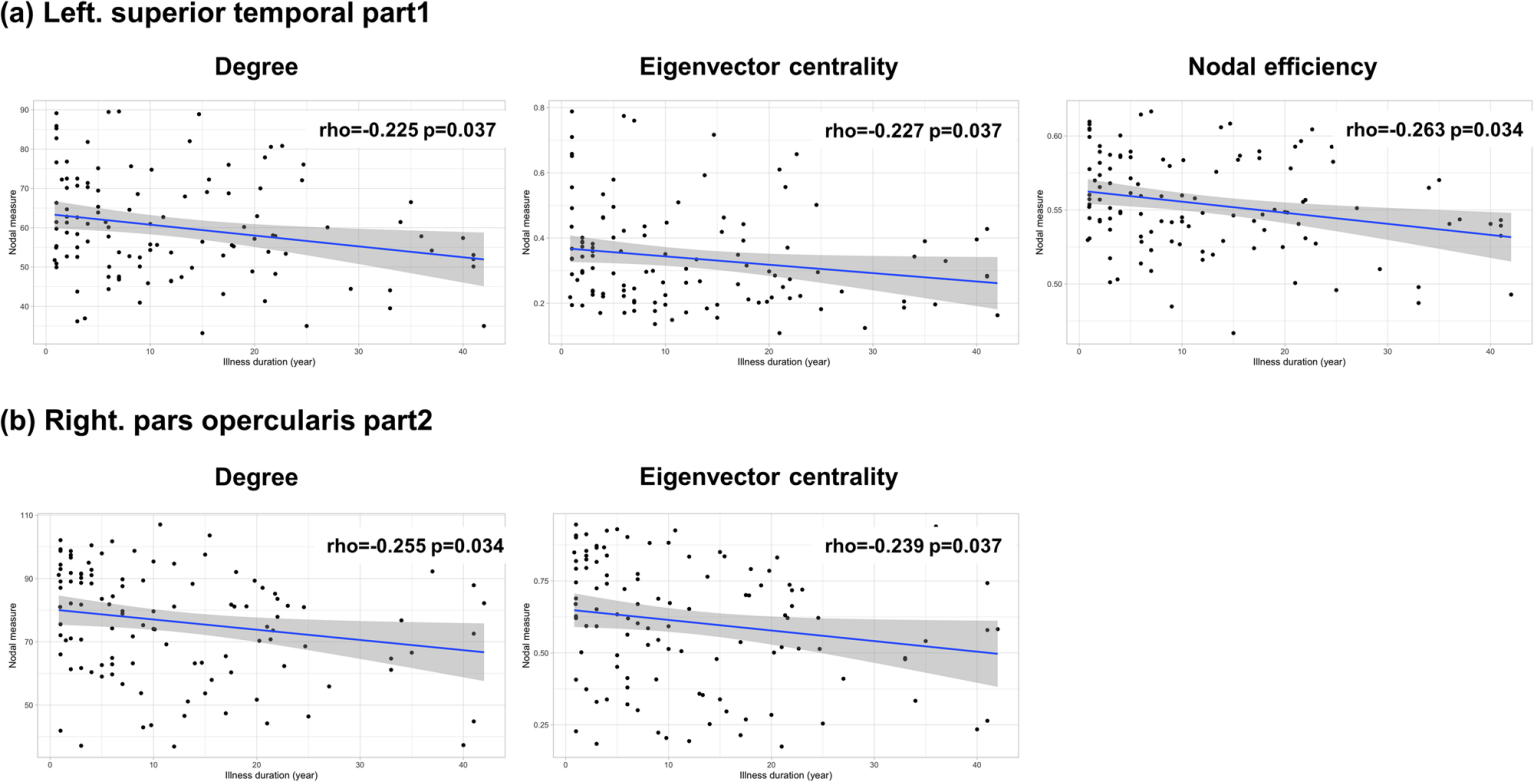
Associations of nodal measures with illness duration in individuals with schizophrenia. **a** In the left superior temporal region 1, the degree, eigenvector centrality, and nodal efficiency were negatively correlated with illness duration. **b** In the right par opercularis part 2, the degree and eigenvector centrality are negatively correlated with illness duration.

Supplementary Tables 5–8 display the associations between the mean MS of the cortical regions and subnetworks and the clinical variables. The mean MS of the left superior parietal area and frontoparietal subnetwork in the Yeo atlas was negatively associated with illness duration in the patient group. No global network measure was significantly associated with clinical variables in HCs or individuals with schizophrenia (Supplementary Tables 9 and 10). Moreover, no significant associations between nodal network measures and cognitive function were identified in the control group (Supplementary Table 11). The illness duration in the patient group was negatively associated with the degree and eigenvector centrality of the right pars opercularis area and the degree, eigenvector centrality, and nodal efficiency of the left superior temporal area (Supplementary Table 12 and Fig. 4). However, after adjusting for age, the associations of nodal network measures with illness duration did not remain significant (Supplementary Table 13).

## Discussion

In this study, we observed abnormalities in MS networks in individuals with schizophrenia compared with HCs at the node and network levels. At the node level, we observed 18 cortical regions with significant group differences in the mean MS between the patient and control groups. A lower mean MS was observed in the five subnetworks defined by the Von Economo or Yeo atlases in the patient group than in the control group. At the network level, several significant group differences were observed for the strength of connections between the cortical regions, with the left superior temporal area being involved in most of the significant connections, regardless of whether they were stronger or weaker in the patient group. Among the global network measures, while the global efficiency was greater in the patient group, the local efficiency, modularity, and transitivity were lower than those in the control group. Among several nodal network measures that were significantly lower in the patient group than in the control group, some nodal network measures of the right pars opercularis and left superior temporal areas were associated with illness duration in the patient group. Overall, our findings indicated the importance of the left superior temporal area in morphometric similarity networks of individuals with schizophrenia based on the pattern of connections to other cortical brain regions, abnormal nodal network measures, and their association with illness duration.

Among the 18 cortical regions with significant group differences in the mean MS, 14 cortical regions exhibited a low mean MS in the patient group. Morgan et al.^15^. reported significant group differences in the mean MS of 18 cortical regions between individuals with psychosis and HCs. The authors discovered that individuals with psychosis had a low mean MS in 15 cortical regions located in the superior frontal, caudal middle frontal, precentral, pars triangularis, and superior temporal areas. As we have replicated previous findings with a different neuroimaging dataset, an abnormal regional MS of the cerebral cortex in individuals with schizophrenia was confirmed. We also established that compared to the control group, the patient group exhibited a lower mean MS in five subnetworks: the somatosensory, dorsal attention, and limbic subnetworks and the primary motor cortex and association cortex 1. However, Morgan et al.^15^. revealed that individuals with psychosis had low mean MSs in different subnetworks to those reported in our study, namely, the association cortex, ventral attention, frontoparietal, and default mode subnetworks. Further studies are needed to address this inconsistency, given the considerable heterogeneity of morphometric features of the brain in individuals with psychosis^16,17^. We speculate that increased structural variance in the brain regions in the patient group partially accounted for the inconsistency.

We observed that individuals with schizophrenia had significantly stronger or weaker connections between cortical regions than HCs. Recently, normative modeling has been adopted to explain the heterogeneity of brain structures in individuals with schizophrenia^17,18^. Based on a reference normative range from HCs, individuals with schizophrenia exhibited both infra- and supranormal deviations of brain structures, although infra-normal deviations were common^18^. Our results may validate previous findings of both infra- and supranormal deviations of brain structures in individuals with schizophrenia. Notably, we observed that the left superior temporal area was involved in most of the significant connections between the cortical regions, regardless of the group comparisons. The left superior temporal area is reportedly a key region in the pathophysiology of schizophrenia, and this area is associated with disease course^19^, auditory hallucinations^20^, and formal thought disorders^21^. Our findings suggested the importance of the left superior temporal area in the structural network of the cerebral cortex, playing a central role in cortical connections.

Despite significant group differences in the global network measures, we believe that the implications may be limited because the group differences in actual values might be negligible. Instead, our results indicated regional abnormalities of the MS network in individuals with schizophrenia, with lower centrality values in several cortical regions, including the pars opercularis, superior frontal, and superior temporal areas. Structural abnormalities of the inferior^22,23^ and superior frontal gyrus^24^ and their relationships with cognitive function^25^ have been reported in individuals with schizophrenia. In line with our results, van den Heuvel et al.^5^. reported a reduced betweenness centrality of the frontal hubs and long node-specific path lengths of the frontal and temporal regions in individuals with schizophrenia. They suggested that a reduced central role of key frontal hubs might be linked to a limited structural capacity to integrate information in individuals with schizophrenia. However, contrary to our findings, Zhang et al. ^26^. demonstrated low nodal centrality of several cortical regions of the default mode network and high nodal centrality of the primary and paralimbic cortex regions in individuals with schizophrenia. Previous results on topological abnormalities in brain networks in individuals with schizophrenia have varied, which may be due to the use of different methods for constructing brain networks and the increased heterogeneity of morphometric features of brain regions in individuals with schizophrenia.

We observed an association between illness duration and centrality values of the right pars opercularis and left superior temporal areas in the patient group, which may indicate the detrimental effects of a prolonged disease period on the connectivity of the cortical regions.

Currently, there is lack of clarity on whether individuals with schizophrenia experience progressive brain changes. While individuals with poor outcomes tend to be more affected by the disease, exhibiting more pronounced changes in several brain regions, inconsistencies between previous findings necessitate further studies^27^. The associations of the left superior temporal and right inferior frontal areas with illness duration in our study are based on their connections to other cortical regions, not individual changes. Given the greater accountability of the morphometric similarity network for cytoarchitectonic similarity between cortical regions, further studies should be performed to investigate the longitudinal changes in cortical brain regions regarding their network properties. However, given the cross-sectional design of the current study, the interpretation should be verified, and long-term follow-up studies are needed to achieve confirmatory evidence on this issue.

We utilized various morphometric parameters of the cerebral cortex to construct MS networks in individuals with schizophrenia and HCs. In addition to the group comparisons of the mean MS of the cortical regions and subnetworks, we investigated the topological abnormalities of the MS networks and found that individuals with schizophrenia had regional abnormalities in several cortical regions in the MS network. The investigation of the MS network based on the network properties in the current study provided further evidence of the abnormal MS network in individuals with psychosis. Despite the novelty of investigating structural abnormalities of the cerebral cortex in schizophrenia with an advanced network model, our study had several limitations. First, we did not consider the effects of medication on the structural integrity of brain regions. Previous studies have reported the effects of antipsychotic treatment on structural changes in brain regions in individuals with schizophrenia^28,29^. Second, although we used a retrospective harmonization method for diffusion-weighted images and ComBat harmonization to adjust for site effects owing to the use of different neuroimaging datasets, nonbiological effects, such as scanner and image parameter effects, cannot be disregarded. Third, the schizophrenia group may not represent all individuals with schizophrenia, as this kind of neuroimaging study requires informed consent from participants. Fourth, the associations of nodal network measures with illness duration did not remain significant after adjusting for age, which suggested a cautious interpretation of the associations of nodal network measures with illness duration. This may be due to the strong relationship between age and illness duration, and future studies should consider complex interactions between age, illness duration, and nodal network measures.

We used T1-weighted and diffusion-weighted images of 239 HCs and 190 individuals with schizophrenia to investigate abnormalities in the MS network and their associations with the clinical symptoms of schizophrenia. Significant group differences in the mean MS of several cortical regions and subnetworks were observed. Individuals with schizophrenia had both stronger and weaker connections between cortical regions than HCs, and the left superior temporal area was involved in most of the significant connections. For the global network indices, we observed significant group differences, the implications of which might be limited owing to small effect sizes. Lower centrality values of the pars opercularis, superior frontal, and superior temporal areas were observed in the patient group, and associations with illness duration were observed in a few nodal network measures of the right pars opercularis and left superior temporal areas. Our findings indicate that the left superior temporal area may be a key region in the disrupted cortical brain network affected by schizophrenia, supported by its aberrant connections to other cortical regions, abnormal nodal network measures, and its relationship with illness duration. We demonstrated evidence of abnormalities in the structural networks in the brains of individuals with schizophrenia using a novel method involving MS networks, which could contribute to revealing the neurobiological background of schizophrenia.

## Materials and methods

### Study population

We utilized a neuroimaging dataset acquired from the Asan Medical Center (AMC), a tertiary hospital in the Republic of Korea. The demographic and clinical characteristics of the population in the AMC dataset are described in the Supplementary Materials. The Centers of Biomedical Research Excellence (COBRE) and Neuromorphometry by Computer Algorithm Chicago (NMorphCH) datasets were obtained via the SchizConnect database (http://schizconnect.org)^30^. Neuroimaging data from the University of California Los Angeles Consortium for Neuropsychiatric Phenomics LA5c Study (UCLA-CNP) were downloaded from OpenNeuro (http://openneuro.org) using the accession number ds000030. Brief information about the publicly available datasets is provided in the Supplementary Materials, while comprehensive details can be obtained elsewhere: COBRE^31^, NMorphCH (http://nunda.northwestern.edu/nunda/data/projects/NmorphCH), and UCLA-CNP^32,33^. The individual studies were approved by the local institutional review boards (IRBs) and conducted in accordance with the Declaration of Helsinki. The present study was approved by the IRB of the AMC (IRB No. 2021-0423).

The severity of the psychiatric symptoms of individuals with schizophrenia was evaluated using the Positive and Negative Syndrome Scale (PANSS)^34^ in the AMC and COBRE datasets. In the NMorphCH and UCLA-CNP datasets, the Scale for the Assessment of Positive Symptoms (SAPS)^35^ and the Scale for the Assessment of Negative Symptoms (SANS)^36^ were used. To ensure consistency in the symptom measurements, we used validated equations from a previous study^37^ to convert the SAPS and SANS scores into positive and negative PANSS scores. Considering the various neurocognitive tests utilized in the individual studies, we selected three specific tests: the full-scale intelligence quotient test, the color trail 1 test, and the word fluency test. These three tests were included in all neuroimaging datasets, and we could obtain standardized scores for a relatively large number of participants. The total antipsychotic dose at the date of the MRI scan was estimated as the daily olanzapine equivalent dose using validated conversion equations^38^.

The final study population included 239 HCs and 190 individuals with schizophrenia, and the demographic and clinical characteristics of the participants are presented in Table 2.

**Table 2:** Demographic and clinical characteristics of the study population.

	AMC		COBRE		NMorphCH		UCLA-CNP		Total		Statistic		
	HC	SCZ	HC	SCZ	HC	SCZ	HC	SCZ	HC	SCZ	t/ χ²	df	*p*
Number of subjects	23	47	72	57	32	40	112	46	239	190			
Age, mean (SD), y	30.3 (5.1)	28.6 (6.3)	37.8 (11.9)	38.6 (12.5)	31.9 (8.5)	32.6 (6.7)	31.4 (8.8)	35.8 (9.0)	33.3 (10.0)	34.2 (10.0)	−0.933	427	0.351
Male, *n* (%)	9 (39.1)	19 (40.4)	56 (77.8)	44 (77.2)	20 (62.5)	28 (70.0)	56 (50.0)	34 (73.9)	141 (59.0)	125 (65.8)	1.795	1	0.180
Duration of illness, mean (SD), y		3.5 (3.9)		16.3 (12.6)		14.3 (7.7)		NA					
Olanzapine equivalent dose^a^, mg/day		16.9 (11.0)		16.3 (12.8)		NA		20.4 (30.9)					
PANSS													
Total		62.1 (16.1)		65.5 (17.5)		NA		NA					
Positive		16.4 (7.5)		15.4 (5.2)		20.2 (6.8)		17.4 (4.7)					
Negative		16.7 (7.2)		17.8 (5.3)		21.8 (5.4)		16.4 (5.6)					
General		29.0 (7.7)		32.3 (8.1)		NA		NA					

*AMC* Asan Medical Center, *COBRE* Centers of Biomedical Research Excellence, *df* degrees of freedom, *HC* healthy control, *NA* not available, *NMorphCH* Neuromorphometry by Computer Algorithm Chicago, *SCZ* schizophrenia, *UCLA-CNP* University of California Los Angeles Consortium for Neuropsychiatric Phenomic LA5c Study.

^a^The number of patients who had available information on medication is 46 in AMC, 56 in COBRE, and 43 in UCLA-CNP.

### Image acquisition and preprocessing

Information on the scanners and image parameters is presented in Supplementary Table 1. We visually inspected T1- and diffusion-weighted images to identify any signal dropouts or artifacts. In addition, SliceDiffusionQC (https://github.com/pnlbwh/SlicerDiffusionQC) was used for quality control of the diffusion-weighted images. The details of the quality control procedures are described in the Supplementary Materials. Participants whose images were not eligible for downstream image analyses were excluded. Preprocessing of diffusion-weighted images was performed using the Psychiatry Neuroimaging Laboratory pipeline (https://github.com/pnlbwh/pnlutil), which involves axis alignment, centering, and correction for eddy current and head motion. According to previous studies, a retrospective harmonization method was used to minimize site differences in the acquisition of diffusion-weighted images^39,40^ that utilized raw images before further image analysis. Details on the retrospective harmonization procedures are described in the Supplementary Materials.

### Image analysis

The following procedures were conducted to obtain 10 MRI parameters for each cortical region. First, the FreeSurfer automated pipeline (version 7.1)^41^ was utilized (recon-all command). This process involved the extraction of surface area, gray matter volume, mean and standard deviation of thickness, and Gaussian and mean curvature, as well as the folding and curvature indices of the cerebral cortex. Next, nonlinear registration was performed to align the FreeSurfer parcellated cortical regions with diffusion-weighted images using Advanced Normalization Tools (version 2.3.4)^42^. Subsequently, the DTIFIT tool from FMRIB’s Diffusion Toolbox was employed to estimate the mean fractional anisotropy and diffusivity. Although it is still uncertain which are the determinants of diffusion measures in the cerebral cortex^43^, several ex vivo studies have reported a good correspondence of diffusion measures to cortical histology^44–46^ which is barely represented in conventional MRI parameters. In line with previous studies adopting diffusion indices to investigate cortical gray matter abnormalities in neuropsychiatric disorders^47,48^, including schizophrenia^49^, we utilized diffusion measures to capture microscopic changes that frequently predate any structural changes. The modified Desikan–Killiany atlas, which consists of 308 cortical regions, was adopted to parcellate the cerebral cortex^50^. This process resulted in 10 MRI parameters for each of the 308 cortical regions in each participant. ComBat harmonization was used to adjust nonbiological variances across individual studies, such as scanner effects. We utilized neuroCombat^51^ with the covariates of age, sex, and group. The site differences in most MRI parameters decreased after the ComBat harmonization, although some residual differences between the study sites were observed. The validation process for the harmonization procedures is described in the Supplementary Materials. Given the different scales across the 10 image parameters, we normalized each parameter into a z score. We then calculated MS as Pearson’s correlation for each pair of morphometric feature vectors and merged them into a 308 × 308 MS matrix for each participant.

### Morphometric similarity at the node level

Before constructing cortical networks based on the MS of the cortical regions, we investigated regional MS abnormalities in individuals with schizophrenia compared with HCs. First, we computed the mean MS of the cortical regions by averaging the MS of the remaining 307 cortical regions for each cortical region. Second, we utilized the Von Economo and Yeo atlases, which are based on the cytoarchitectonic criteria^13^ and resting-state functional MRI networks, respectively, to delineate subnetworks from the cerebral cortex^52,53^. We calculated the average MS of the cortical regions included in each subnetwork, as defined by the Von Economo or Yeo atlases.

### Morphometric similarity at the network level

We used the network-based statistic method^54^ to investigate group differences in the strength of connections between the cortical regions in the MS matrix. We set the initial threshold as a *p* value of 0.0000011, which was based on the Bonferroni correction for all cortical region connections (*n* = 47,228). We performed group comparisons to reveal connections that exhibited significantly stronger or weaker strengths in individuals with schizophrenia than in HCs, with the covariates of age, sex, and their interaction.

A range of sparsity thresholds from 0.05 to 0.45 with an interval of 0.02 was applied to the MS matrix. We applied the following criteria to determine the threshold range: 1) the threshold networks should be estimable for the small-worldness scalar, and 2) the small-worldness scalar of the threshold networks of all participants should be greater than 1.1, which was based on previous studies on the construction of binarized networks using the small-worldness scalar^55,56^. From 0.10 to 0.40 of the sparsity thresholds with an interval of 0.02, we calculated global and nodal network measures of the MS networks using the BrainGraph (https://github.com/cwatson/brainGraph) and NetworkToolbox^57^ packages. We averaged the network measures calculated at each sparsity threshold to compare groups. We used global efficiency, modularity, transitivity, and local efficiency as global network measures and degree, betweenness centrality, eigenvector centrality, and nodal efficiency as the nodal network measures.

### Statistical analysis

All the statistical analyses were performed using R software (ver. 4.0.2; R Development Core Team, Vienna, Austria), and significance was based on an alpha value less than 0.05. Student’s *t* tests or chi-square tests were used to compare continuous or categorical variables related to the demographic and clinical characteristics of the population. We used linear regressions with the covariates of age, sex, and their interaction in group comparisons for the mean MS of the cortical regions and subnetworks and the global and nodal network measures. Spearman’s correlation was used to analyze associations of MS with clinical variables, which were the PANSS positive and negative scores, cognitive function, illness duration, and antipsychotic dose.

We used the Benjamini–Hochberg method to adjust the multiple testing and determined the significance based on a false discovery rate of q < 0.05. In the group comparisons, we accounted for the number of cortical regions (*n* = 308), subnetworks (*n* = 14), and global network measures (*n* = 4). Group comparisons of nodal network measures were performed by adjusting for the number of cortical regions and the number of nodal network measures (*n* = 308 × 4). In associations with clinical variables, multiple testing correction was performed for each clinical variable. We only included the significant cortical regions, subnetworks, or network properties in the group comparisons.

### Supplementary information

Supplementary Material

## Data Availability

The data of this study are available from the corresponding author upon reasonable request.
