# Supplementary material for: Topological abnormalities of the morphometric similarity network of the cerebral cortex in schizophrenia
Source: Schizophrenia (Heidelb). 2024 Jun 17;10(1):57. doi: 10.1038/s41537-024-00477-x (PMC11183129; doi:10.1038/s41537-024-00477-x)
Supplement: Supplementary file 1 — Supplementary Material [file 41537_2024_477_MOESM1_ESM.docx]

Supplementary Material

[Supplementary Methods 2](#_Toc164420557)

[Supplementary Table 1. Information on scanners and image parameters 7](#_Toc164420558)

[Supplementary Table 2. Group comparisons of the mean morphometric similarity of cortical regions 8](#_Toc164420559)

[Supplementary Table 3. Group comparisons of the mean morphometric similarity of subnetworks 16](#_Toc164420560)

[Supplementary Table 4. Group comparisons of nodal measures of the morphometric similarity network 17](#_Toc164420561)

[Supplementary Table 5. Associations of the mean morphometric similarity of cortical regions with cognitive functions in healthy controls 61](#_Toc164420562)

[Supplementary Table 6. Associations of the mean morphometric similarity of cortical regions with clinical symptoms, illness duration, antipsychotic dose, and cognitive function in patients with schizophrenia 62](#_Toc164420563)

[Supplementary Table 7. Associations of the mean morphometric similarity of subnetworks with cognitive functions in healthy controls 63](#_Toc164420564)

[Supplementary Table 8. Associations of the mean morphometric similarity of subnetworks with clinical symptoms, illness duration, antipsychotic dose, and cognitive functions in patients with schizophrenia 64](#_Toc164420565)

[Supplementary Table 9. Associations of the global measures of the morphometric similarity network with cognitive functions in healthy controls 65](#_Toc164420566)

[Supplementary Table 10. Associations of the global measures of the morphometric similarity network with clinical symptoms, illness duration, antipsychotic dose, and cognitive function in patients with schizophrenia 66](#_Toc164420567)

[Supplementary Table 11. Associations of the nodal measures of the morphometric similarity network with cognitive functions in healthy controls 67](#_Toc164420568)

[Supplementary Table 12. Associations of the nodal measures of the morphometric similarity network with clinical symptoms, illness duration, antipsychotic dose, and cognitive function in patients with schizophrenia 69](#_Toc164420569)

[Supplementary Table 13. Associations of the nodal measures of the morphometric similarity network with illness duration as a covariate of age 72](#_Toc164420570)

[Supplementary Figure 1. Group comparisons of the mean morphometric similarity of subnetworks. 73](#_Toc164420571)

[References 74](#_Toc164420572)

# **Supplementary Methods**

**Study Population**

**Asan Medical Center**

All participants were right-handed, aged between 20 and 40 years, and ideally did not have any illnesses that could affect brain functioning. Patients with schizophrenia were diagnosed by a board-certified psychiatrist according to the Diagnostic and Statistical Manual of Mental Disorders-IV Text Revision (DSM-IV-TR). The patients did not have any comorbid psychiatric diagnoses, and the duration of psychotic symptoms was less than 5 years. Patients whose psychiatric diagnoses changed within 1–6 months of the initial diagnosis were excluded. The severity of psychiatric symptoms was assessed using the Positive and Negative Syndrome Scale within a week of the magnetic resonance imaging (MRI) scan. According to the DSM-IV-TR, the control participants and their first-degree relatives did not have any Axis I psychiatric diagnoses.

**Centers of Biomedical Research Excellence**

These multimodal neuroimaging data were obtained from patients with schizophrenia and age-matched controls and were released through the collaborative informatics and neuroimaging suite of the Mind Research Network. The Structured Clinical Interview for DSM-IV was used to diagnose schizophrenia. The control participants had no personal or family history of major psychiatric disorders. Among the eligible participants, those with an IQ<70; a history of neurological disorders, severe head trauma, substance abuse, or dependence within the last 12 months; and MRI contraindications were excluded.

**Neuromorphometry by computer algorithm Chicago**

A longitudinal study with a 2-year follow-up was conducted at Northwestern University and included clinical, cognitive, and neuroimaging data from patients with schizophrenia and healthy controls. The neuroimaging data included T1-, T2-, and diffusion-weighted images; resting-state functional MRI (fMRI) data; and an n-back fMRI task. The clinical symptoms of patients with schizophrenia were assessed using the Scale for the Assessment of Positive Symptoms and Scale for the Assessment of Negative Symptoms. Neurocognitive information on working memory, episodic memory, and executive function of the participants was included in the dataset.

**University of California Los Angeles Consortium for Neuropsychiatric Phenomics LA5c Study**

This publicly available dataset includes neuroimaging data of patients with schizophrenia, bipolar disorder, or attention-deficit hyperactivity disorder and healthy controls with neuropsychological and neurocognitive data. The primary goal of this project was to understand the dimensional structure of memory and cognitive control function in nonpatient and patient samples. We selectively retrieved clinical and neuroimaging data from patients with schizophrenia and healthy individuals from the entire dataset.

**Quality assessment of MRI data**

The T1- and diffusion-weighted images were visually inspected to detect any signal dropouts or artifacts. For the diffusion-weighted images, we used SliceDiffusionQC (https://github.com/pnlbwh/SlicerDiffusionQC) to remove poor gradient volumes from the diffusion-weighted images. Each diffusion volume was classified as a good or a bad gradient based on the distance to a median line estimated from Kullback–Leibler divergence between consecutive diffusion volumes. After automatic software processing, we carefully reviewed the classification results and discarded bad gradient volumes.

**Harmonization**

**A retrospective harmonization method for diffusion-weighted images**

Retrospective harmonization of the raw dMRI data was performed according to previous studies^2,3^. Initially, we selected 20 right-handed participants from the UCLA-CNP, which was determined as the reference dataset based on the image parameters and sample size. A subset of 20 participants from each target site (AMC, COBRE, and NMorphCH), matched for age, sex, and handedness to the greatest extent, was also selected. Each subset comprised 20 healthy controls. Below, an unpaired *t* test was used to assess the matching.

| Variable | Site | | *t/χ2* | *df* | *p* |
| --- | --- | --- | --- | --- | --- |
|  | UCLA-CNP | AMC |  |  |  |
| Age, year, mean (SD) | 29.5 (8.2) | 29.5 (5.4) | 0.000 | 38 | 1.000 |
| Sex, male (%) | 7 (35) | 7 (35) | 0.000 | 1 | 1.000 |
|  |  |  |  |  |  |
| Variable | Site | | *t/χ2* | *df* | *p* |
|  | UCLA-CNP | COBRE |  |  |  |
| Age, year, mean (SD) | 29.0 (7.4) | 29.0 (7.5) | 0.000 | 38 | 1.000 |
| Sex, male (%) | 8 (40) | 8 (40) | 0.000 | 1 | 1.000 |
|  |  |  |  |  |  |
| Variable | Site | | *t/χ2* | *df* | *p* |
|  | UCLA-CNP | NMorphCH |  |  |  |
| Age, year, mean (SD) | 31.5 (9.2) | 31.5 (9.3) | 0.000 | 38 | 1.000 |
| Sex, male (%) | 10 (50) | 10 (50) | 0.000 | 1 | 1.000 |

For harmonization of the raw dMRI data, we used dMRIharmonization (https://github.com/pnlbwh/dMRIharmonization). Using the dMRI data of the matched subsets, we created scale maps, which were generated from pairs of rotation-invariant spherical harmonics feature templates. The scanner-specific differences between the reference and target sites were determined by building scale maps. Then, the scale maps were applied to the raw dMRI data of the target site, resulting in harmonized dMRI data. The default options implemented in the program were used for this procedure. To validate the performance of the harmonization, we used the Illinois Institute of Technology Human Brain Atlas^4^ and calculated the mean FA value over a whole-brain white matter skeleton (IITmean_FA_skeleton.nii.gz) before and after harmonization. An unpaired *t* test was used to evaluate intersite differences. The below figure showed that although intersite differences decreased after harmonization (AMC vs target site, before: 0.450±0.014 vs 0.495±0.026, *t*=-9.914, *p*=<0.001; after: 0.450±0.014 vs 0.479±0.026, *t*=-6.196, *p*=<0.001), a significant difference still remained.


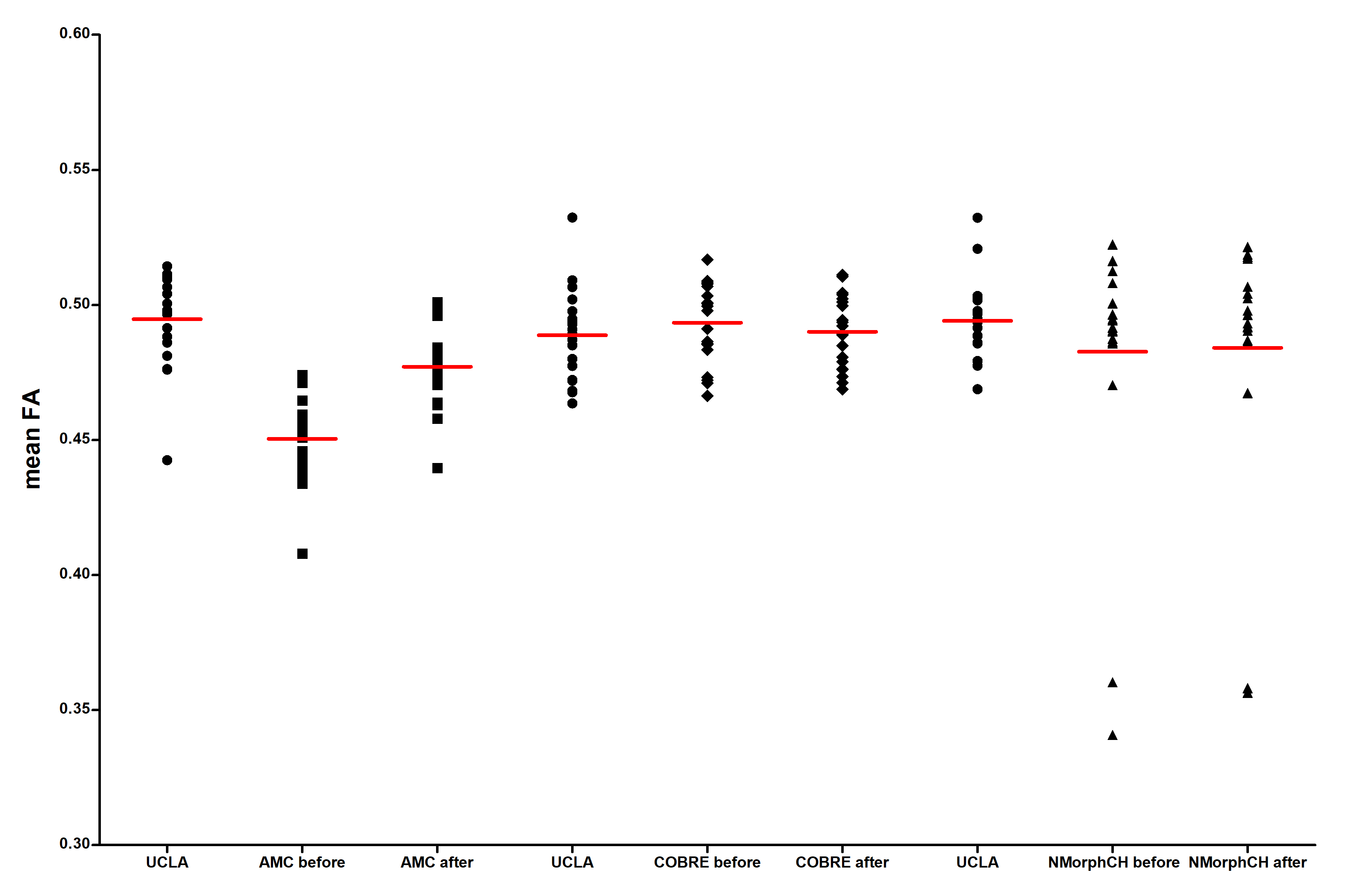


We applied b-value mapping, resampling, and gibbs unringing to the raw dMRI data of the reference site. The mean FA value was computed using the resampled dMRI data of the reference site and the harmonized dMRI data of the target sites. Outliers, defined by a 1.5 interquartile range (IQR) below the first quartile of the FA value, were excluded from the downstream processes (Please see the below figure).


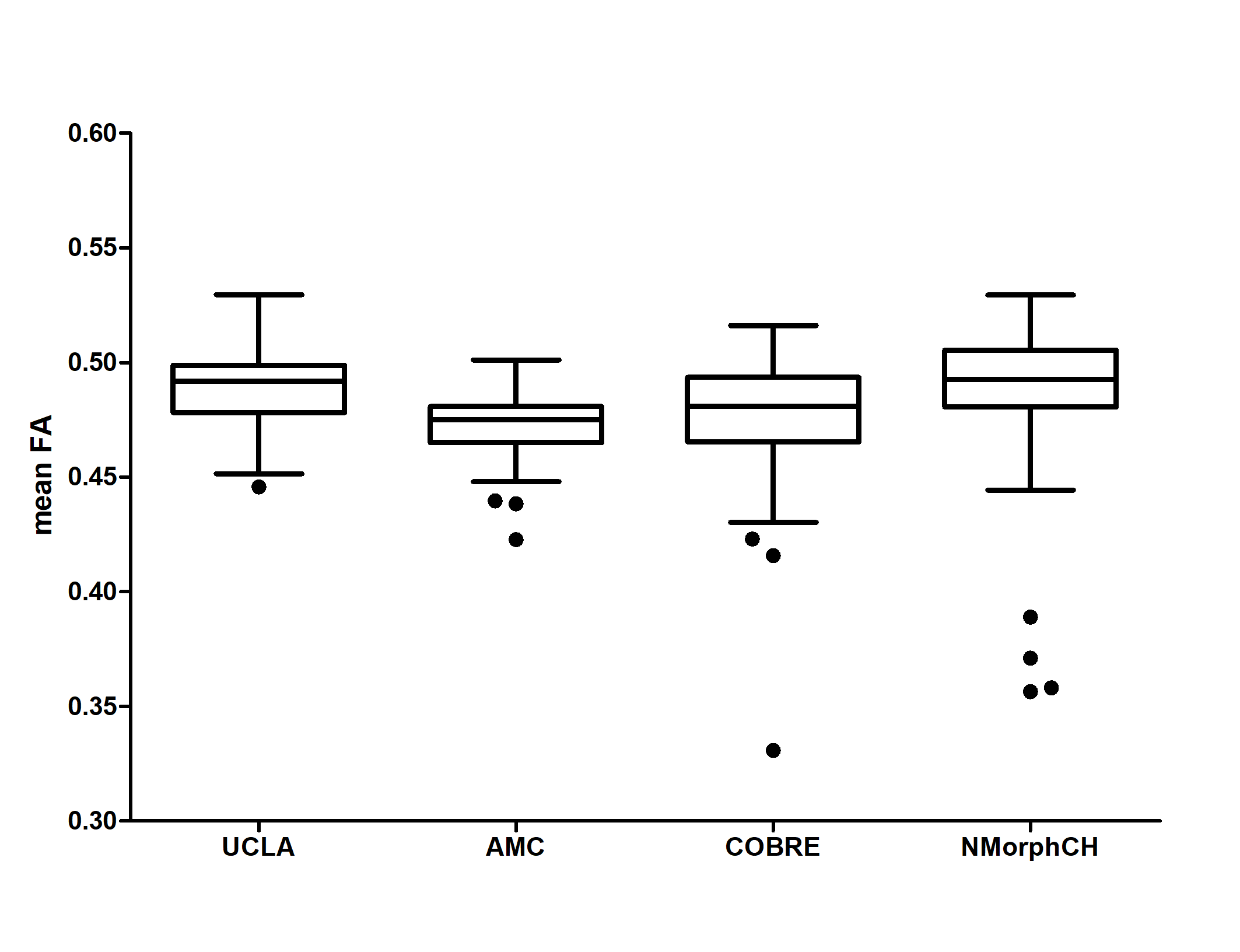


**ComBat harmonization**

We performed Combat harmonization to adjust nonbiological variances across individual studies for each MRI parameters. We used healthy control samples from each study site that were matched for age and sex. Below, we show the characteristics of the matched samples and the statistics for group comparisons.

|  | Reference | Target | Statistic | |
| --- | --- | --- | --- | --- |
|  | AMC | COBRE | t or chi | p |
| Age, mean (SD), year | 29.2 (4.8) | 30.1 (5.3) | -0.518 | 0.608 |
| Male, n (%) | 8 (53.3) | 12 (63.2) | 0.052 | 0.82 |
|  | AMC | NMorphCH | t or chi | p |
| Age, mean (SD), year | 27.1 (3.2) | 27.8 (3.3) | -0.504 | 0.619 |
| Male, n (%) | 5 (41.7) | 8 (66.7) | 0.671 | 0.413 |
|  | AMC | UCLA-CNP | t or chi | p |
| Age, mean (SD), year | 29.7 (4.9) | 28.2 (4.5) | 1.204 | 0.236 |
| Male, n (%) | 9 (42.9) | 20 (48.8) | 0.03 | 0.862 |
|  | COBRE | NMorphCH | t or chi | p |
| Age, mean (SD), year | 28.8 (7.6) | 30.7 (8.3) | -0.792 | 0.433 |
| Male, n (%) | 20 (80.0) | 16 (76.2) | <0.001 | 1 |
|  | COBRE | UCLA-CNP | t or chi | p |
| Age, mean (SD), year | 32.9 (9.3) | 30.0 (8.2) | 1.745 | 0.084 |
| Male, n (%) | 37 (75.5) | 47 (62.7) | 1.688 | 0.194 |
|  | NMorphCH | UCLA-CNP | t or chi | p |
| Age, mean (SD), year | 29.0 (6.7) | 26.6 (5.5) | 1.481 | 0.147 |
| Male, n (%) | 11 (47.8) | 21 (42.0) | 0.045 | 0.832 |

We averaged each MRI parameter from 308 ROIs and performed an unpaired t test to evaluate residual site effects on the MRI parameters. The figure below shows the t statistics for site comparisons, and site differences decreased in most MRI parameters after the ComBat harmonization despite some site differences.


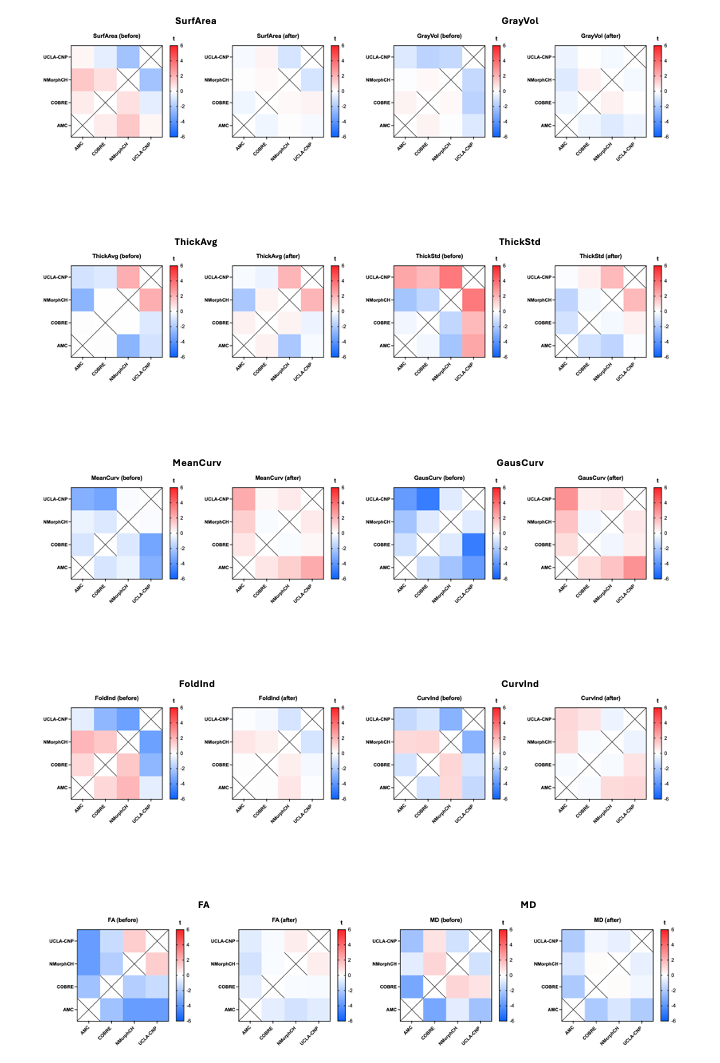


# **Supplementary Table 1. Information on scanners and image parameters**

| Study | Sequence | Scanners | Protocol parameters | |
| --- | --- | --- | --- | --- |
| AMC | T1 | 3T Philips Achieva | T1 images were acquired with turbo field echo sequence, with the following scan parameters: field of view (FOV), 240*240*170; voxel size, 1*1*1 mm^3^; echo time (TE), 4.6 ms; repetition time (TR), 9 ms; flip angle, 8°. | |
| AMC | DWI | 3T Philips Achieva | | The diffusion-weighted images were acquired via echo-planar imaging (EPI) sequence. One baseline (b=0) image and 32 diffusion gradient directions with b=1000 s/mm^2^ were acquired. The scan parameters were as follows: FOV, 224*224*135 mm; voxel size, 2*2*3 mm^3^; TE, 70 ms; flip angle, 90°; and TR, 5422 ms. |
| COBRE | T1 | 3T Siemens Trio | Coronal T1-weighted structural images were acquired with a 12-channel head-coil and a five-echo MPRAGE sequence (TE = 1.64, 3.5, 5.36, 7.22, and 9.08 ms; TR = 2.53 s; TI = 1.2 s, flip angle = 7°; number of excitations = 1; slice thickness = 1 mm; field of view = 256 mm; resolution = 256 × 256). | |
| COBRE | DWI | 3T Siemens Trio | Diffusion weighted images were acquired with a voxel size of 2.0 × 2.0 × 2.0 mm (TR = 9000 ms; TE = 84 ms; B values of 0 and 800; bandwidth = 1562; 72 slices and 35 directions). | |
| NMorphCH | T1 | 3T Siemens Trio | Coronal T1-weighted structural images were acquired with a 32-channel head coil and a MPRAGE sequence (TR = 2400 ms, TE = 3.16 ms, flip = 8°, 256 × 256 matrix, 176 slices, slice thickness = 1 mm). | |
| NMorphCH | DWI | 3T Siemens Trio | Diffusion weighted images were acquired with a slice thickness of 2 mm (TR = 8000 ms, TE = 86 ms, B values of 0 and 800; flip = 90°, 896 × 896 matrix, 35 slices and 30 directions). | |
| UCLA-CNP | T1 | 3T Siemens Trio | The scan parameters for the T1-weighted high-resolution anatomical scan (MPRAGE) were as follows: slice thickness, 1 mm; 176 slices; repetition time, 1900 ms; TE, 2.26 ms; matrix, 256 × 256; and FOV, 250 × 250 mm. | |
| UCLA-CNP | DWI | 3T Siemens Trio | The parameters for the DWI were as follows: slice thickness, 2 mm; 64 directions; TR/TE, 9000/93 ms; flip angle, 90°; matrix, 96 × 96; axial slices, b = 0 and 1000 s/mm^2^. | |

AMC: Asan Medical Center, DW: diffusion-weighted, COBRE: Centers of Biomedical Research Excellence, NMorphCH: Neuromorphometry by Computer Algorithm Chicago, UCLA-CNP: University of California Los Angeles Consortium for Neuropsychiatric Phenomic LA5c Study

# **Supplementary Table 2. Group comparisons of the mean morphometric similarity of cortical regions**

|  | Healthy control | | Schizophrenia | |  |  |
| --- | --- | --- | --- | --- | --- | --- |
| Structure | Mean | SD | Mean | SD | Unadjusted p | FDR adjusted p |
| Left.bankssts_part1 | 0.03421 | 0.03238 | 0.03377 | 0.03052 | 0.896 | 0.957 |
| Left.bankssts_part2 | 0.04305 | 0.02861 | 0.04402 | 0.02663 | 0.563 | 0.785 |
| Left.caudalanteriorcingulate_part1 | -0.00366 | 0.025 | -5.00E-05 | 0.02409 | 0.086 | 0.312 |
| Left.caudalmiddlefrontal_part1 | 0.04321 | 0.0243 | 0.04121 | 0.02455 | 0.354 | 0.612 |
| Left.caudalmiddlefrontal_part2 | 0.05017 | 0.02358 | 0.04094 | 0.02639 | <0.001 | 0.01 |
| Left.caudalmiddlefrontal_part3 | 0.0388 | 0.02008 | 0.03826 | 0.02055 | 0.852 | 0.93 |
| Left.caudalmiddlefrontal_part4 | 0.03941 | 0.02603 | 0.03793 | 0.02421 | 0.54 | 0.777 |
| Left.cuneus_part1 | -0.01436 | 0.0199 | -0.01333 | 0.01886 | 0.522 | 0.776 |
| Left.cuneus_part2 | -0.03944 | 0.02324 | -0.03714 | 0.02268 | 0.333 | 0.594 |
| Left.entorhinal_part1 | -0.04411 | 0.01603 | -0.0433 | 0.016 | 0.683 | 0.85 |
| Left.frontalpole_part1 | -0.02922 | 0.0224 | -0.02585 | 0.02083 | 0.144 | 0.386 |
| Left.fusiform_part1 | 0.01976 | 0.02551 | 0.02034 | 0.02365 | 0.743 | 0.89 |
| Left.fusiform_part2 | -0.01638 | 0.02484 | -0.0165 | 0.02515 | 0.984 | 0.992 |
| Left.fusiform_part3 | -0.01276 | 0.02698 | -0.01489 | 0.02636 | 0.589 | 0.803 |
| Left.fusiform_part4 | -0.00412 | 0.0263 | -0.00473 | 0.02703 | 0.904 | 0.957 |
| Left.fusiform_part5 | 0.01116 | 0.02895 | 0.00989 | 0.02624 | 0.774 | 0.907 |
| Left.inferiorparietal_part1 | 0.00464 | 0.03185 | 0.00041 | 0.02955 | 0.136 | 0.386 |
| Left.inferiorparietal_part2 | -0.02557 | 0.02779 | -0.02232 | 0.0261 | 0.25 | 0.514 |
| Left.inferiorparietal_part3 | 0.03435 | 0.0258 | 0.03058 | 0.02589 | 0.151 | 0.386 |
| Left.inferiorparietal_part4 | 0.00108 | 0.03076 | -0.00297 | 0.02808 | 0.15 | 0.386 |
| Left.inferiorparietal_part5 | 0.00489 | 0.02852 | 0.00872 | 0.02765 | 0.096 | 0.337 |
| Left.inferiorparietal_part6 | -0.01511 | 0.02984 | -0.01701 | 0.02771 | 0.451 | 0.7 |
| Left.inferiorparietal_part7 | -0.02781 | 0.02698 | -0.02279 | 0.02684 | 0.068 | 0.28 |
| Left.inferiorparietal_part8 | 0.00503 | 0.03017 | 0.0043 | 0.0252 | 0.687 | 0.85 |
| Left.inferiortemporal_part1 | -0.01706 | 0.02773 | -0.01307 | 0.02926 | 0.2 | 0.446 |
| Left.inferiortemporal_part2 | 0.00025 | 0.03276 | -0.0064 | 0.03092 | 0.033 | 0.169 |
| Left.inferiortemporal_part3 | -0.02865 | 0.02961 | -0.02956 | 0.02584 | 0.773 | 0.907 |
| Left.inferiortemporal_part4 | -0.00327 | 0.03199 | -0.0112 | 0.03077 | 0.01 | 0.092 |
| Left.inferiortemporal_part5 | -0.03376 | 0.02891 | -0.02935 | 0.02761 | 0.13 | 0.386 |
| Left.inferiortemporal_part6 | -0.02283 | 0.02921 | -0.02431 | 0.02843 | 0.667 | 0.85 |
| Left.insula_part1 | 0.02638 | 0.02323 | 0.02741 | 0.02301 | 0.681 | 0.85 |
| Left.insula_part2 | 0.01307 | 0.024 | 0.01068 | 0.02465 | 0.287 | 0.549 |
| Left.insula_part3 | 0.00852 | 0.02899 | 0.00734 | 0.02925 | 0.557 | 0.779 |
| Left.insula_part4 | 0.03093 | 0.02499 | 0.03028 | 0.02394 | 0.811 | 0.912 |
| Left.isthmuscingulate_part1 | -0.01708 | 0.02601 | -0.01091 | 0.02601 | 0.014 | 0.112 |
| Left.isthmuscingulate_part2 | -0.03365 | 0.02136 | -0.0315 | 0.0225 | 0.267 | 0.534 |
| Left.lateraloccipital_part1 | -0.02873 | 0.03098 | -0.02498 | 0.02916 | 0.228 | 0.493 |
| Left.lateraloccipital_part2 | -0.01875 | 0.03156 | -0.02 | 0.02839 | 0.911 | 0.959 |
| Left.lateraloccipital_part3 | -0.05258 | 0.02382 | -0.04891 | 0.02268 | 0.117 | 0.382 |
| Left.lateraloccipital_part4 | -0.02941 | 0.02563 | -0.03087 | 0.02502 | 0.556 | 0.779 |
| Left.lateraloccipital_part5 | -0.05967 | 0.02365 | -0.05992 | 0.02159 | 0.924 | 0.964 |
| Left.lateraloccipital_part6 | -0.05067 | 0.02461 | -0.04805 | 0.02492 | 0.176 | 0.423 |
| Left.lateraloccipital_part7 | -0.01391 | 0.02619 | -0.0125 | 0.02455 | 0.548 | 0.777 |
| Left.lateraloccipital_part8 | -0.05725 | 0.02322 | -0.05763 | 0.02278 | 0.997 | 0.998 |
| Left.lateraloccipital_part9 | -0.04187 | 0.02366 | -0.04053 | 0.02302 | 0.471 | 0.711 |
| Left.lateralorbitofrontal_part1 | -0.05012 | 0.02131 | -0.04371 | 0.02152 | 0.001 | 0.029 |
| Left.lateralorbitofrontal_part2 | -0.04089 | 0.02445 | -0.03298 | 0.0252 | 0.001 | 0.021 |
| Left.lateralorbitofrontal_part3 | -0.02217 | 0.02583 | -0.01904 | 0.02655 | 0.191 | 0.437 |
| Left.lateralorbitofrontal_part4 | -0.00839 | 0.02863 | -0.00449 | 0.02724 | 0.169 | 0.414 |
| Left.lingual_part1 | -0.00384 | 0.026 | -0.00183 | 0.02654 | 0.317 | 0.584 |
| Left.lingual_part2 | -0.0368 | 0.02591 | -0.03585 | 0.02352 | 0.735 | 0.885 |
| Left.lingual_part3 | -0.01295 | 0.02637 | -0.01501 | 0.02419 | 0.381 | 0.636 |
| Left.lingual_part4 | -0.01836 | 0.02013 | -0.01672 | 0.01874 | 0.408 | 0.663 |
| Left.lingual_part5 | -0.01254 | 0.02063 | -0.01048 | 0.02059 | 0.321 | 0.588 |
| Left.lingual_part6 | -0.02808 | 0.0221 | -0.02509 | 0.02179 | 0.134 | 0.386 |
| Left.medialorbitofrontal_part1 | -0.03969 | 0.02672 | -0.03182 | 0.02746 | 0.004 | 0.056 |
| Left.medialorbitofrontal_part2 | -0.01786 | 0.03029 | -0.01504 | 0.03082 | 0.282 | 0.544 |
| Left.medialorbitofrontal_part3 | -0.03094 | 0.02633 | -0.02668 | 0.02806 | 0.062 | 0.26 |
| Left.middletemporal_part1 | -0.00402 | 0.02591 | -0.00225 | 0.02545 | 0.547 | 0.777 |
| Left.middletemporal_part2 | -0.02205 | 0.02538 | -0.01837 | 0.02509 | 0.132 | 0.386 |
| Left.middletemporal_part3 | 0.00366 | 0.02552 | 0.00936 | 0.02418 | 0.021 | 0.138 |
| Left.middletemporal_part4 | -0.00943 | 0.0236 | -0.00775 | 0.02546 | 0.469 | 0.711 |
| Left.middletemporal_part5 | 0.00814 | 0.02998 | 0.00716 | 0.03054 | 0.846 | 0.928 |
| Left.paracentral_part1 | 0.03323 | 0.02241 | 0.02735 | 0.02359 | 0.01 | 0.092 |
| Left.paracentral_part2 | 0.03815 | 0.0238 | 0.0321 | 0.02621 | 0.019 | 0.133 |
| Left.paracentral_part3 | 0.03014 | 0.02844 | 0.0198 | 0.02818 | <0.001 | 0.015 |
| Left.parahippocampal_part1 | 0.01323 | 0.0235 | 0.01371 | 0.02237 | 0.786 | 0.91 |
| Left.parahippocampal_part2 | 0.0165 | 0.02404 | 0.01688 | 0.02201 | 0.998 | 0.998 |
| Left.parsopercularis_part1 | 0.03067 | 0.0321 | 0.02939 | 0.02834 | 0.575 | 0.794 |
| Left.parsopercularis_part2 | 0.05152 | 0.02286 | 0.04929 | 0.02073 | 0.303 | 0.569 |
| Left.parsopercularis_part3 | 0.05713 | 0.02472 | 0.0535 | 0.02118 | 0.105 | 0.359 |
| Left.parsorbitalis_part1 | -0.03085 | 0.02592 | -0.02418 | 0.02656 | 0.007 | 0.075 |
| Left.parstriangularis_part1 | 0.00651 | 0.0311 | 0.00744 | 0.03147 | 0.789 | 0.91 |
| Left.parstriangularis_part2 | 0.01746 | 0.02996 | 0.01672 | 0.02966 | 0.86 | 0.936 |
| Left.pericalcarine_part1 | -0.02086 | 0.02139 | -0.01997 | 0.02074 | 0.645 | 0.849 |
| Left.pericalcarine_part2 | -0.02751 | 0.01819 | -0.02419 | 0.01846 | 0.058 | 0.253 |
| Left.postcentral_part1 | 0.01344 | 0.02427 | 0.00602 | 0.02505 | 0.001 | 0.028 |
| Left.postcentral_part2 | 0.02003 | 0.02459 | 0.02653 | 0.02506 | 0.012 | 0.107 |
| Left.postcentral_part3 | 0.02621 | 0.02312 | 0.02002 | 0.0229 | 0.006 | 0.073 |
| Left.postcentral_part4 | -0.01606 | 0.03335 | -0.01035 | 0.03305 | 0.078 | 0.295 |
| Left.postcentral_part5 | 0.00858 | 0.02412 | 0.00051 | 0.02789 | 0.001 | 0.031 |
| Left.postcentral_part6 | 0.00257 | 0.02632 | 0.00538 | 0.02272 | 0.279 | 0.544 |
| Left.postcentral_part7 | 0.02605 | 0.0274 | 0.02476 | 0.02556 | 0.668 | 0.85 |
| Left.postcentral_part8 | 4.00E-04 | 0.02755 | 0.00319 | 0.02507 | 0.312 | 0.582 |
| Left.posteriorcingulate_part1 | 0.00865 | 0.03105 | 0.0108 | 0.02758 | 0.36 | 0.612 |
| Left.posteriorcingulate_part2 | -0.02878 | 0.02093 | -0.02899 | 0.02011 | 0.879 | 0.95 |
| Left.precentral_part1 | -0.01287 | 0.03104 | -0.01405 | 0.02873 | 0.782 | 0.91 |
| Left.precentral_part2 | 0.05385 | 0.02207 | 0.0506 | 0.02366 | 0.14 | 0.386 |
| Left.precentral_part3 | 0.01712 | 0.02981 | 0.00877 | 0.03169 | 0.006 | 0.073 |
| Left.precentral_part4 | 0.04119 | 0.02621 | 0.03874 | 0.02753 | 0.333 | 0.594 |
| Left.precentral_part5 | 0.03646 | 0.03034 | 0.02572 | 0.02856 | <0.001 | 0.015 |
| Left.precentral_part6 | 0.02775 | 0.03296 | 0.02259 | 0.03042 | 0.137 | 0.386 |
| Left.precentral_part7 | 0.04696 | 0.02973 | 0.04235 | 0.03029 | 0.152 | 0.386 |
| Left.precentral_part8 | 0.04266 | 0.02649 | 0.03309 | 0.02891 | <0.001 | 0.015 |
| Left.precentral_part9 | 0.02885 | 0.03215 | 0.02845 | 0.03082 | 0.969 | 0.982 |
| Left.precuneus_part1 | -0.01028 | 0.02932 | -0.00968 | 0.03057 | 0.817 | 0.915 |
| Left.precuneus_part2 | 0.03649 | 0.02617 | 0.03405 | 0.02361 | 0.331 | 0.594 |
| Left.precuneus_part3 | 0.02426 | 0.02724 | 0.02843 | 0.02525 | 0.087 | 0.312 |
| Left.precuneus_part4 | 0.01536 | 0.02943 | 0.01394 | 0.02776 | 0.761 | 0.905 |
| Left.precuneus_part5 | 0.02484 | 0.02489 | 0.0205 | 0.02531 | 0.086 | 0.312 |
| Left.precuneus_part6 | 0.0149 | 0.02621 | 0.01257 | 0.02638 | 0.382 | 0.636 |
| Left.precuneus_part7 | 0.01716 | 0.03054 | 0.01357 | 0.02982 | 0.263 | 0.531 |
| Left.rostralanteriorcingulate_part1 | -0.00153 | 0.02545 | 0.00081 | 0.02341 | 0.278 | 0.544 |
| Left.rostralmiddlefrontal_part1 | 0.005 | 0.03051 | 0.00698 | 0.02993 | 0.536 | 0.777 |
| Left.rostralmiddlefrontal_part10 | -0.00547 | 0.02856 | -0.00443 | 0.02521 | 0.948 | 0.977 |
| Left.rostralmiddlefrontal_part2 | -0.02646 | 0.03006 | -0.02211 | 0.03021 | 0.139 | 0.386 |
| Left.rostralmiddlefrontal_part3 | 0.02113 | 0.03105 | 0.01973 | 0.02823 | 0.636 | 0.844 |
| Left.rostralmiddlefrontal_part4 | -0.05373 | 0.0226 | -0.04684 | 0.02595 | 0.004 | 0.056 |
| Left.rostralmiddlefrontal_part5 | 0.01997 | 0.03035 | 0.02032 | 0.02787 | 0.921 | 0.964 |
| Left.rostralmiddlefrontal_part6 | -0.0416 | 0.02836 | -0.03556 | 0.02721 | 0.031 | 0.167 |
| Left.rostralmiddlefrontal_part7 | 0.01375 | 0.0296 | 0.01592 | 0.02883 | 0.435 | 0.682 |
| Left.rostralmiddlefrontal_part8 | -0.01267 | 0.02852 | -0.01016 | 0.02742 | 0.391 | 0.648 |
| Left.rostralmiddlefrontal_part9 | -0.01537 | 0.02984 | -0.01014 | 0.02968 | 0.107 | 0.362 |
| Left.superiorfrontal_part1 | -0.02333 | 0.02676 | -0.02023 | 0.02565 | 0.179 | 0.423 |
| Left.superiorfrontal_part10 | 0.04318 | 0.02612 | 0.0389 | 0.02519 | 0.13 | 0.386 |
| Left.superiorfrontal_part11 | 0.04483 | 0.02781 | 0.03998 | 0.02626 | 0.061 | 0.26 |
| Left.superiorfrontal_part12 | 0.04058 | 0.02718 | 0.03472 | 0.02628 | 0.032 | 0.169 |
| Left.superiorfrontal_part13 | 0.04431 | 0.02436 | 0.03876 | 0.02562 | 0.026 | 0.153 |
| Left.superiorfrontal_part2 | 0.04507 | 0.02832 | 0.03415 | 0.02803 | <0.001 | 0.008 |
| Left.superiorfrontal_part3 | 0.01499 | 0.03048 | 0.01624 | 0.03081 | 0.698 | 0.853 |
| Left.superiorfrontal_part4 | 0.0457 | 0.0273 | 0.04174 | 0.02801 | 0.127 | 0.386 |
| Left.superiorfrontal_part5 | 0.01863 | 0.02959 | 0.02047 | 0.02897 | 0.542 | 0.777 |
| Left.superiorfrontal_part6 | 0.04718 | 0.02482 | 0.03839 | 0.02445 | <0.001 | 0.015 |
| Left.superiorfrontal_part7 | 0.04229 | 0.02381 | 0.03634 | 0.0241 | 0.014 | 0.112 |
| Left.superiorfrontal_part8 | 0.03538 | 0.0296 | 0.02871 | 0.02829 | 0.029 | 0.166 |
| Left.superiorfrontal_part9 | 0.03811 | 0.02836 | 0.03619 | 0.02663 | 0.468 | 0.711 |
| Left.superiorparietal_part1 | 0.01865 | 0.02747 | 0.01206 | 0.02683 | 0.007 | 0.075 |
| Left.superiorparietal_part10 | 0.00871 | 0.03208 | 0.00095 | 0.03109 | 0.005 | 0.067 |
| Left.superiorparietal_part2 | -0.01571 | 0.03156 | -0.02001 | 0.03065 | 0.168 | 0.414 |
| Left.superiorparietal_part3 | 0.01806 | 0.02754 | 0.01273 | 0.02911 | 0.038 | 0.187 |
| Left.superiorparietal_part4 | 0.03298 | 0.02462 | 0.03487 | 0.02047 | 0.358 | 0.612 |
| Left.superiorparietal_part5 | 0.02031 | 0.0277 | 9.00E-05 | 0.02804 | <0.001 | <0.001 |
| Left.superiorparietal_part6 | 0.00801 | 0.03457 | 0.00366 | 0.03119 | 0.18 | 0.423 |
| Left.superiorparietal_part7 | 0.01977 | 0.03001 | 0.00739 | 0.03292 | <0.001 | 0.006 |
| Left.superiorparietal_part8 | 0.0404 | 0.02353 | 0.03914 | 0.02264 | 0.543 | 0.777 |
| Left.superiorparietal_part9 | 0.03988 | 0.02286 | 0.03412 | 0.02485 | 0.01 | 0.092 |
| Left.superiortemporal_part1 | 0.04426 | 0.02244 | 0.04587 | 0.01962 | 0.404 | 0.661 |
| Left.superiortemporal_part2 | 0.04105 | 0.02292 | 0.0389 | 0.02312 | 0.302 | 0.569 |
| Left.superiortemporal_part3 | -4.00E-05 | 0.02927 | -0.00073 | 0.02414 | 0.688 | 0.85 |
| Left.superiortemporal_part4 | 0.0304 | 0.02654 | 0.03427 | 0.02592 | 0.148 | 0.386 |
| Left.superiortemporal_part5 | 0.02421 | 0.02953 | 0.0259 | 0.02727 | 0.492 | 0.74 |
| Left.superiortemporal_part6 | 0.0378 | 0.02727 | 0.03668 | 0.02793 | 0.649 | 0.85 |
| Left.superiortemporal_part7 | 0.05091 | 0.02204 | 0.05166 | 0.0212 | 0.836 | 0.923 |
| Left.supramarginal_part1 | 0.02701 | 0.03103 | 0.02416 | 0.02529 | 0.283 | 0.544 |
| Left.supramarginal_part2 | 0.02072 | 0.02456 | 0.02387 | 0.02456 | 0.179 | 0.423 |
| Left.supramarginal_part3 | -0.00376 | 0.02878 | -0.01044 | 0.02815 | 0.013 | 0.109 |
| Left.supramarginal_part4 | 0.0192 | 0.02835 | 0.02283 | 0.02999 | 0.335 | 0.594 |
| Left.supramarginal_part5 | -0.00692 | 0.03111 | -0.00723 | 0.02734 | 0.77 | 0.907 |
| Left.supramarginal_part6 | 0.01952 | 0.03353 | 0.01638 | 0.0318 | 0.264 | 0.531 |
| Left.supramarginal_part7 | 0.0322 | 0.02799 | 0.03175 | 0.02526 | 0.759 | 0.905 |
| Left.temporalpole_part1 | 0.01109 | 0.02627 | 0.0122 | 0.02475 | 0.701 | 0.853 |
| Left.transversetemporal_part1 | 0.02511 | 0.02542 | 0.02496 | 0.02562 | 0.954 | 0.977 |
| Right.bankssts_part1 | 0.05197 | 0.02259 | 0.05314 | 0.02101 | 0.588 | 0.803 |
| Right.bankssts_part2 | 0.03883 | 0.02866 | 0.03916 | 0.03002 | 0.882 | 0.95 |
| Right.caudalanteriorcingulate_part1 | -0.01163 | 0.02125 | -0.00852 | 0.0233 | 0.121 | 0.386 |
| Right.caudalmiddlefrontal_part1 | 0.0388 | 0.02085 | 0.03735 | 0.02106 | 0.567 | 0.787 |
| Right.caudalmiddlefrontal_part2 | 0.04168 | 0.03013 | 0.03812 | 0.02851 | 0.188 | 0.433 |
| Right.caudalmiddlefrontal_part3 | 0.03472 | 0.02764 | 0.02908 | 0.02586 | 0.024 | 0.142 |
| Right.caudalmiddlefrontal_part4 | 0.04629 | 0.02573 | 0.039 | 0.02692 | 0.003 | 0.054 |
| Right.cuneus_part1 | 0.00052 | 0.02675 | -0.00033 | 0.02563 | 0.686 | 0.85 |
| Right.cuneus_part2 | -0.03795 | 0.02439 | -0.0371 | 0.02174 | 0.635 | 0.844 |
| Right.cuneus_part3 | -0.01295 | 0.02062 | -0.01029 | 0.01955 | 0.193 | 0.438 |
| Right.entorhinal_part1 | -0.04157 | 0.01674 | -0.04057 | 0.01645 | 0.689 | 0.85 |
| Right.frontalpole_part1 | -0.03971 | 0.02244 | -0.03757 | 0.01864 | 0.357 | 0.612 |
| Right.fusiform_part1 | -0.01079 | 0.02628 | -0.01024 | 0.02573 | 0.611 | 0.822 |
| Right.fusiform_part2 | -0.02146 | 0.02765 | -0.02215 | 0.02633 | 0.679 | 0.85 |
| Right.fusiform_part3 | 0.01298 | 0.0268 | 0.00414 | 0.02681 | 0.002 | 0.031 |
| Right.fusiform_part4 | -0.00299 | 0.03051 | -0.00651 | 0.02947 | 0.252 | 0.514 |
| Right.fusiform_part5 | 0.01747 | 0.02541 | 0.01638 | 0.02548 | 0.663 | 0.85 |
| Right.inferiorparietal_part1 | 0.02913 | 0.02472 | 0.02818 | 0.02654 | 0.687 | 0.85 |
| Right.inferiorparietal_part10 | -0.00726 | 0.03191 | -0.00192 | 0.03034 | 0.099 | 0.344 |
| Right.inferiorparietal_part2 | -0.02657 | 0.02247 | -0.02507 | 0.02547 | 0.531 | 0.777 |
| Right.inferiorparietal_part3 | 0.01214 | 0.03241 | 0.01396 | 0.03051 | 0.584 | 0.803 |
| Right.inferiorparietal_part4 | -0.00777 | 0.0298 | -0.01349 | 0.02406 | 0.026 | 0.153 |
| Right.inferiorparietal_part5 | 0.00648 | 0.03051 | 0.00516 | 0.02602 | 0.811 | 0.912 |
| Right.inferiorparietal_part6 | 0.03018 | 0.02748 | 0.02599 | 0.02992 | 0.139 | 0.386 |
| Right.inferiorparietal_part7 | -0.02291 | 0.02725 | -0.02219 | 0.02391 | 0.795 | 0.91 |
| Right.inferiorparietal_part8 | -0.01501 | 0.02489 | -0.01686 | 0.02812 | 0.36 | 0.612 |
| Right.inferiorparietal_part9 | -0.0014 | 0.03291 | -0.00692 | 0.03106 | 0.047 | 0.214 |
| Right.inferiortemporal_part1 | -0.0113 | 0.02487 | -0.01319 | 0.02563 | 0.314 | 0.583 |
| Right.inferiortemporal_part2 | -0.00524 | 0.02686 | -0.00929 | 0.02691 | 0.187 | 0.433 |
| Right.inferiortemporal_part3 | -0.02096 | 0.0283 | -0.02513 | 0.0277 | 0.113 | 0.375 |
| Right.inferiortemporal_part4 | -0.01552 | 0.02496 | -0.01475 | 0.02445 | 0.7 | 0.853 |
| Right.inferiortemporal_part5 | -0.03242 | 0.02764 | -0.03175 | 0.0247 | 0.798 | 0.91 |
| Right.insula_part1 | 0.0233 | 0.02153 | 0.02155 | 0.02128 | 0.434 | 0.682 |
| Right.insula_part2 | 0.03489 | 0.02518 | 0.03496 | 0.02279 | 0.894 | 0.957 |
| Right.insula_part3 | 0.02527 | 0.0266 | 0.02537 | 0.02314 | 0.898 | 0.957 |
| Right.insula_part4 | 0.03153 | 0.02149 | 0.03064 | 0.02142 | 0.628 | 0.841 |
| Right.isthmuscingulate_part1 | -0.01289 | 0.02525 | -0.0094 | 0.02591 | 0.165 | 0.412 |
| Right.isthmuscingulate_part2 | -0.03603 | 0.02133 | -0.03187 | 0.02052 | 0.023 | 0.142 |
| Right.lateraloccipital_part1 | -0.06652 | 0.01981 | -0.06449 | 0.02004 | 0.29 | 0.552 |
| Right.lateraloccipital_part2 | -0.01002 | 0.03133 | -0.01005 | 0.03223 | 0.771 | 0.907 |
| Right.lateraloccipital_part3 | -0.05824 | 0.02161 | -0.05311 | 0.02136 | 0.016 | 0.118 |
| Right.lateraloccipital_part4 | -0.02891 | 0.03065 | -0.0289 | 0.02827 | 0.932 | 0.964 |
| Right.lateraloccipital_part5 | -0.04218 | 0.01761 | -0.04004 | 0.02072 | 0.174 | 0.423 |
| Right.lateraloccipital_part6 | -0.02001 | 0.02932 | -0.02258 | 0.02423 | 0.334 | 0.594 |
| Right.lateraloccipital_part7 | -0.04669 | 0.02428 | -0.0452 | 0.02302 | 0.417 | 0.668 |
| Right.lateraloccipital_part8 | -0.05164 | 0.02171 | -0.05261 | 0.02219 | 0.69 | 0.85 |
| Right.lateraloccipital_part9 | -0.04065 | 0.02538 | -0.04087 | 0.02099 | 0.958 | 0.977 |
| Right.lateralorbitofrontal_part1 | -0.03208 | 0.0293 | -0.02814 | 0.02659 | 0.144 | 0.386 |
| Right.lateralorbitofrontal_part2 | -0.05707 | 0.02191 | -0.0527 | 0.02158 | 0.031 | 0.167 |
| Right.lateralorbitofrontal_part3 | 0.01022 | 0.03139 | 0.01323 | 0.03002 | 0.238 | 0.505 |
| Right.lateralorbitofrontal_part4 | -0.05529 | 0.02078 | -0.0512 | 0.02327 | 0.042 | 0.192 |
| Right.lingual_part1 | -0.01562 | 0.02612 | -0.0151 | 0.02505 | 0.825 | 0.915 |
| Right.lingual_part2 | -0.04189 | 0.02364 | -0.04023 | 0.02181 | 0.427 | 0.679 |
| Right.lingual_part3 | -0.00069 | 0.02983 | -0.00193 | 0.02783 | 0.709 | 0.86 |
| Right.lingual_part4 | -0.02468 | 0.01957 | -0.02085 | 0.01837 | 0.041 | 0.192 |
| Right.lingual_part5 | -0.01863 | 0.02075 | -0.01621 | 0.02043 | 0.243 | 0.513 |
| Right.lingual_part6 | -0.03003 | 0.02226 | -0.02593 | 0.02205 | 0.035 | 0.172 |
| Right.medialorbitofrontal_part1 | -0.03486 | 0.0292 | -0.02653 | 0.02666 | 0.002 | 0.041 |
| Right.medialorbitofrontal_part2 | -0.04253 | 0.02428 | -0.03673 | 0.02493 | 0.016 | 0.118 |
| Right.medialorbitofrontal_part3 | -0.03693 | 0.0247 | -0.03349 | 0.02611 | 0.133 | 0.386 |
| Right.middletemporal_part1 | 0.01697 | 0.02683 | 0.01944 | 0.02653 | 0.377 | 0.634 |
| Right.middletemporal_part2 | 0.00107 | 0.02551 | 0.00277 | 0.02205 | 0.534 | 0.777 |
| Right.middletemporal_part3 | 0.00268 | 0.02808 | 0.00219 | 0.02848 | 0.796 | 0.91 |
| Right.middletemporal_part4 | -0.00548 | 0.03072 | -0.00564 | 0.02941 | 0.93 | 0.964 |
| Right.middletemporal_part5 | -0.01906 | 0.024 | -0.01503 | 0.02348 | 0.082 | 0.306 |
| Right.middletemporal_part6 | 0.01169 | 0.02952 | 0.01161 | 0.02942 | 0.969 | 0.982 |
| Right.paracentral_part1 | 0.03795 | 0.02264 | 0.0349 | 0.02168 | 0.15 | 0.386 |
| Right.paracentral_part2 | 0.03285 | 0.03097 | 0.0206 | 0.03147 | <0.001 | 0.008 |
| Right.paracentral_part3 | 0.03724 | 0.02553 | 0.03187 | 0.02639 | 0.039 | 0.187 |
| Right.parahippocampal_part1 | 0.01559 | 0.02281 | 0.01298 | 0.02237 | 0.166 | 0.414 |
| Right.parahippocampal_part2 | 0.01559 | 0.02269 | 0.01335 | 0.02314 | 0.332 | 0.594 |
| Right.parsopercularis_part1 | 0.04431 | 0.02145 | 0.0427 | 0.02152 | 0.361 | 0.612 |
| Right.parsopercularis_part2 | 0.03555 | 0.02753 | 0.03011 | 0.02809 | 0.039 | 0.187 |
| Right.parsopercularis_part3 | 0.05246 | 0.02201 | 0.04846 | 0.02344 | 0.058 | 0.253 |
| Right.parsorbitalis_part1 | -0.02754 | 0.02531 | -0.02517 | 0.02388 | 0.229 | 0.493 |
| Right.parstriangularis_part1 | -0.02029 | 0.0312 | -0.01292 | 0.03294 | 0.014 | 0.112 |
| Right.parstriangularis_part2 | 0.03585 | 0.02673 | 0.03586 | 0.02466 | 0.902 | 0.957 |
| Right.parstriangularis_part3 | 0.00566 | 0.03048 | 0.01034 | 0.02776 | 0.111 | 0.37 |
| Right.pericalcarine_part1 | -0.02111 | 0.02046 | -0.01668 | 0.02062 | 0.03 | 0.167 |
| Right.pericalcarine_part2 | -0.02472 | 0.02242 | -0.02302 | 0.02151 | 0.455 | 0.7 |
| Right.pericalcarine_part3 | -0.01603 | 0.02091 | -0.01354 | 0.02044 | 0.232 | 0.495 |
| Right.postcentral_part1 | 0.00415 | 0.02683 | -0.002 | 0.02472 | 0.016 | 0.118 |
| Right.postcentral_part2 | 0.03156 | 0.02866 | 0.03468 | 0.02644 | 0.251 | 0.514 |
| Right.postcentral_part3 | 0.02725 | 0.02238 | 0.02334 | 0.0204 | 0.062 | 0.26 |
| Right.postcentral_part4 | -0.02332 | 0.02798 | -0.0182 | 0.02788 | 0.095 | 0.336 |
| Right.postcentral_part5 | 0.02848 | 0.02574 | 0.02063 | 0.02854 | 0.004 | 0.057 |
| Right.postcentral_part6 | 0.00216 | 0.02956 | -0.0012 | 0.02943 | 0.198 | 0.446 |
| Right.postcentral_part7 | 0.01331 | 0.02569 | 0.00879 | 0.02695 | 0.075 | 0.293 |
| Right.postcentral_part8 | 0.01621 | 0.02368 | 0.01515 | 0.02374 | 0.607 | 0.822 |
| Right.posteriorcingulate_part1 | -0.02113 | 0.03012 | -0.01797 | 0.03013 | 0.282 | 0.544 |
| Right.posteriorcingulate_part2 | -0.02694 | 0.0167 | -0.02321 | 0.01709 | 0.015 | 0.113 |
| Right.precentral_part1 | 0.05379 | 0.02395 | 0.05178 | 0.0234 | 0.423 | 0.676 |
| Right.precentral_part2 | 0.02534 | 0.02716 | 0.01647 | 0.02943 | 0.002 | 0.035 |
| Right.precentral_part3 | 0.04038 | 0.02859 | 0.04176 | 0.02751 | 0.675 | 0.85 |
| Right.precentral_part4 | 0.03473 | 0.02941 | 0.02677 | 0.02964 | 0.007 | 0.075 |
| Right.precentral_part5 | 0.03929 | 0.03296 | 0.03755 | 0.03039 | 0.539 | 0.777 |
| Right.precentral_part6 | -0.01043 | 0.03014 | -0.01268 | 0.0294 | 0.436 | 0.682 |
| Right.precentral_part7 | 0.04213 | 0.03056 | 0.03813 | 0.02644 | 0.126 | 0.386 |
| Right.precentral_part8 | 0.01454 | 0.02685 | 0.01426 | 0.02545 | 0.913 | 0.959 |
| Right.precentral_part9 | 0.0407 | 0.02768 | 0.03658 | 0.02821 | 0.148 | 0.386 |
| Right.precuneus_part1 | -0.01612 | 0.02943 | -0.01571 | 0.02984 | 0.953 | 0.977 |
| Right.precuneus_part2 | 0.02432 | 0.0289 | 0.0245 | 0.02802 | 0.812 | 0.912 |
| Right.precuneus_part3 | 0.03749 | 0.02409 | 0.03355 | 0.02397 | 0.076 | 0.293 |
| Right.precuneus_part4 | 0.0178 | 0.02692 | 0.01632 | 0.02401 | 0.641 | 0.848 |
| Right.precuneus_part5 | 0.01222 | 0.02593 | 0.01129 | 0.0249 | 0.685 | 0.85 |
| Right.precuneus_part6 | 0.02455 | 0.02855 | 0.01959 | 0.02761 | 0.073 | 0.292 |
| Right.precuneus_part7 | 0.00474 | 0.02447 | 0.01192 | 0.02614 | 0.005 | 0.061 |
| Right.rostralanteriorcingulate_part1 | -0.01128 | 0.03131 | -0.00488 | 0.03054 | 0.033 | 0.169 |
| Right.rostralmiddlefrontal_part1 | -0.05562 | 0.02257 | -0.05169 | 0.02386 | 0.083 | 0.306 |
| Right.rostralmiddlefrontal_part10 | -0.00723 | 0.0312 | 0.00172 | 0.02776 | 0.002 | 0.041 |
| Right.rostralmiddlefrontal_part2 | -0.03341 | 0.0295 | -0.02875 | 0.02737 | 0.125 | 0.386 |
| Right.rostralmiddlefrontal_part3 | 0.01949 | 0.03009 | 0.01767 | 0.02851 | 0.511 | 0.764 |
| Right.rostralmiddlefrontal_part4 | -0.04402 | 0.02649 | -0.03851 | 0.02645 | 0.033 | 0.169 |
| Right.rostralmiddlefrontal_part5 | 0.01827 | 0.02854 | 0.01645 | 0.02625 | 0.409 | 0.663 |
| Right.rostralmiddlefrontal_part6 | -0.02824 | 0.02699 | -0.02444 | 0.02428 | 0.151 | 0.386 |
| Right.rostralmiddlefrontal_part7 | 0.00098 | 0.03254 | 0.00658 | 0.02894 | 0.072 | 0.29 |
| Right.rostralmiddlefrontal_part8 | -0.00778 | 0.03202 | -0.00854 | 0.02888 | 0.841 | 0.925 |
| Right.rostralmiddlefrontal_part9 | -0.03332 | 0.02844 | -0.02928 | 0.02682 | 0.161 | 0.407 |
| Right.superiorfrontal_part1 | 0.04382 | 0.02333 | 0.04002 | 0.02425 | 0.121 | 0.386 |
| Right.superiorfrontal_part10 | 0.03599 | 0.02548 | 0.03552 | 0.02284 | 0.823 | 0.915 |
| Right.superiorfrontal_part11 | 0.03423 | 0.02627 | 0.03134 | 0.02708 | 0.249 | 0.514 |
| Right.superiorfrontal_part12 | 0.03403 | 0.02848 | 0.03383 | 0.0302 | 0.933 | 0.964 |
| Right.superiorfrontal_part13 | 0.04277 | 0.02333 | 0.04091 | 0.02555 | 0.454 | 0.7 |
| Right.superiorfrontal_part2 | -0.02569 | 0.02686 | -0.02278 | 0.0263 | 0.282 | 0.544 |
| Right.superiorfrontal_part3 | 0.03301 | 0.02935 | 0.02926 | 0.02818 | 0.212 | 0.467 |
| Right.superiorfrontal_part4 | 0.00214 | 0.03257 | 0.00294 | 0.03172 | 0.717 | 0.865 |
| Right.superiorfrontal_part5 | 0.04293 | 0.02898 | 0.03896 | 0.02794 | 0.152 | 0.386 |
| Right.superiorfrontal_part6 | 0.00744 | 0.03051 | 0.01011 | 0.03122 | 0.353 | 0.612 |
| Right.superiorfrontal_part7 | 0.03737 | 0.02874 | 0.03075 | 0.02657 | 0.023 | 0.142 |
| Right.superiorfrontal_part8 | 0.0253 | 0.03142 | 0.02597 | 0.02899 | 0.811 | 0.912 |
| Right.superiorfrontal_part9 | 0.03909 | 0.02458 | 0.03529 | 0.02694 | 0.144 | 0.386 |
| Right.superiorparietal_part1 | 0.01011 | 0.02849 | 0.00576 | 0.02858 | 0.143 | 0.386 |
| Right.superiorparietal_part10 | 0.02851 | 0.02571 | 0.02243 | 0.03071 | 0.022 | 0.142 |
| Right.superiorparietal_part2 | 0.0248 | 0.02659 | 0.01803 | 0.02714 | 0.007 | 0.075 |
| Right.superiorparietal_part3 | -0.0171 | 0.0344 | -0.01764 | 0.03155 | 0.826 | 0.915 |
| Right.superiorparietal_part4 | 0.01488 | 0.03055 | 0.00846 | 0.02856 | 0.02 | 0.137 |
| Right.superiorparietal_part5 | 0.03628 | 0.024 | 0.03341 | 0.02295 | 0.182 | 0.424 |
| Right.superiorparietal_part6 | 0.02615 | 0.02872 | 0.02153 | 0.0295 | 0.076 | 0.293 |
| Right.superiorparietal_part7 | 0.0158 | 0.03209 | 0.00778 | 0.03177 | 0.008 | 0.076 |
| Right.superiorparietal_part8 | 0.02983 | 0.02385 | 0.03071 | 0.02234 | 0.608 | 0.822 |
| Right.superiorparietal_part9 | 0.02348 | 0.02923 | 0.01237 | 0.03255 | <0.001 | 0.008 |
| Right.superiortemporal_part1 | 0.03016 | 0.02588 | 0.03292 | 0.02156 | 0.252 | 0.514 |
| Right.superiortemporal_part2 | 0.01767 | 0.02771 | 0.02045 | 0.02413 | 0.338 | 0.595 |
| Right.superiortemporal_part3 | 0.02755 | 0.0264 | 0.03068 | 0.02315 | 0.206 | 0.456 |
| Right.superiortemporal_part4 | 0.03756 | 0.02659 | 0.03971 | 0.02363 | 0.463 | 0.71 |
| Right.superiortemporal_part5 | 0.02821 | 0.02718 | 0.02634 | 0.0282 | 0.536 | 0.777 |
| Right.superiortemporal_part6 | 0.049 | 0.01995 | 0.04676 | 0.01827 | 0.224 | 0.49 |
| Right.supramarginal_part1 | 0.03992 | 0.02331 | 0.03542 | 0.02582 | 0.05 | 0.223 |
| Right.supramarginal_part2 | 0.02123 | 0.02497 | 0.02097 | 0.02627 | 0.871 | 0.944 |
| Right.supramarginal_part3 | 0.01761 | 0.03109 | 0.01195 | 0.03103 | 0.022 | 0.142 |
| Right.supramarginal_part4 | 0.03147 | 0.02705 | 0.03072 | 0.02517 | 0.652 | 0.85 |
| Right.supramarginal_part5 | -0.00179 | 0.02965 | -0.00073 | 0.03066 | 0.985 | 0.992 |
| Right.supramarginal_part6 | 0.00776 | 0.03118 | 0.0063 | 0.03039 | 0.396 | 0.653 |
| Right.supramarginal_part7 | 0.04438 | 0.02712 | 0.0425 | 0.03122 | 0.412 | 0.664 |
| Right.temporalpole_part1 | 0.00316 | 0.0295 | 0.00206 | 0.02833 | 0.555 | 0.779 |
| Right.transversetemporal_part1 | 0.02264 | 0.02217 | 0.02329 | 0.02204 | 0.791 | 0.91 |

FDR: false discovery rate, SD: standard deviation

# **Supplementary Table 3. Group comparisons of the mean morphometric similarity of subnetworks**

|  |  | Healthy control | | Schizophrenia | |  |  |
| --- | --- | --- | --- | --- | --- | --- | --- |
| Atlas | Subnetwork | Mean | SD | Mean | SD | Unadjusted p | FDR adjusted p |
| Yeo | Visual | 0.2699 | 0.06271 | 0.268 | 0.0655 | 0.884 | 0.884 |
|  | Somatosensory | 0.2459 | 0.06716 | 0.22206 | 0.07225 | <0.001 | 0.002 |
|  | Dorsal attention | 0.10903 | 0.05495 | 0.08825 | 0.05592 | <0.001 | 0.001 |
|  | Salience/Ventral attention | 0.13825 | 0.05577 | 0.14162 | 0.05271 | 0.557 | 0.709 |
|  | Limbic | 0.2336 | 0.0712 | 0.21436 | 0.0716 | 0.003 | 0.009 |
|  | Fronto-parietal | 0.03476 | 0.02852 | 0.03442 | 0.03454 | 0.746 | 0.870 |
|  | Default mode | 0.08919 | 0.0324 | 0.09107 | 0.03515 | 0.465 | 0.709 |
| Von economo | Primary motor cortex | 0.45136 | 0.13241 | 0.40709 | 0.13386 | 0.001 | 0.004 |
|  | Association cortex 1 | 0.06629 | 0.02211 | 0.05812 | 0.02343 | <0.001 | 0.002 |
|  | Association cortex 2 | 0.05231 | 0.02686 | 0.05224 | 0.02815 | 0.851 | 0.884 |
|  | Primary/secondary cortex | 0.31978 | 0.06656 | 0.30886 | 0.0765 | 0.122 | 0.219 |
|  | Primary sensory cortex | 0.31773 | 0.08245 | 0.31443 | 0.08175 | 0.544 | 0.709 |
|  | Limbic regions | 0.24218 | 0.08885 | 0.22917 | 0.08482 | 0.125 | 0.219 |
|  | Insular cortex | 0.72888 | 0.20539 | 0.68871 | 0.2125 | 0.038 | 0.088 |

FDR: false discovery rate, SD: standard deviation

# **Supplementary Table 4. Group comparisons of nodal measures of the morphometric similarity network**

|  |  | Healthy control | | Schizophrenia | |  |  |
| --- | --- | --- | --- | --- | --- | --- | --- |
| Measure | Structure | Mean | SD | Mean | SD | t | FDR adjusted p |
| Degree | Left.bankssts_part1 | 83.351 | 14.536 | 85.092 | 13.941 | 1.269 | 0.584 |
| Degree | Left.bankssts_part2 | 87.738 | 13.347 | 88.627 | 13.081 | 0.955 | 0.674 |
| Degree | Left.caudalanteriorcingulate_part1 | 63.882 | 12.476 | 65.811 | 13.283 | 1.69 | 0.454 |
| Degree | Left.caudalmiddlefrontal_part1 | 85.151 | 12.806 | 84.783 | 11.559 | -0.211 | 0.93 |
| Degree | Left.caudalmiddlefrontal_part2 | 85.611 | 12.768 | 82.815 | 13.455 | -2.066 | 0.34 |
| Degree | Left.caudalmiddlefrontal_part3 | 77.692 | 11.841 | 79.299 | 10.657 | 1.55 | 0.493 |
| Degree | Left.caudalmiddlefrontal_part4 | 84.924 | 13.935 | 84.605 | 13.672 | -0.216 | 0.927 |
| Degree | Left.cuneus_part1 | 76.633 | 6.332 | 78.195 | 6.301 | 2.504 | 0.219 |
| Degree | Left.cuneus_part2 | 79.693 | 6.035 | 80.197 | 5.758 | 1.055 | 0.653 |
| Degree | Left.entorhinal_part1 | 44.069 | 8.519 | 45.804 | 8.828 | 1.878 | 0.406 |
| Degree | Left.fusiform_part1 | 67.379 | 6.223 | 68.167 | 6.349 | 1.283 | 0.575 |
| Degree | Left.fusiform_part2 | 76.971 | 11.501 | 78.568 | 11.522 | 1.458 | 0.522 |
| Degree | Left.fusiform_part3 | 70.978 | 8.709 | 71.472 | 9.087 | 0.709 | 0.757 |
| Degree | Left.fusiform_part4 | 70.499 | 9.754 | 72.377 | 9.357 | 1.98 | 0.36 |
| Degree | Left.fusiform_part5 | 72.867 | 9.289 | 73.605 | 9.321 | 0.897 | 0.696 |
| Degree | Left.inferiorparietal_part1 | 74.367 | 10.188 | 73.912 | 10.447 | -0.466 | 0.848 |
| Degree | Left.inferiorparietal_part2 | 72.824 | 10.323 | 73.372 | 10.538 | 0.575 | 0.812 |
| Degree | Left.inferiorparietal_part3 | 71.05 | 9.016 | 69.869 | 9.477 | -1.309 | 0.567 |
| Degree | Left.inferiorparietal_part4 | 77.726 | 12.361 | 78.09 | 13.039 | 0.278 | 0.914 |
| Degree | Left.inferiorparietal_part5 | 70.347 | 10.868 | 69.272 | 10.196 | -1.248 | 0.59 |
| Degree | Left.inferiorparietal_part6 | 75.792 | 8.115 | 75.363 | 8.437 | -0.472 | 0.847 |
| Degree | Left.inferiorparietal_part7 | 71.192 | 9.729 | 72.147 | 8.976 | 1.032 | 0.657 |
| Degree | Left.inferiorparietal_part8 | 72.738 | 9.792 | 72.144 | 9.707 | -0.61 | 0.8 |
| Degree | Left.inferiortemporal_part1 | 75.159 | 11.624 | 75.249 | 11.221 | 0.163 | 0.941 |
| Degree | Left.inferiortemporal_part2 | 69.904 | 9.378 | 71.622 | 10.69 | 1.606 | 0.469 |
| Degree | Left.inferiortemporal_part3 | 74.512 | 10.365 | 72.516 | 10.879 | -1.864 | 0.407 |
| Degree | Left.inferiortemporal_part4 | 68.597 | 10.119 | 68.851 | 9.134 | 0.421 | 0.86 |
| Degree | Left.inferiortemporal_part5 | 73.262 | 11.62 | 72.074 | 10.411 | -1.212 | 0.593 |
| Degree | Left.inferiortemporal_part6 | 68.581 | 9.48 | 68.601 | 9.541 | -0.022 | 0.99 |
| Degree | Left.isthmuscingulate_part1 | 68.113 | 9.994 | 68.374 | 9.157 | 0.389 | 0.876 |
| Degree | Left.isthmuscingulate_part2 | 73.721 | 13.767 | 75.287 | 15.468 | 1.105 | 0.634 |
| Degree | Left.lateraloccipital_part1 | 77.129 | 11.754 | 78.534 | 10.877 | 1.235 | 0.591 |
| Degree | Left.lateraloccipital_part2 | 70.001 | 14.449 | 68.103 | 14.535 | -1.465 | 0.522 |
| Degree | Left.lateraloccipital_part3 | 83.957 | 13.141 | 85.125 | 13.676 | 0.948 | 0.674 |
| Degree | Left.lateraloccipital_part4 | 68.218 | 9.69 | 70.005 | 10.911 | 1.883 | 0.406 |
| Degree | Left.lateraloccipital_part5 | 58.98 | 10.005 | 60.822 | 10.177 | 1.88 | 0.406 |
| Degree | Left.lateraloccipital_part6 | 76.323 | 8.072 | 75.632 | 8.806 | -0.898 | 0.696 |
| Degree | Left.lateraloccipital_part7 | 73.527 | 8.809 | 72.522 | 9.506 | -0.987 | 0.673 |
| Degree | Left.lateraloccipital_part8 | 79.177 | 6.263 | 79.886 | 5.281 | 1.281 | 0.575 |
| Degree | Left.lateraloccipital_part9 | 72.224 | 8.961 | 72.51 | 9.009 | 0.468 | 0.847 |
| Degree | Left.lateralorbitofrontal_part1 | 76.685 | 6.082 | 77.411 | 6.094 | 1.221 | 0.591 |
| Degree | Left.lateralorbitofrontal_part2 | 72.434 | 10.061 | 72.831 | 8.904 | 0.609 | 0.8 |
| Degree | Left.lateralorbitofrontal_part3 | 75.842 | 7.039 | 75.966 | 7.025 | 0.145 | 0.942 |
| Degree | Left.lateralorbitofrontal_part4 | 75.34 | 7.31 | 76.095 | 6.615 | 1.263 | 0.589 |
| Degree | Left.lingual_part1 | 73.488 | 8.825 | 73.464 | 8.514 | -0.005 | 0.999 |
| Degree | Left.lingual_part2 | 73.925 | 8.049 | 74.159 | 8.391 | 0.394 | 0.875 |
| Degree | Left.lingual_part3 | 68.791 | 8.5 | 70.125 | 8.525 | 1.825 | 0.421 |
| Degree | Left.lingual_part4 | 73.084 | 8.323 | 72.353 | 9.19 | -0.837 | 0.705 |
| Degree | Left.lingual_part5 | 72.462 | 10.679 | 73.013 | 11.051 | 0.484 | 0.841 |
| Degree | Left.lingual_part6 | 72.136 | 9.698 | 72.808 | 9.166 | 0.65 | 0.786 |
| Degree | Left.medialorbitofrontal_part1 | 76.169 | 6.215 | 76.661 | 5.508 | 1.087 | 0.644 |
| Degree | Left.medialorbitofrontal_part2 | 71.899 | 10.214 | 70.768 | 9.56 | -1.237 | 0.591 |
| Degree | Left.medialorbitofrontal_part3 | 76.588 | 5.316 | 76.944 | 4.801 | 0.641 | 0.786 |
| Degree | Left.middletemporal_part1 | 70.219 | 8.193 | 71.091 | 7.094 | 1.099 | 0.637 |
| Degree | Left.middletemporal_part2 | 75.834 | 6.983 | 75.58 | 6.525 | -0.383 | 0.878 |
| Degree | Left.middletemporal_part3 | 59.088 | 12.227 | 61.779 | 12.797 | 2.021 | 0.348 |
| Degree | Left.middletemporal_part4 | 71.261 | 9.626 | 71.305 | 10.598 | 0.128 | 0.949 |
| Degree | Left.middletemporal_part5 | 65.663 | 12.016 | 67.851 | 11.568 | 1.818 | 0.423 |
| Degree | Left.parahippocampal_part1 | 76.142 | 7.672 | 76.44 | 8.796 | 0.263 | 0.915 |
| Degree | Left.parahippocampal_part2 | 74.981 | 7.354 | 74.867 | 7.795 | -0.094 | 0.963 |
| Degree | Left.paracentral_part1 | 77.269 | 11.569 | 78.458 | 10.93 | 1.03 | 0.657 |
| Degree | Left.paracentral_part2 | 75.832 | 6.906 | 75.918 | 7.83 | 0.196 | 0.934 |
| Degree | Left.paracentral_part3 | 79.674 | 10.286 | 78.833 | 11.142 | -0.846 | 0.705 |
| Degree | Left.parsopercularis_part1 | 87.961 | 10.064 | 85.339 | 11.189 | -2.496 | 0.219 |
| Degree | Left.parsopercularis_part2 | 91.837 | 9.495 | 88.765 | 12.716 | -2.757 | 0.188 |
| Degree | Left.parsopercularis_part3 | 88.806 | 12.33 | 83.459 | 14.196 | -4.05 | 0.013 |
| Degree | Left.parsorbitalis_part1 | 84.936 | 10.757 | 85.513 | 11.648 | 0.639 | 0.786 |
| Degree | Left.parstriangularis_part1 | 85.04 | 10.127 | 86.138 | 10.049 | 1.002 | 0.671 |
| Degree | Left.parstriangularis_part2 | 78.411 | 12.556 | 78.103 | 12.941 | -0.334 | 0.893 |
| Degree | Left.pericalcarine_part1 | 84.892 | 11.75 | 83.854 | 12.774 | -0.796 | 0.722 |
| Degree | Left.pericalcarine_part2 | 92.302 | 11.325 | 90.541 | 11.152 | -1.493 | 0.517 |
| Degree | Left.postcentral_part1 | 76.05 | 6.701 | 74.335 | 7.67 | -2.55 | 0.212 |
| Degree | Left.postcentral_part2 | 75.462 | 8.176 | 76.648 | 8.744 | 1.344 | 0.557 |
| Degree | Left.postcentral_part3 | 76.095 | 9.598 | 77.259 | 10.891 | 1.34 | 0.559 |
| Degree | Left.postcentral_part4 | 75.519 | 6.358 | 75.764 | 5.79 | 0.48 | 0.843 |
| Degree | Left.postcentral_part5 | 75.188 | 5.942 | 76.316 | 5.447 | 1.815 | 0.423 |
| Degree | Left.postcentral_part6 | 75.058 | 8.176 | 71.928 | 8.692 | -3.859 | 0.02 |
| Degree | Left.postcentral_part7 | 70.197 | 9.464 | 72.667 | 10.665 | 2.347 | 0.259 |
| Degree | Left.postcentral_part8 | 79.625 | 9.845 | 78.359 | 9.305 | -1.283 | 0.575 |
| Degree | Left.posteriorcingulate_part1 | 69.34 | 10.551 | 70.06 | 10.756 | 0.755 | 0.736 |
| Degree | Left.posteriorcingulate_part2 | 71.556 | 11.995 | 70.196 | 12.653 | -1.205 | 0.596 |
| Degree | Left.precentral_part1 | 72.493 | 9.776 | 72.992 | 9.149 | 0.425 | 0.859 |
| Degree | Left.precentral_part2 | 80.069 | 11.266 | 81.433 | 11.213 | 1.323 | 0.567 |
| Degree | Left.precentral_part3 | 71.431 | 9.821 | 72.252 | 11.011 | 0.741 | 0.74 |
| Degree | Left.precentral_part4 | 74.808 | 11.036 | 75.626 | 10.936 | 0.73 | 0.744 |
| Degree | Left.precentral_part5 | 56.05 | 9.067 | 56.09 | 8.787 | 0.149 | 0.941 |
| Degree | Left.precentral_part6 | 63.222 | 14.472 | 64.649 | 14.81 | 1.189 | 0.603 |
| Degree | Left.precentral_part7 | 88.517 | 10.873 | 87.066 | 11.845 | -1.381 | 0.541 |
| Degree | Left.precentral_part8 | 82.933 | 14.666 | 77.767 | 16.329 | -3.329 | 0.078 |
| Degree | Left.precentral_part9 | 85.155 | 11.605 | 86.107 | 12.42 | 0.729 | 0.744 |
| Degree | Left.precuneus_part1 | 89.033 | 14.388 | 85.054 | 14.633 | -2.621 | 0.191 |
| Degree | Left.precuneus_part2 | 85.226 | 17.091 | 83.643 | 17.532 | -0.694 | 0.762 |
| Degree | Left.precuneus_part3 | 90.805 | 13.211 | 89.718 | 13.522 | -0.677 | 0.772 |
| Degree | Left.precuneus_part4 | 90.021 | 13.074 | 86.934 | 12.863 | -2.471 | 0.223 |
| Degree | Left.precuneus_part5 | 82.794 | 16.833 | 84.03 | 17.138 | 0.966 | 0.673 |
| Degree | Left.precuneus_part6 | 69.629 | 10.998 | 68.524 | 11.097 | -1.104 | 0.634 |
| Degree | Left.precuneus_part7 | 82.725 | 11.54 | 82.143 | 12.085 | -0.539 | 0.824 |
| Degree | Left.rostralanteriorcingulate_part1 | 76.274 | 11.144 | 78.901 | 11.757 | 2.387 | 0.252 |
| Degree | Left.rostralmiddlefrontal_part1 | 76.867 | 8.809 | 76.151 | 10.688 | -0.861 | 0.701 |
| Degree | Left.rostralmiddlefrontal_part2 | 74.643 | 10.252 | 75.877 | 8.791 | 1.256 | 0.589 |
| Degree | Left.rostralmiddlefrontal_part3 | 74.663 | 10.089 | 76.335 | 9.114 | 1.765 | 0.425 |
| Degree | Left.rostralmiddlefrontal_part4 | 75.536 | 12.217 | 77.22 | 10.961 | 1.432 | 0.525 |
| Degree | Left.rostralmiddlefrontal_part5 | 73.958 | 8.885 | 74.188 | 8.711 | 0.265 | 0.915 |
| Degree | Left.rostralmiddlefrontal_part6 | 72.458 | 9.29 | 74.306 | 8.274 | 2.019 | 0.348 |
| Degree | Left.rostralmiddlefrontal_part7 | 74.383 | 9.012 | 75.177 | 8.294 | 0.773 | 0.73 |
| Degree | Left.rostralmiddlefrontal_part8 | 74.992 | 9.895 | 73.945 | 9.921 | -1.064 | 0.651 |
| Degree | Left.rostralmiddlefrontal_part9 | 75.728 | 12.263 | 76.619 | 12.026 | 0.827 | 0.709 |
| Degree | Left.rostralmiddlefrontal_part10 | 74.564 | 8.888 | 74.227 | 8.607 | -0.382 | 0.878 |
| Degree | Left.superiorfrontal_part1 | 75.348 | 11.54 | 74.923 | 11.847 | -0.462 | 0.848 |
| Degree | Left.superiorfrontal_part2 | 78.64 | 8.332 | 78.885 | 8.079 | 0.423 | 0.859 |
| Degree | Left.superiorfrontal_part3 | 75.31 | 11.458 | 76.258 | 12.519 | 0.775 | 0.73 |
| Degree | Left.superiorfrontal_part4 | 74.621 | 7.81 | 75.792 | 7.618 | 1.623 | 0.469 |
| Degree | Left.superiorfrontal_part5 | 73.892 | 9.119 | 75.39 | 8.606 | 1.935 | 0.38 |
| Degree | Left.superiorfrontal_part6 | 75.202 | 10.132 | 75.102 | 8.909 | -0.239 | 0.92 |
| Degree | Left.superiorfrontal_part7 | 87.738 | 13.09 | 88.212 | 12.403 | 0.507 | 0.83 |
| Degree | Left.superiorfrontal_part8 | 87.346 | 13.412 | 87.992 | 12.143 | 0.535 | 0.824 |
| Degree | Left.superiorfrontal_part9 | 87.395 | 14.18 | 85.999 | 12.992 | -1.031 | 0.657 |
| Degree | Left.superiorfrontal_part10 | 85.434 | 11.83 | 84.901 | 13.253 | -0.364 | 0.88 |
| Degree | Left.superiorfrontal_part11 | 89.449 | 12.827 | 84.702 | 14.215 | -3.582 | 0.043 |
| Degree | Left.superiorfrontal_part12 | 72.29 | 12.94 | 74.435 | 11.584 | 1.643 | 0.457 |
| Degree | Left.superiorfrontal_part13 | 91.181 | 13.828 | 89.863 | 12.972 | -1.055 | 0.653 |
| Degree | Left.superiorparietal_part1 | 71.502 | 13.991 | 72.742 | 14.304 | 0.915 | 0.69 |
| Degree | Left.superiorparietal_part2 | 90.356 | 11.173 | 88.436 | 13.746 | -1.405 | 0.536 |
| Degree | Left.superiorparietal_part3 | 86.321 | 11.345 | 84.692 | 12.287 | -1.249 | 0.59 |
| Degree | Left.superiorparietal_part4 | 85.026 | 13.914 | 83.94 | 13.867 | -0.666 | 0.777 |
| Degree | Left.superiorparietal_part5 | 83.311 | 15.02 | 82.835 | 13.624 | -0.285 | 0.912 |
| Degree | Left.superiorparietal_part6 | 78.47 | 9.99 | 76.575 | 10.074 | -2.13 | 0.324 |
| Degree | Left.superiorparietal_part7 | 71.268 | 10.877 | 71.195 | 10.553 | -0.189 | 0.935 |
| Degree | Left.superiorparietal_part8 | 73.572 | 9.084 | 74.321 | 9.036 | 0.931 | 0.682 |
| Degree | Left.superiorparietal_part9 | 77.157 | 10.381 | 76.398 | 9.512 | -0.892 | 0.696 |
| Degree | Left.superiorparietal_part10 | 80.409 | 9.475 | 80.453 | 9.1 | 0.027 | 0.987 |
| Degree | Left.superiortemporal_part1 | 70.164 | 13.587 | 61.945 | 12.809 | -6.599 | <0.001 |
| Degree | Left.superiortemporal_part2 | 74.446 | 10.742 | 74.71 | 9.718 | 0.217 | 0.927 |
| Degree | Left.superiortemporal_part3 | 75.073 | 11.439 | 73.615 | 10.621 | -1.544 | 0.494 |
| Degree | Left.superiortemporal_part4 | 82.13 | 10.421 | 81.971 | 10.656 | -0.324 | 0.898 |
| Degree | Left.superiortemporal_part5 | 83.634 | 11.081 | 81.53 | 9.721 | -2.125 | 0.324 |
| Degree | Left.superiortemporal_part6 | 90.917 | 11.407 | 91.209 | 10.952 | 0.522 | 0.827 |
| Degree | Left.superiortemporal_part7 | 88.838 | 8.757 | 89.567 | 8.923 | 0.913 | 0.691 |
| Degree | Left.supramarginal_part1 | 74.248 | 8.909 | 72.153 | 8.394 | -2.611 | 0.192 |
| Degree | Left.supramarginal_part2 | 86.134 | 10.372 | 87.673 | 10.598 | 1.56 | 0.485 |
| Degree | Left.supramarginal_part3 | 86.135 | 12.711 | 86.54 | 11.879 | 0.48 | 0.843 |
| Degree | Left.supramarginal_part4 | 86.142 | 12.874 | 86.287 | 13.191 | 0.162 | 0.941 |
| Degree | Left.supramarginal_part5 | 96.279 | 9.912 | 97.813 | 8.274 | 1.663 | 0.457 |
| Degree | Left.supramarginal_part6 | 77.15 | 10.499 | 76.872 | 8.756 | -0.305 | 0.906 |
| Degree | Left.supramarginal_part7 | 71.988 | 10.122 | 73.395 | 10.515 | 1.359 | 0.55 |
| Degree | Left.frontalpole_part1 | 71.951 | 10.002 | 73.041 | 8.515 | 1.134 | 0.63 |
| Degree | Left.temporalpole_part1 | 72.765 | 10.813 | 75.478 | 11.359 | 2.389 | 0.252 |
| Degree | Left.transversetemporal_part1 | 73.265 | 10.335 | 73.179 | 10.265 | -0.125 | 0.95 |
| Degree | Left.insula_part1 | 77.427 | 11.237 | 75.637 | 12.059 | -1.798 | 0.424 |
| Degree | Left.insula_part2 | 79.376 | 15.221 | 80.811 | 13.863 | 1.029 | 0.657 |
| Degree | Left.insula_part3 | 72.386 | 11.247 | 73.605 | 10.475 | 1.089 | 0.644 |
| Degree | Left.insula_part4 | 80.56 | 9.311 | 81.244 | 10.244 | 0.72 | 0.75 |
| Degree | Right.bankssts_part1 | 88.552 | 12.204 | 89.083 | 12.371 | 0.456 | 0.848 |
| Degree | Right.bankssts_part2 | 84.913 | 14.744 | 85.703 | 14.99 | 0.493 | 0.837 |
| Degree | Right.caudalanteriorcingulate_part1 | 64.42 | 10.259 | 66.741 | 10.416 | 2.433 | 0.233 |
| Degree | Right.caudalmiddlefrontal_part1 | 77.865 | 12.38 | 79.223 | 11.474 | 1.392 | 0.538 |
| Degree | Right.caudalmiddlefrontal_part2 | 86.419 | 15.029 | 84.217 | 14.656 | -1.594 | 0.471 |
| Degree | Right.caudalmiddlefrontal_part3 | 80.22 | 14.398 | 80.303 | 15.198 | 0.013 | 0.995 |
| Degree | Right.caudalmiddlefrontal_part4 | 89.133 | 13.215 | 87.094 | 12.257 | -1.743 | 0.429 |
| Degree | Right.cuneus_part1 | 76.685 | 6.741 | 76.268 | 6.604 | -0.764 | 0.732 |
| Degree | Right.cuneus_part2 | 76.86 | 5.534 | 76.466 | 5.034 | -0.763 | 0.732 |
| Degree | Right.cuneus_part3 | 77.006 | 5.933 | 77.44 | 5.204 | 0.773 | 0.73 |
| Degree | Right.entorhinal_part1 | 47.224 | 7.937 | 48.825 | 7.976 | 1.772 | 0.424 |
| Degree | Right.fusiform_part1 | 68.271 | 6.275 | 68.553 | 6.427 | 0.467 | 0.847 |
| Degree | Right.fusiform_part2 | 71.626 | 9.356 | 72.323 | 8.146 | 0.75 | 0.736 |
| Degree | Right.fusiform_part3 | 69.862 | 9.989 | 69.16 | 9.82 | -0.983 | 0.673 |
| Degree | Right.fusiform_part4 | 74.944 | 10.656 | 73.919 | 10.12 | -0.987 | 0.673 |
| Degree | Right.fusiform_part5 | 73.722 | 10.636 | 73.802 | 10.42 | 0.051 | 0.983 |
| Degree | Right.inferiorparietal_part1 | 75.892 | 12.787 | 77.166 | 11.506 | 0.998 | 0.673 |
| Degree | Right.inferiorparietal_part2 | 78.257 | 12.237 | 77.809 | 11.695 | -0.441 | 0.852 |
| Degree | Right.inferiorparietal_part3 | 73.325 | 11.088 | 75.287 | 10.006 | 1.806 | 0.424 |
| Degree | Right.inferiorparietal_part4 | 68.548 | 8.732 | 68.805 | 8.397 | 0.256 | 0.918 |
| Degree | Right.inferiorparietal_part5 | 77.797 | 11.517 | 77.945 | 12.146 | 0.158 | 0.941 |
| Degree | Right.inferiorparietal_part6 | 68.736 | 10.113 | 68.054 | 8.731 | -0.818 | 0.712 |
| Degree | Right.inferiorparietal_part7 | 73.442 | 9.881 | 72.822 | 9.843 | -0.606 | 0.8 |
| Degree | Right.inferiorparietal_part8 | 77.281 | 11.139 | 78.105 | 11.162 | 0.668 | 0.776 |
| Degree | Right.inferiorparietal_part9 | 70.857 | 9.667 | 71.679 | 9.831 | 0.87 | 0.697 |
| Degree | Right.inferiorparietal_part10 | 71.755 | 8.889 | 72.973 | 8.335 | 1.481 | 0.521 |
| Degree | Right.inferiortemporal_part1 | 69.214 | 10.853 | 70.193 | 11.092 | 0.764 | 0.732 |
| Degree | Right.inferiortemporal_part2 | 72.799 | 10.635 | 72.596 | 9.657 | -0.424 | 0.859 |
| Degree | Right.inferiortemporal_part3 | 75.693 | 8.252 | 74.509 | 8.344 | -1.4 | 0.537 |
| Degree | Right.inferiortemporal_part4 | 71.404 | 9.683 | 71.814 | 9.755 | 0.391 | 0.876 |
| Degree | Right.inferiortemporal_part5 | 72.021 | 8.934 | 71.708 | 9.268 | -0.299 | 0.907 |
| Degree | Right.isthmuscingulate_part1 | 69.087 | 7.453 | 70.18 | 8.016 | 1.403 | 0.536 |
| Degree | Right.isthmuscingulate_part2 | 82.982 | 9.648 | 84.753 | 9.039 | 2.022 | 0.348 |
| Degree | Right.lateraloccipital_part1 | 80.285 | 14.103 | 79.413 | 14.463 | -0.75 | 0.736 |
| Degree | Right.lateraloccipital_part2 | 76.453 | 12.271 | 77.686 | 12.932 | 0.882 | 0.696 |
| Degree | Right.lateraloccipital_part3 | 83.952 | 9.48 | 84.47 | 10.565 | 0.591 | 0.803 |
| Degree | Right.lateraloccipital_part4 | 71.614 | 9.51 | 72.467 | 10.226 | 0.952 | 0.674 |
| Degree | Right.lateraloccipital_part5 | 62.184 | 10.091 | 62.942 | 10.212 | 0.777 | 0.73 |
| Degree | Right.lateraloccipital_part6 | 75.571 | 5.995 | 75.601 | 6.495 | 0.23 | 0.92 |
| Degree | Right.lateraloccipital_part7 | 70.419 | 9.661 | 71.556 | 9.532 | 1.127 | 0.63 |
| Degree | Right.lateraloccipital_part8 | 76.766 | 6.496 | 77.099 | 6.451 | 0.533 | 0.824 |
| Degree | Right.lateraloccipital_part9 | 75.354 | 8.501 | 74.762 | 7.378 | -0.692 | 0.762 |
| Degree | Right.lateralorbitofrontal_part1 | 68.549 | 7.67 | 68.918 | 7.562 | 0.488 | 0.841 |
| Degree | Right.lateralorbitofrontal_part2 | 73.923 | 8.47 | 74.025 | 8.216 | 0.24 | 0.92 |
| Degree | Right.lateralorbitofrontal_part3 | 73.515 | 7.436 | 73.133 | 7.718 | -0.46 | 0.848 |
| Degree | Right.lateralorbitofrontal_part4 | 74.987 | 7.307 | 74.584 | 7.044 | -0.484 | 0.841 |
| Degree | Right.lingual_part1 | 69.648 | 8.754 | 69.813 | 8.107 | 0.26 | 0.915 |
| Degree | Right.lingual_part2 | 65.13 | 9.513 | 65.98 | 10.518 | 0.677 | 0.772 |
| Degree | Right.lingual_part3 | 70.709 | 9.03 | 72.138 | 9.333 | 1.585 | 0.472 |
| Degree | Right.lingual_part4 | 76.969 | 12.697 | 76.667 | 14.345 | -0.096 | 0.963 |
| Degree | Right.lingual_part5 | 70.547 | 8.93 | 70.128 | 8.971 | -0.401 | 0.872 |
| Degree | Right.lingual_part6 | 70.059 | 9.578 | 70.088 | 9.044 | 0.084 | 0.967 |
| Degree | Right.medialorbitofrontal_part1 | 75.177 | 6.263 | 76.048 | 6.226 | 1.762 | 0.425 |
| Degree | Right.medialorbitofrontal_part2 | 72.672 | 10.277 | 73.154 | 9.542 | 0.586 | 0.804 |
| Degree | Right.medialorbitofrontal_part3 | 76.77 | 5.784 | 77.093 | 5.009 | 0.63 | 0.789 |
| Degree | Right.middletemporal_part1 | 72.73 | 6.804 | 73.99 | 6.04 | 2.001 | 0.357 |
| Degree | Right.middletemporal_part2 | 73.984 | 6.886 | 75.408 | 6.248 | 2.201 | 0.309 |
| Degree | Right.middletemporal_part3 | 71.694 | 10.775 | 69.413 | 11.747 | -2.18 | 0.314 |
| Degree | Right.middletemporal_part4 | 58.475 | 11.196 | 60.179 | 12.048 | 1.439 | 0.525 |
| Degree | Right.middletemporal_part5 | 71.475 | 9.229 | 72.173 | 9.038 | 0.778 | 0.73 |
| Degree | Right.middletemporal_part6 | 80.243 | 10.244 | 79.711 | 9.673 | -0.511 | 0.829 |
| Degree | Right.parahippocampal_part1 | 76.307 | 10.827 | 76.453 | 9.646 | 0.035 | 0.987 |
| Degree | Right.parahippocampal_part2 | 79.628 | 11.619 | 79.181 | 11.282 | -0.568 | 0.814 |
| Degree | Right.paracentral_part1 | 75.597 | 9.13 | 75.896 | 8.413 | 0.274 | 0.914 |
| Degree | Right.paracentral_part2 | 72.887 | 7.601 | 72.413 | 7.722 | -0.599 | 0.8 |
| Degree | Right.paracentral_part3 | 83.193 | 10.981 | 82.044 | 12.041 | -0.98 | 0.673 |
| Degree | Right.parsopercularis_part1 | 85.412 | 11.181 | 85.783 | 10.909 | 0.299 | 0.907 |
| Degree | Right.parsopercularis_part2 | 82.353 | 15.128 | 76.147 | 17.351 | -3.692 | 0.034 |
| Degree | Right.parsopercularis_part3 | 87.756 | 12.049 | 84.045 | 14.711 | -2.783 | 0.188 |
| Degree | Right.parsorbitalis_part1 | 84.388 | 9.757 | 84.022 | 10.195 | -0.503 | 0.831 |
| Degree | Right.parstriangularis_part1 | 85.407 | 9.793 | 85.964 | 10.791 | 0.634 | 0.788 |
| Degree | Right.parstriangularis_part2 | 82.111 | 11.522 | 83.72 | 10.776 | 1.369 | 0.546 |
| Degree | Right.parstriangularis_part3 | 79.324 | 10.837 | 77.463 | 12.697 | -1.754 | 0.429 |
| Degree | Right.pericalcarine_part1 | 91.308 | 10.574 | 90.745 | 10.783 | -0.606 | 0.8 |
| Degree | Right.pericalcarine_part2 | 77.865 | 5.663 | 77.946 | 5.559 | 0.151 | 0.941 |
| Degree | Right.pericalcarine_part3 | 71.437 | 9.831 | 73.012 | 8.859 | 1.478 | 0.521 |
| Degree | Right.postcentral_part1 | 86.154 | 12.002 | 87.153 | 11.582 | 1.123 | 0.63 |
| Degree | Right.postcentral_part2 | 72.451 | 11.001 | 72.926 | 9.565 | 0.379 | 0.878 |
| Degree | Right.postcentral_part3 | 74.193 | 6.432 | 74.287 | 5.78 | 0.005 | 0.999 |
| Degree | Right.postcentral_part4 | 75.597 | 6.222 | 75.083 | 5.649 | -0.937 | 0.679 |
| Degree | Right.postcentral_part5 | 74.485 | 6.363 | 74.744 | 6.527 | 0.275 | 0.914 |
| Degree | Right.postcentral_part6 | 74.767 | 6.78 | 72.725 | 8.739 | -2.68 | 0.188 |
| Degree | Right.postcentral_part7 | 75.436 | 11.452 | 77.143 | 11.807 | 1.458 | 0.522 |
| Degree | Right.postcentral_part8 | 82.069 | 10.079 | 80.313 | 9.868 | -1.774 | 0.424 |
| Degree | Right.posteriorcingulate_part1 | 67.03 | 10.837 | 68.151 | 10.811 | 0.981 | 0.673 |
| Degree | Right.posteriorcingulate_part2 | 84.759 | 13.936 | 81.802 | 13.223 | -2.079 | 0.337 |
| Degree | Right.precentral_part1 | 73.036 | 11.566 | 72.002 | 11.541 | -1.059 | 0.653 |
| Degree | Right.precentral_part2 | 77.479 | 11.585 | 75.936 | 12.115 | -1.303 | 0.568 |
| Degree | Right.precentral_part3 | 77.592 | 11.038 | 77.102 | 11.646 | -0.461 | 0.848 |
| Degree | Right.precentral_part4 | 66.598 | 11.848 | 67.377 | 12.051 | 0.757 | 0.735 |
| Degree | Right.precentral_part5 | 49.513 | 10.017 | 51.929 | 9.687 | 2.668 | 0.188 |
| Degree | Right.precentral_part6 | 88.588 | 10.861 | 88.228 | 11.846 | -0.3 | 0.907 |
| Degree | Right.precentral_part7 | 85.013 | 15.279 | 80.611 | 15.696 | -2.731 | 0.188 |
| Degree | Right.precentral_part8 | 84.958 | 12.469 | 85.888 | 11.947 | 0.817 | 0.712 |
| Degree | Right.precentral_part9 | 89.282 | 13.959 | 86.104 | 14.598 | -2.231 | 0.296 |
| Degree | Right.precuneus_part1 | 89.995 | 13.624 | 90.529 | 12.161 | 0.373 | 0.878 |
| Degree | Right.precuneus_part2 | 63.853 | 17.871 | 66.362 | 16.433 | 1.502 | 0.515 |
| Degree | Right.precuneus_part3 | 88.536 | 15.431 | 88.191 | 14.318 | -0.375 | 0.878 |
| Degree | Right.precuneus_part4 | 74.467 | 13.388 | 75.047 | 13.047 | 0.454 | 0.85 |
| Degree | Right.precuneus_part5 | 89.581 | 13.2 | 86.404 | 15.709 | -2.155 | 0.317 |
| Degree | Right.precuneus_part6 | 67.769 | 11.533 | 67.492 | 10.839 | -0.324 | 0.898 |
| Degree | Right.precuneus_part7 | 76.002 | 11.882 | 76.62 | 11.764 | 0.699 | 0.76 |
| Degree | Right.rostralanteriorcingulate_part1 | 82.648 | 10.975 | 82.361 | 11.061 | -0.402 | 0.872 |
| Degree | Right.rostralmiddlefrontal_part1 | 72.622 | 10.811 | 72.315 | 10.039 | -0.128 | 0.949 |
| Degree | Right.rostralmiddlefrontal_part2 | 73.512 | 9.541 | 74.596 | 8.956 | 1.13 | 0.63 |
| Degree | Right.rostralmiddlefrontal_part3 | 74.788 | 10.279 | 75.147 | 10.376 | 0.27 | 0.914 |
| Degree | Right.rostralmiddlefrontal_part4 | 75.764 | 8.369 | 76.273 | 9.031 | 0.574 | 0.812 |
| Degree | Right.rostralmiddlefrontal_part5 | 69.888 | 12.233 | 72.401 | 11.476 | 2.193 | 0.312 |
| Degree | Right.rostralmiddlefrontal_part6 | 72.11 | 8.673 | 72.606 | 7.763 | 0.457 | 0.848 |
| Degree | Right.rostralmiddlefrontal_part7 | 72.176 | 10.406 | 73.396 | 10.012 | 1.129 | 0.63 |
| Degree | Right.rostralmiddlefrontal_part8 | 74.729 | 7.958 | 75.377 | 7.648 | 0.694 | 0.762 |
| Degree | Right.rostralmiddlefrontal_part9 | 74.06 | 11.884 | 75.539 | 10.022 | 1.414 | 0.533 |
| Degree | Right.rostralmiddlefrontal_part10 | 76.24 | 9.656 | 76.38 | 9.11 | 0.086 | 0.966 |
| Degree | Right.superiorfrontal_part1 | 74.451 | 11.238 | 73.438 | 11.08 | -1.045 | 0.657 |
| Degree | Right.superiorfrontal_part2 | 74.1 | 8.442 | 74.08 | 8.978 | -0.145 | 0.942 |
| Degree | Right.superiorfrontal_part3 | 74.159 | 9.455 | 74.426 | 9.579 | 0.21 | 0.93 |
| Degree | Right.superiorfrontal_part4 | 72.571 | 11.279 | 73.355 | 9.781 | 0.7 | 0.76 |
| Degree | Right.superiorfrontal_part5 | 74.279 | 10.245 | 74.581 | 9.028 | 0.339 | 0.891 |
| Degree | Right.superiorfrontal_part6 | 88.361 | 12.24 | 87.022 | 12.478 | -1.057 | 0.653 |
| Degree | Right.superiorfrontal_part7 | 85.433 | 12.561 | 86.464 | 11.25 | 0.901 | 0.696 |
| Degree | Right.superiorfrontal_part8 | 80.594 | 13.029 | 80.334 | 13.536 | -0.296 | 0.907 |
| Degree | Right.superiorfrontal_part9 | 81.758 | 15.434 | 82.476 | 13.763 | 0.561 | 0.817 |
| Degree | Right.superiorfrontal_part10 | 88.807 | 12.638 | 87.387 | 13.062 | -1.085 | 0.644 |
| Degree | Right.superiorfrontal_part11 | 70.889 | 10.851 | 70.651 | 8.908 | -0.149 | 0.941 |
| Degree | Right.superiorfrontal_part12 | 87.602 | 12.02 | 86.128 | 12.831 | -1.23 | 0.591 |
| Degree | Right.superiorfrontal_part13 | 71.964 | 13.4 | 73.482 | 12.709 | 1.311 | 0.567 |
| Degree | Right.superiorparietal_part1 | 92.948 | 11.251 | 91.35 | 11.735 | -1.388 | 0.541 |
| Degree | Right.superiorparietal_part2 | 71.112 | 12.394 | 72.788 | 13.675 | 1.6 | 0.469 |
| Degree | Right.superiorparietal_part3 | 86.57 | 13.109 | 85.582 | 13.359 | -0.589 | 0.803 |
| Degree | Right.superiorparietal_part4 | 79.238 | 14.96 | 78.965 | 14.914 | -0.132 | 0.948 |
| Degree | Right.superiorparietal_part5 | 88.214 | 11.75 | 86.633 | 12.445 | -1.286 | 0.575 |
| Degree | Right.superiorparietal_part6 | 75.648 | 10.06 | 74.835 | 8.87 | -0.809 | 0.714 |
| Degree | Right.superiorparietal_part7 | 78.75 | 10.388 | 78.336 | 10.278 | -0.441 | 0.852 |
| Degree | Right.superiorparietal_part8 | 80.728 | 9.868 | 78.103 | 9.914 | -2.781 | 0.188 |
| Degree | Right.superiorparietal_part9 | 70.509 | 9.921 | 70.277 | 10.214 | -0.314 | 0.902 |
| Degree | Right.superiorparietal_part10 | 72.188 | 10.944 | 72.195 | 9.616 | 0.039 | 0.986 |
| Degree | Right.superiortemporal_part1 | 78.618 | 10.482 | 77.919 | 10.122 | -0.759 | 0.735 |
| Degree | Right.superiortemporal_part2 | 78.418 | 9.763 | 77.663 | 10.27 | -0.972 | 0.673 |
| Degree | Right.superiortemporal_part3 | 73.391 | 11.313 | 75.035 | 11.632 | 1.462 | 0.522 |
| Degree | Right.superiortemporal_part4 | 77.952 | 9.646 | 78.266 | 9.068 | 0.3 | 0.907 |
| Degree | Right.superiortemporal_part5 | 77.124 | 12.261 | 75.491 | 11.374 | -1.611 | 0.469 |
| Degree | Right.superiortemporal_part6 | 86.223 | 10.011 | 87.155 | 8.881 | 1.052 | 0.654 |
| Degree | Right.supramarginal_part1 | 82.258 | 10.653 | 81.597 | 9.993 | -0.72 | 0.75 |
| Degree | Right.supramarginal_part2 | 86.876 | 10.067 | 87.387 | 9.816 | 0.505 | 0.83 |
| Degree | Right.supramarginal_part3 | 91.277 | 9.965 | 90.692 | 11.827 | -0.609 | 0.8 |
| Degree | Right.supramarginal_part4 | 81.32 | 10.43 | 81.53 | 11.218 | 0.29 | 0.91 |
| Degree | Right.supramarginal_part5 | 97.098 | 7.486 | 95.945 | 7.375 | -1.495 | 0.517 |
| Degree | Right.supramarginal_part6 | 80.71 | 10.265 | 79.861 | 10.464 | -0.961 | 0.673 |
| Degree | Right.supramarginal_part7 | 70.473 | 8.864 | 72.001 | 11.075 | 1.47 | 0.522 |
| Degree | Right.frontalpole_part1 | 75.16 | 11.115 | 75.337 | 10.449 | -0.043 | 0.985 |
| Degree | Right.temporalpole_part1 | 75.834 | 11.084 | 76.665 | 12.071 | 0.657 | 0.783 |
| Degree | Right.transversetemporal_part1 | 72.009 | 10.438 | 72.118 | 10.757 | -0.122 | 0.951 |
| Degree | Right.insula_part1 | 73.326 | 13.396 | 72.383 | 12.39 | -1.077 | 0.644 |
| Degree | Right.insula_part2 | 87.991 | 13.175 | 88.971 | 12.661 | 0.764 | 0.732 |
| Degree | Right.insula_part3 | 68.934 | 12.14 | 69.743 | 12.124 | 0.419 | 0.861 |
| Degree | Right.insula_part4 | 82.538 | 9.203 | 84.172 | 8.508 | 1.844 | 0.413 |
| Betweenness centrality | Left.bankssts_part1 | 151.994 | 44.117 | 148.74 | 38.934 | -0.928 | 0.683 |
| Betweenness centrality | Left.bankssts_part2 | 156.526 | 43.017 | 149.215 | 35.464 | -1.961 | 0.367 |
| Betweenness centrality | Left.caudalanteriorcingulate_part1 | 125.307 | 39.134 | 127.353 | 39.314 | 0.531 | 0.824 |
| Betweenness centrality | Left.caudalmiddlefrontal_part1 | 157.265 | 46.518 | 148.124 | 38.863 | -2.206 | 0.309 |
| Betweenness centrality | Left.caudalmiddlefrontal_part2 | 143.617 | 39.728 | 142.555 | 39.378 | -0.403 | 0.872 |
| Betweenness centrality | Left.caudalmiddlefrontal_part3 | 148.606 | 38.213 | 152.298 | 39.702 | 0.986 | 0.673 |
| Betweenness centrality | Left.caudalmiddlefrontal_part4 | 145.734 | 46.151 | 142.964 | 41.66 | -0.583 | 0.805 |
| Betweenness centrality | Left.cuneus_part1 | 183.099 | 46.713 | 179.16 | 46.23 | -0.801 | 0.719 |
| Betweenness centrality | Left.cuneus_part2 | 153.986 | 42.827 | 148.639 | 37.513 | -1.298 | 0.57 |
| Betweenness centrality | Left.entorhinal_part1 | 96.974 | 27.982 | 101.503 | 27.727 | 1.526 | 0.502 |
| Betweenness centrality | Left.fusiform_part1 | 179.917 | 45.801 | 189.358 | 51.786 | 1.989 | 0.36 |
| Betweenness centrality | Left.fusiform_part2 | 151.662 | 46.557 | 163.284 | 46.616 | 2.529 | 0.212 |
| Betweenness centrality | Left.fusiform_part3 | 152.605 | 50.078 | 151.581 | 43.241 | -0.107 | 0.96 |
| Betweenness centrality | Left.fusiform_part4 | 147.812 | 43.882 | 155.541 | 44.052 | 1.906 | 0.39 |
| Betweenness centrality | Left.fusiform_part5 | 148.853 | 46.42 | 155.777 | 44.323 | 1.657 | 0.457 |
| Betweenness centrality | Left.inferiorparietal_part1 | 151.518 | 43.614 | 161.604 | 44.316 | 2.35 | 0.259 |
| Betweenness centrality | Left.inferiorparietal_part2 | 155.345 | 50.561 | 151.588 | 45.62 | -0.653 | 0.785 |
| Betweenness centrality | Left.inferiorparietal_part3 | 145.981 | 40.521 | 158.572 | 48.473 | 2.764 | 0.188 |
| Betweenness centrality | Left.inferiorparietal_part4 | 152.296 | 43.183 | 153.339 | 47.677 | 0.095 | 0.963 |
| Betweenness centrality | Left.inferiorparietal_part5 | 155.858 | 48.465 | 148.534 | 44.173 | -1.527 | 0.502 |
| Betweenness centrality | Left.inferiorparietal_part6 | 155.687 | 42.714 | 153.54 | 38.158 | -0.27 | 0.914 |
| Betweenness centrality | Left.inferiorparietal_part7 | 137.714 | 37.973 | 138.515 | 39.197 | 0.205 | 0.932 |
| Betweenness centrality | Left.inferiorparietal_part8 | 141.459 | 41.352 | 137.151 | 36.854 | -1.036 | 0.657 |
| Betweenness centrality | Left.inferiortemporal_part1 | 147.077 | 45.591 | 142.439 | 43.124 | -1.079 | 0.644 |
| Betweenness centrality | Left.inferiortemporal_part2 | 165.111 | 55.844 | 158.443 | 46.467 | -1.308 | 0.567 |
| Betweenness centrality | Left.inferiortemporal_part3 | 150.368 | 38.021 | 144.282 | 42.83 | -1.6 | 0.469 |
| Betweenness centrality | Left.inferiortemporal_part4 | 151.307 | 45.104 | 147.65 | 46.995 | -0.793 | 0.724 |
| Betweenness centrality | Left.inferiortemporal_part5 | 157.53 | 49.121 | 155.416 | 51.337 | -0.423 | 0.859 |
| Betweenness centrality | Left.inferiortemporal_part6 | 145.572 | 46.035 | 147.414 | 43.464 | 0.53 | 0.825 |
| Betweenness centrality | Left.isthmuscingulate_part1 | 155.133 | 46.357 | 151.899 | 48.465 | -0.707 | 0.758 |
| Betweenness centrality | Left.isthmuscingulate_part2 | 107.348 | 37.824 | 107.963 | 39.245 | 0.227 | 0.922 |
| Betweenness centrality | Left.lateraloccipital_part1 | 135.145 | 44.261 | 132.586 | 36.265 | -0.802 | 0.719 |
| Betweenness centrality | Left.lateraloccipital_part2 | 108.64 | 36.039 | 106.253 | 35.316 | -0.588 | 0.803 |
| Betweenness centrality | Left.lateraloccipital_part3 | 109.742 | 30.975 | 111.666 | 27.842 | 0.647 | 0.786 |
| Betweenness centrality | Left.lateraloccipital_part4 | 155.191 | 47.041 | 151.765 | 42.915 | -0.772 | 0.73 |
| Betweenness centrality | Left.lateraloccipital_part5 | 142.434 | 45.5 | 139.186 | 40.431 | -0.84 | 0.705 |
| Betweenness centrality | Left.lateraloccipital_part6 | 154.291 | 40.159 | 143.954 | 42.88 | -2.654 | 0.188 |
| Betweenness centrality | Left.lateraloccipital_part7 | 144.323 | 41.491 | 146.279 | 38.732 | 0.56 | 0.817 |
| Betweenness centrality | Left.lateraloccipital_part8 | 132.476 | 31.063 | 130.577 | 31.879 | -0.706 | 0.758 |
| Betweenness centrality | Left.lateraloccipital_part9 | 144.559 | 37.109 | 143.564 | 37.718 | -0.162 | 0.941 |
| Betweenness centrality | Left.lateralorbitofrontal_part1 | 125.661 | 28.457 | 130.608 | 30.099 | 1.742 | 0.429 |
| Betweenness centrality | Left.lateralorbitofrontal_part2 | 143.325 | 42.184 | 138.975 | 39.465 | -0.88 | 0.696 |
| Betweenness centrality | Left.lateralorbitofrontal_part3 | 151.444 | 44.952 | 153.212 | 51.384 | 0.426 | 0.859 |
| Betweenness centrality | Left.lateralorbitofrontal_part4 | 135.918 | 36.483 | 133.764 | 33.394 | -0.519 | 0.827 |
| Betweenness centrality | Left.lingual_part1 | 142.054 | 38.165 | 138.035 | 36.483 | -1.119 | 0.63 |
| Betweenness centrality | Left.lingual_part2 | 135.681 | 35.461 | 142.327 | 36.348 | 1.846 | 0.413 |
| Betweenness centrality | Left.lingual_part3 | 143.78 | 42.732 | 143.271 | 42.445 | -0.203 | 0.932 |
| Betweenness centrality | Left.lingual_part4 | 138.889 | 38.753 | 140.115 | 38.804 | 0.262 | 0.915 |
| Betweenness centrality | Left.lingual_part5 | 155.846 | 51.304 | 154.654 | 53.724 | -0.253 | 0.919 |
| Betweenness centrality | Left.lingual_part6 | 165.705 | 54.085 | 158.136 | 47.702 | -1.468 | 0.522 |
| Betweenness centrality | Left.medialorbitofrontal_part1 | 115.505 | 29.695 | 115.058 | 30.659 | -0.335 | 0.893 |
| Betweenness centrality | Left.medialorbitofrontal_part2 | 157.329 | 55.458 | 155.334 | 55.531 | -0.28 | 0.914 |
| Betweenness centrality | Left.medialorbitofrontal_part3 | 112.613 | 29.112 | 112.459 | 27.855 | -0.152 | 0.941 |
| Betweenness centrality | Left.middletemporal_part1 | 165.84 | 57.879 | 164.451 | 52.776 | -0.138 | 0.945 |
| Betweenness centrality | Left.middletemporal_part2 | 129.511 | 39.706 | 128.991 | 37.857 | 0.028 | 0.987 |
| Betweenness centrality | Left.middletemporal_part3 | 113.077 | 53.483 | 117.769 | 46.733 | 0.749 | 0.736 |
| Betweenness centrality | Left.middletemporal_part4 | 140.412 | 49.64 | 148.757 | 49.954 | 1.833 | 0.418 |
| Betweenness centrality | Left.middletemporal_part5 | 114.009 | 41.486 | 122.675 | 44.288 | 2.027 | 0.348 |
| Betweenness centrality | Left.parahippocampal_part1 | 171.328 | 55.043 | 161.103 | 46.859 | -2.021 | 0.348 |
| Betweenness centrality | Left.parahippocampal_part2 | 142.318 | 37.123 | 146.413 | 36.516 | 1.138 | 0.627 |
| Betweenness centrality | Left.paracentral_part1 | 167.845 | 49.593 | 158.821 | 45.597 | -1.939 | 0.379 |
| Betweenness centrality | Left.paracentral_part2 | 154.501 | 41.314 | 154.076 | 40.765 | 0.043 | 0.985 |
| Betweenness centrality | Left.paracentral_part3 | 169.346 | 44.449 | 163.905 | 52.332 | -1.2 | 0.598 |
| Betweenness centrality | Left.parsopercularis_part1 | 167.561 | 47.567 | 171.696 | 46.537 | 0.965 | 0.673 |
| Betweenness centrality | Left.parsopercularis_part2 | 130.223 | 36.263 | 124.485 | 29.406 | -1.863 | 0.407 |
| Betweenness centrality | Left.parsopercularis_part3 | 128.201 | 38.233 | 133.822 | 44.068 | 1.372 | 0.546 |
| Betweenness centrality | Left.parsorbitalis_part1 | 144.782 | 35.342 | 136.969 | 33.483 | -2.508 | 0.219 |
| Betweenness centrality | Left.parstriangularis_part1 | 141.044 | 36.988 | 136.354 | 31.954 | -1.328 | 0.565 |
| Betweenness centrality | Left.parstriangularis_part2 | 169.559 | 48.028 | 160.955 | 45.705 | -2.068 | 0.34 |
| Betweenness centrality | Left.pericalcarine_part1 | 155.909 | 41.543 | 146.329 | 40.364 | -2.37 | 0.259 |
| Betweenness centrality | Left.pericalcarine_part2 | 155.488 | 36.573 | 155.543 | 39.198 | 0.00E+00 | 1 |
| Betweenness centrality | Left.postcentral_part1 | 149.087 | 34.425 | 146.325 | 32.219 | -0.881 | 0.696 |
| Betweenness centrality | Left.postcentral_part2 | 170.02 | 41.858 | 163.62 | 43.433 | -1.701 | 0.449 |
| Betweenness centrality | Left.postcentral_part3 | 155.328 | 45.06 | 157.349 | 45.651 | 0.521 | 0.827 |
| Betweenness centrality | Left.postcentral_part4 | 132.956 | 35.76 | 137.533 | 42.469 | 1.143 | 0.627 |
| Betweenness centrality | Left.postcentral_part5 | 143.977 | 39.11 | 140.542 | 35.34 | -0.87 | 0.697 |
| Betweenness centrality | Left.postcentral_part6 | 162.524 | 43.737 | 163.238 | 46.647 | 0.368 | 0.879 |
| Betweenness centrality | Left.postcentral_part7 | 168.637 | 46.937 | 164.61 | 41.993 | -0.958 | 0.674 |
| Betweenness centrality | Left.postcentral_part8 | 152.832 | 42.075 | 159.196 | 43.02 | 1.507 | 0.514 |
| Betweenness centrality | Left.posteriorcingulate_part1 | 152.087 | 48.966 | 155.335 | 42.578 | 0.776 | 0.73 |
| Betweenness centrality | Left.posteriorcingulate_part2 | 137.875 | 36.491 | 133.634 | 38.797 | -1.161 | 0.618 |
| Betweenness centrality | Left.precentral_part1 | 170.765 | 53.57 | 172.966 | 54.503 | 0.339 | 0.891 |
| Betweenness centrality | Left.precentral_part2 | 163.375 | 44.927 | 155.36 | 42.523 | -1.666 | 0.457 |
| Betweenness centrality | Left.precentral_part3 | 157.895 | 52.344 | 151.449 | 47.957 | -1.224 | 0.591 |
| Betweenness centrality | Left.precentral_part4 | 153.899 | 45.161 | 154.412 | 47.286 | 0.16 | 0.941 |
| Betweenness centrality | Left.precentral_part5 | 133.05 | 37.165 | 128.115 | 36.517 | -1.469 | 0.522 |
| Betweenness centrality | Left.precentral_part6 | 115.842 | 42.656 | 115.891 | 44.86 | 0.073 | 0.972 |
| Betweenness centrality | Left.precentral_part7 | 162.436 | 42.448 | 155.481 | 37.465 | -1.87 | 0.407 |
| Betweenness centrality | Left.precentral_part8 | 124.316 | 35.956 | 122.755 | 35.002 | -0.174 | 0.941 |
| Betweenness centrality | Left.precentral_part9 | 164.587 | 44.997 | 158.154 | 40.875 | -1.458 | 0.522 |
| Betweenness centrality | Left.precuneus_part1 | 136.068 | 37.197 | 132.871 | 37.632 | -0.778 | 0.73 |
| Betweenness centrality | Left.precuneus_part2 | 121.797 | 34.16 | 119.919 | 30.296 | -0.462 | 0.848 |
| Betweenness centrality | Left.precuneus_part3 | 145.277 | 39.298 | 143.361 | 33.883 | -0.604 | 0.8 |
| Betweenness centrality | Left.precuneus_part4 | 153.659 | 43.453 | 144.538 | 42.477 | -2.315 | 0.26 |
| Betweenness centrality | Left.precuneus_part5 | 130.044 | 40.726 | 130.887 | 37.334 | 0.235 | 0.92 |
| Betweenness centrality | Left.precuneus_part6 | 128.886 | 50.893 | 132.397 | 50.443 | 0.637 | 0.786 |
| Betweenness centrality | Left.precuneus_part7 | 164.267 | 47.438 | 158.502 | 44.665 | -1.158 | 0.618 |
| Betweenness centrality | Left.rostralanteriorcingulate_part1 | 162.573 | 48.144 | 164.318 | 47.446 | 0.449 | 0.851 |
| Betweenness centrality | Left.rostralmiddlefrontal_part1 | 163.485 | 46.261 | 158.881 | 44.202 | -1.084 | 0.644 |
| Betweenness centrality | Left.rostralmiddlefrontal_part2 | 153.225 | 45.851 | 159.092 | 42.942 | 1.292 | 0.572 |
| Betweenness centrality | Left.rostralmiddlefrontal_part3 | 160.886 | 51.941 | 164.256 | 46.689 | 0.838 | 0.705 |
| Betweenness centrality | Left.rostralmiddlefrontal_part4 | 150.915 | 44.258 | 155.493 | 42.704 | 0.998 | 0.673 |
| Betweenness centrality | Left.rostralmiddlefrontal_part5 | 151.208 | 45.392 | 149.976 | 36.721 | -0.213 | 0.93 |
| Betweenness centrality | Left.rostralmiddlefrontal_part6 | 160.182 | 45.648 | 162.532 | 44.536 | 0.535 | 0.824 |
| Betweenness centrality | Left.rostralmiddlefrontal_part7 | 161.252 | 52.814 | 165.173 | 56.009 | 0.584 | 0.805 |
| Betweenness centrality | Left.rostralmiddlefrontal_part8 | 143.116 | 40.91 | 142.535 | 44.349 | 0 | 1 |
| Betweenness centrality | Left.rostralmiddlefrontal_part9 | 152.657 | 44.272 | 152.347 | 48.866 | 0.052 | 0.983 |
| Betweenness centrality | Left.rostralmiddlefrontal_part10 | 141.967 | 36.685 | 141.97 | 34.567 | -0.108 | 0.96 |
| Betweenness centrality | Left.superiorfrontal_part1 | 157.806 | 45.815 | 155.027 | 48.695 | -0.75 | 0.736 |
| Betweenness centrality | Left.superiorfrontal_part2 | 147.069 | 41.091 | 150.941 | 41.025 | 0.946 | 0.674 |
| Betweenness centrality | Left.superiorfrontal_part3 | 161.32 | 51.505 | 160.811 | 56.065 | -0.099 | 0.962 |
| Betweenness centrality | Left.superiorfrontal_part4 | 149.605 | 46.718 | 151.12 | 46.771 | 0.236 | 0.92 |
| Betweenness centrality | Left.superiorfrontal_part5 | 147.678 | 40.498 | 154.233 | 45.379 | 1.65 | 0.457 |
| Betweenness centrality | Left.superiorfrontal_part6 | 147.824 | 43.705 | 146.304 | 40.86 | -0.387 | 0.877 |
| Betweenness centrality | Left.superiorfrontal_part7 | 135.687 | 40.806 | 137.795 | 38.645 | 0.546 | 0.819 |
| Betweenness centrality | Left.superiorfrontal_part8 | 146.275 | 42.171 | 147.541 | 38.501 | 0.17 | 0.941 |
| Betweenness centrality | Left.superiorfrontal_part9 | 138.205 | 41.183 | 138.489 | 42.729 | -0.027 | 0.987 |
| Betweenness centrality | Left.superiorfrontal_part10 | 138.378 | 34.365 | 135.533 | 38.634 | -0.782 | 0.729 |
| Betweenness centrality | Left.superiorfrontal_part11 | 134.92 | 37.256 | 134.94 | 37.827 | -0.101 | 0.962 |
| Betweenness centrality | Left.superiorfrontal_part12 | 148.04 | 48.593 | 154.662 | 53.934 | 1.381 | 0.541 |
| Betweenness centrality | Left.superiorfrontal_part13 | 137.44 | 36.259 | 143.821 | 42.49 | 1.608 | 0.469 |
| Betweenness centrality | Left.superiorparietal_part1 | 137.882 | 50.793 | 138.21 | 50.364 | -0.027 | 0.987 |
| Betweenness centrality | Left.superiorparietal_part2 | 126.924 | 33.749 | 128.554 | 34.741 | 0.45 | 0.851 |
| Betweenness centrality | Left.superiorparietal_part3 | 155.017 | 45.504 | 148.592 | 42.473 | -1.626 | 0.469 |
| Betweenness centrality | Left.superiorparietal_part4 | 131.391 | 39.523 | 133.012 | 39.62 | 0.536 | 0.824 |
| Betweenness centrality | Left.superiorparietal_part5 | 142.798 | 44.928 | 143.499 | 41.731 | 0.21 | 0.93 |
| Betweenness centrality | Left.superiorparietal_part6 | 171.423 | 53.575 | 170.241 | 49.216 | -0.166 | 0.941 |
| Betweenness centrality | Left.superiorparietal_part7 | 144.205 | 46.118 | 144.908 | 41.933 | 0.188 | 0.935 |
| Betweenness centrality | Left.superiorparietal_part8 | 155.731 | 49.446 | 154.589 | 53.796 | -0.266 | 0.915 |
| Betweenness centrality | Left.superiorparietal_part9 | 175.922 | 48.686 | 165.484 | 49.931 | -2.173 | 0.314 |
| Betweenness centrality | Left.superiorparietal_part10 | 192.225 | 49.922 | 181.885 | 50.175 | -2.179 | 0.314 |
| Betweenness centrality | Left.superiortemporal_part1 | 109.215 | 49.708 | 86.631 | 53.197 | -4.585 | 0.002 |
| Betweenness centrality | Left.superiortemporal_part2 | 154.565 | 48.108 | 157.891 | 44.01 | 0.926 | 0.684 |
| Betweenness centrality | Left.superiortemporal_part3 | 151.214 | 45.76 | 155.75 | 45.029 | 0.964 | 0.673 |
| Betweenness centrality | Left.superiortemporal_part4 | 178.085 | 44.255 | 175.964 | 46.102 | -0.602 | 0.8 |
| Betweenness centrality | Left.superiortemporal_part5 | 177.991 | 48.061 | 171.215 | 42.978 | -1.418 | 0.531 |
| Betweenness centrality | Left.superiortemporal_part6 | 156.309 | 46.298 | 153.1 | 40.637 | -0.844 | 0.705 |
| Betweenness centrality | Left.superiortemporal_part7 | 145.237 | 35.979 | 146.622 | 39.312 | 0.298 | 0.907 |
| Betweenness centrality | Left.supramarginal_part1 | 168.354 | 47.929 | 167.054 | 52.546 | -0.232 | 0.92 |
| Betweenness centrality | Left.supramarginal_part2 | 152.24 | 38.471 | 152.606 | 39.103 | 0.26 | 0.915 |
| Betweenness centrality | Left.supramarginal_part3 | 165.677 | 43.266 | 169.842 | 47.648 | 0.85 | 0.705 |
| Betweenness centrality | Left.supramarginal_part4 | 151.316 | 43.932 | 158.058 | 43.032 | 1.679 | 0.456 |
| Betweenness centrality | Left.supramarginal_part5 | 147.58 | 37.754 | 142.724 | 29.84 | -1.503 | 0.515 |
| Betweenness centrality | Left.supramarginal_part6 | 173.034 | 51.77 | 164.282 | 40.57 | -1.927 | 0.382 |
| Betweenness centrality | Left.supramarginal_part7 | 165.037 | 46.839 | 163.958 | 45.296 | -0.222 | 0.925 |
| Betweenness centrality | Left.frontalpole_part1 | 150.331 | 52.777 | 149.591 | 38.501 | -0.239 | 0.92 |
| Betweenness centrality | Left.temporalpole_part1 | 162.913 | 41.488 | 159.352 | 40.496 | -0.847 | 0.705 |
| Betweenness centrality | Left.transversetemporal_part1 | 160.074 | 43.184 | 158.524 | 38.863 | -0.457 | 0.848 |
| Betweenness centrality | Left.insula_part1 | 170.971 | 45.904 | 160.727 | 44.313 | -2.487 | 0.219 |
| Betweenness centrality | Left.insula_part2 | 143.474 | 44.993 | 145.244 | 47.777 | 0.445 | 0.851 |
| Betweenness centrality | Left.insula_part3 | 145.92 | 41.154 | 146.52 | 40.209 | 1.00E-03 | 1 |
| Betweenness centrality | Left.insula_part4 | 189.941 | 59.359 | 185.962 | 58.583 | -0.683 | 0.768 |
| Betweenness centrality | Right.bankssts_part1 | 141.878 | 38.803 | 137.559 | 38.759 | -1.14 | 0.627 |
| Betweenness centrality | Right.bankssts_part2 | 144.024 | 40.763 | 143.096 | 39.514 | -0.232 | 0.92 |
| Betweenness centrality | Right.caudalanteriorcingulate_part1 | 134.985 | 35.627 | 136.944 | 33.418 | 0.643 | 0.786 |
| Betweenness centrality | Right.caudalmiddlefrontal_part1 | 142.034 | 39.15 | 145.549 | 40.692 | 1.07 | 0.648 |
| Betweenness centrality | Right.caudalmiddlefrontal_part2 | 139.569 | 38.962 | 133.812 | 39.941 | -1.404 | 0.536 |
| Betweenness centrality | Right.caudalmiddlefrontal_part3 | 138.981 | 44.488 | 135.821 | 38.319 | -0.643 | 0.786 |
| Betweenness centrality | Right.caudalmiddlefrontal_part4 | 143.162 | 44.341 | 145.363 | 41.825 | 0.52 | 0.827 |
| Betweenness centrality | Right.cuneus_part1 | 170.572 | 57.566 | 164.938 | 51.374 | -1.109 | 0.634 |
| Betweenness centrality | Right.cuneus_part2 | 130.657 | 43.604 | 126.609 | 39.516 | -0.889 | 0.696 |
| Betweenness centrality | Right.cuneus_part3 | 129.724 | 43.676 | 130.625 | 38.865 | 0.193 | 0.935 |
| Betweenness centrality | Right.entorhinal_part1 | 112.177 | 32.179 | 114.12 | 30.953 | 0.549 | 0.818 |
| Betweenness centrality | Right.fusiform_part1 | 174.292 | 44.384 | 174.571 | 45.321 | 0.173 | 0.941 |
| Betweenness centrality | Right.fusiform_part2 | 158.034 | 40.022 | 164.676 | 38.455 | 1.689 | 0.454 |
| Betweenness centrality | Right.fusiform_part3 | 138.927 | 45.077 | 138.615 | 45.245 | -0.038 | 0.986 |
| Betweenness centrality | Right.fusiform_part4 | 159.2 | 41.873 | 159.379 | 48.366 | -0.019 | 0.991 |
| Betweenness centrality | Right.fusiform_part5 | 166.706 | 50.941 | 164.155 | 56.837 | -0.715 | 0.753 |
| Betweenness centrality | Right.inferiorparietal_part1 | 158.998 | 47.665 | 160.839 | 47.201 | 0.375 | 0.878 |
| Betweenness centrality | Right.inferiorparietal_part2 | 156.592 | 43.015 | 156.529 | 49.038 | -0.152 | 0.941 |
| Betweenness centrality | Right.inferiorparietal_part3 | 155.243 | 46.567 | 145.205 | 39.713 | -2.485 | 0.219 |
| Betweenness centrality | Right.inferiorparietal_part4 | 148.472 | 39.221 | 153.684 | 44.559 | 1.139 | 0.627 |
| Betweenness centrality | Right.inferiorparietal_part5 | 161.489 | 46.942 | 155.711 | 47.828 | -1.335 | 0.561 |
| Betweenness centrality | Right.inferiorparietal_part6 | 157.411 | 48.416 | 154.838 | 48.459 | -0.555 | 0.818 |
| Betweenness centrality | Right.inferiorparietal_part7 | 153.302 | 37.758 | 157.37 | 45.943 | 1.083 | 0.644 |
| Betweenness centrality | Right.inferiorparietal_part8 | 169.092 | 42.691 | 165.907 | 45.993 | -0.954 | 0.674 |
| Betweenness centrality | Right.inferiorparietal_part9 | 138.773 | 41.119 | 143.48 | 43.801 | 1.12 | 0.63 |
| Betweenness centrality | Right.inferiorparietal_part10 | 145.775 | 37.928 | 145.281 | 40.046 | -0.074 | 0.972 |
| Betweenness centrality | Right.inferiortemporal_part1 | 142.392 | 44.494 | 142.728 | 42.172 | -0.076 | 0.972 |
| Betweenness centrality | Right.inferiortemporal_part2 | 167.606 | 44.544 | 160.349 | 49.242 | -1.616 | 0.469 |
| Betweenness centrality | Right.inferiortemporal_part3 | 147.609 | 35.837 | 148.221 | 41.68 | 0.183 | 0.936 |
| Betweenness centrality | Right.inferiortemporal_part4 | 144.6 | 50.434 | 148.008 | 49.503 | 0.703 | 0.758 |
| Betweenness centrality | Right.inferiortemporal_part5 | 150.534 | 46.298 | 152.57 | 48.192 | 0.52 | 0.827 |
| Betweenness centrality | Right.isthmuscingulate_part1 | 150.585 | 46.577 | 149.985 | 47.681 | -0.2 | 0.932 |
| Betweenness centrality | Right.isthmuscingulate_part2 | 132.81 | 33.862 | 132.915 | 30.301 | -0.044 | 0.985 |
| Betweenness centrality | Right.lateraloccipital_part1 | 100.495 | 34.955 | 103.739 | 33.429 | 0.814 | 0.712 |
| Betweenness centrality | Right.lateraloccipital_part2 | 116.027 | 34.03 | 113.045 | 32.734 | -0.989 | 0.673 |
| Betweenness centrality | Right.lateraloccipital_part3 | 117.476 | 28.848 | 117.889 | 27.689 | 0.242 | 0.92 |
| Betweenness centrality | Right.lateraloccipital_part4 | 162.265 | 49.491 | 156.711 | 43.671 | -1.129 | 0.63 |
| Betweenness centrality | Right.lateraloccipital_part5 | 149.185 | 48.214 | 151.726 | 51.434 | 0.432 | 0.856 |
| Betweenness centrality | Right.lateraloccipital_part6 | 132.552 | 33.8 | 130.486 | 29.239 | -0.737 | 0.742 |
| Betweenness centrality | Right.lateraloccipital_part7 | 151.84 | 43.276 | 151.384 | 46.259 | -0.268 | 0.914 |
| Betweenness centrality | Right.lateraloccipital_part8 | 134 | 32.293 | 135.555 | 36.65 | 0.525 | 0.826 |
| Betweenness centrality | Right.lateraloccipital_part9 | 153.311 | 39.435 | 148.961 | 44.303 | -1.027 | 0.657 |
| Betweenness centrality | Right.lateralorbitofrontal_part1 | 144.531 | 36.81 | 144.251 | 34.535 | -0.076 | 0.972 |
| Betweenness centrality | Right.lateralorbitofrontal_part2 | 156.99 | 42.7 | 152.274 | 46.114 | -1.191 | 0.603 |
| Betweenness centrality | Right.lateralorbitofrontal_part3 | 141.815 | 40.17 | 147.803 | 46.718 | 1.381 | 0.541 |
| Betweenness centrality | Right.lateralorbitofrontal_part4 | 135.888 | 34.191 | 141.117 | 35.42 | 1.434 | 0.525 |
| Betweenness centrality | Right.lingual_part1 | 143.079 | 40.712 | 140.178 | 35.81 | -0.859 | 0.702 |
| Betweenness centrality | Right.lingual_part2 | 144.065 | 51.236 | 137.75 | 51.258 | -1.477 | 0.521 |
| Betweenness centrality | Right.lingual_part3 | 137.96 | 37.94 | 136.645 | 32.187 | -0.437 | 0.854 |
| Betweenness centrality | Right.lingual_part4 | 151.579 | 50.59 | 143.9 | 52.24 | -1.568 | 0.483 |
| Betweenness centrality | Right.lingual_part5 | 146.073 | 41.637 | 151.552 | 46.727 | 1.217 | 0.591 |
| Betweenness centrality | Right.lingual_part6 | 149.066 | 50.68 | 155.478 | 50.707 | 1.371 | 0.546 |
| Betweenness centrality | Right.medialorbitofrontal_part1 | 114.698 | 37.639 | 117.77 | 29.631 | 0.814 | 0.712 |
| Betweenness centrality | Right.medialorbitofrontal_part2 | 159.114 | 54.659 | 161.71 | 59.973 | 0.539 | 0.824 |
| Betweenness centrality | Right.medialorbitofrontal_part3 | 107.225 | 28.502 | 108.22 | 28.179 | 0.342 | 0.891 |
| Betweenness centrality | Right.middletemporal_part1 | 141.206 | 53.428 | 140.556 | 51.298 | -0.164 | 0.941 |
| Betweenness centrality | Right.middletemporal_part2 | 135.81 | 48.876 | 137.709 | 50.138 | 0.554 | 0.818 |
| Betweenness centrality | Right.middletemporal_part3 | 138.151 | 41.488 | 132.244 | 44.266 | -1.451 | 0.524 |
| Betweenness centrality | Right.middletemporal_part4 | 105.211 | 38.987 | 113.108 | 41.909 | 1.806 | 0.424 |
| Betweenness centrality | Right.middletemporal_part5 | 126.18 | 36.466 | 126.908 | 36.808 | 0.189 | 0.935 |
| Betweenness centrality | Right.middletemporal_part6 | 183.592 | 53.915 | 178.701 | 43.874 | -0.832 | 0.706 |
| Betweenness centrality | Right.parahippocampal_part1 | 170.886 | 45.924 | 176.833 | 45.064 | 1.397 | 0.537 |
| Betweenness centrality | Right.parahippocampal_part2 | 172.653 | 50.734 | 167.833 | 46.9 | -0.957 | 0.674 |
| Betweenness centrality | Right.paracentral_part1 | 154.011 | 37.526 | 154.336 | 38.692 | 0.156 | 0.941 |
| Betweenness centrality | Right.paracentral_part2 | 157.355 | 46.169 | 153.19 | 41.833 | -0.886 | 0.696 |
| Betweenness centrality | Right.paracentral_part3 | 180.25 | 51.906 | 171.035 | 49.95 | -1.785 | 0.424 |
| Betweenness centrality | Right.parsopercularis_part1 | 154 | 42.753 | 160.172 | 43.33 | 1.564 | 0.483 |
| Betweenness centrality | Right.parsopercularis_part2 | 113.432 | 41.95 | 113.193 | 43.032 | 0.192 | 0.935 |
| Betweenness centrality | Right.parsopercularis_part3 | 143.325 | 46.474 | 147.369 | 54.779 | 0.974 | 0.673 |
| Betweenness centrality | Right.parsorbitalis_part1 | 139.102 | 34.828 | 140.242 | 34.679 | 0.246 | 0.92 |
| Betweenness centrality | Right.parstriangularis_part1 | 138.548 | 27.569 | 136.263 | 27.557 | -0.946 | 0.674 |
| Betweenness centrality | Right.parstriangularis_part2 | 151.563 | 40.84 | 153.465 | 37.695 | 0.465 | 0.848 |
| Betweenness centrality | Right.parstriangularis_part3 | 171.244 | 40.186 | 167.407 | 47.129 | -0.877 | 0.696 |
| Betweenness centrality | Right.pericalcarine_part1 | 152.849 | 40.52 | 151.056 | 33.744 | -0.653 | 0.785 |
| Betweenness centrality | Right.pericalcarine_part2 | 146.612 | 36.176 | 145.788 | 36.255 | -0.121 | 0.952 |
| Betweenness centrality | Right.pericalcarine_part3 | 155.514 | 43.919 | 158.259 | 45.482 | 0.598 | 0.8 |
| Betweenness centrality | Right.postcentral_part1 | 142.338 | 41.537 | 148.381 | 42.139 | 1.442 | 0.525 |
| Betweenness centrality | Right.postcentral_part2 | 162.308 | 53.013 | 165.206 | 52.37 | 0.57 | 0.814 |
| Betweenness centrality | Right.postcentral_part3 | 122.811 | 40.838 | 123.554 | 39.057 | 0.151 | 0.941 |
| Betweenness centrality | Right.postcentral_part4 | 138.656 | 53.39 | 133.729 | 43.287 | -0.906 | 0.695 |
| Betweenness centrality | Right.postcentral_part5 | 118.465 | 45.67 | 127.794 | 44.749 | 2.084 | 0.335 |
| Betweenness centrality | Right.postcentral_part6 | 177.42 | 61.491 | 173.878 | 51.432 | -0.469 | 0.847 |
| Betweenness centrality | Right.postcentral_part7 | 168.62 | 47.43 | 166.314 | 49.823 | -0.554 | 0.818 |
| Betweenness centrality | Right.postcentral_part8 | 153.159 | 38.926 | 149.742 | 42.097 | -0.947 | 0.674 |
| Betweenness centrality | Right.posteriorcingulate_part1 | 162.308 | 46.289 | 162.516 | 46.241 | 0.167 | 0.941 |
| Betweenness centrality | Right.posteriorcingulate_part2 | 139.473 | 39.511 | 138.973 | 40.821 | -0.097 | 0.963 |
| Betweenness centrality | Right.precentral_part1 | 160.115 | 57.375 | 158.77 | 48.537 | -0.345 | 0.89 |
| Betweenness centrality | Right.precentral_part2 | 139.506 | 37.732 | 135.761 | 37.992 | -0.963 | 0.673 |
| Betweenness centrality | Right.precentral_part3 | 146.22 | 41.786 | 148.131 | 43.352 | 0.436 | 0.854 |
| Betweenness centrality | Right.precentral_part4 | 142.453 | 44.04 | 140.175 | 42.964 | -0.516 | 0.828 |
| Betweenness centrality | Right.precentral_part5 | 107.012 | 30.693 | 107.735 | 28.962 | 0.324 | 0.898 |
| Betweenness centrality | Right.precentral_part6 | 164.42 | 45.293 | 156.42 | 36.389 | -2.058 | 0.344 |
| Betweenness centrality | Right.precentral_part7 | 124.807 | 40.717 | 121.742 | 38.18 | -0.529 | 0.825 |
| Betweenness centrality | Right.precentral_part8 | 155.025 | 48.125 | 155.112 | 38.676 | 0.048 | 0.984 |
| Betweenness centrality | Right.precentral_part9 | 136.96 | 41.615 | 133.985 | 39.324 | -0.838 | 0.705 |
| Betweenness centrality | Right.precuneus_part1 | 143.598 | 41.225 | 141.638 | 33.879 | -0.617 | 0.796 |
| Betweenness centrality | Right.precuneus_part2 | 106.509 | 38.56 | 113.345 | 38.506 | 1.802 | 0.424 |
| Betweenness centrality | Right.precuneus_part3 | 137.577 | 42.211 | 133.066 | 39.481 | -1.075 | 0.644 |
| Betweenness centrality | Right.precuneus_part4 | 132.024 | 39.067 | 136.875 | 43.681 | 1.23 | 0.591 |
| Betweenness centrality | Right.precuneus_part5 | 135.734 | 35.218 | 131.294 | 40.297 | -1.194 | 0.602 |
| Betweenness centrality | Right.precuneus_part6 | 131.728 | 47.722 | 136.485 | 47.714 | 1.016 | 0.66 |
| Betweenness centrality | Right.precuneus_part7 | 158.653 | 45.548 | 159.846 | 43.903 | 0.396 | 0.875 |
| Betweenness centrality | Right.rostralanteriorcingulate_part1 | 170.413 | 46.342 | 162.43 | 40.019 | -1.646 | 0.457 |
| Betweenness centrality | Right.rostralmiddlefrontal_part1 | 151.858 | 49.251 | 147.398 | 44.733 | -0.871 | 0.697 |
| Betweenness centrality | Right.rostralmiddlefrontal_part2 | 158.87 | 49.868 | 162.486 | 44.901 | 0.718 | 0.751 |
| Betweenness centrality | Right.rostralmiddlefrontal_part3 | 154.886 | 47.191 | 156.158 | 45.265 | 0.243 | 0.92 |
| Betweenness centrality | Right.rostralmiddlefrontal_part4 | 157.509 | 41.498 | 163.069 | 44.34 | 1.586 | 0.472 |
| Betweenness centrality | Right.rostralmiddlefrontal_part5 | 138.575 | 42.213 | 139.14 | 41.844 | 0.312 | 0.902 |
| Betweenness centrality | Right.rostralmiddlefrontal_part6 | 138.597 | 34.822 | 136.533 | 34.376 | -0.867 | 0.697 |
| Betweenness centrality | Right.rostralmiddlefrontal_part7 | 153.225 | 48.368 | 154.964 | 49.121 | 0.322 | 0.898 |
| Betweenness centrality | Right.rostralmiddlefrontal_part8 | 139.222 | 42.571 | 148.567 | 44.654 | 2.017 | 0.348 |
| Betweenness centrality | Right.rostralmiddlefrontal_part9 | 154.799 | 51.227 | 148.739 | 43.695 | -1.396 | 0.537 |
| Betweenness centrality | Right.rostralmiddlefrontal_part10 | 141.875 | 40.965 | 134.593 | 37.222 | -1.785 | 0.424 |
| Betweenness centrality | Right.superiorfrontal_part1 | 162.427 | 56.412 | 158.06 | 47.622 | -0.871 | 0.697 |
| Betweenness centrality | Right.superiorfrontal_part2 | 145.522 | 49.698 | 150.836 | 60.658 | 0.965 | 0.673 |
| Betweenness centrality | Right.superiorfrontal_part3 | 160.352 | 48.398 | 167.435 | 57.625 | 1.259 | 0.589 |
| Betweenness centrality | Right.superiorfrontal_part4 | 154.048 | 51.895 | 155.008 | 50.214 | 0.329 | 0.897 |
| Betweenness centrality | Right.superiorfrontal_part5 | 137.317 | 40.458 | 135.903 | 39.398 | -0.305 | 0.906 |
| Betweenness centrality | Right.superiorfrontal_part6 | 129.098 | 36.45 | 127.8 | 37.8 | -0.271 | 0.914 |
| Betweenness centrality | Right.superiorfrontal_part7 | 139.373 | 39.244 | 139.453 | 43.55 | -0.159 | 0.941 |
| Betweenness centrality | Right.superiorfrontal_part8 | 133.92 | 37.388 | 135.338 | 39.251 | 0.355 | 0.885 |
| Betweenness centrality | Right.superiorfrontal_part9 | 143.447 | 44.904 | 139.142 | 44.131 | -0.939 | 0.678 |
| Betweenness centrality | Right.superiorfrontal_part10 | 129.336 | 36.887 | 132.33 | 34.693 | 0.766 | 0.732 |
| Betweenness centrality | Right.superiorfrontal_part11 | 134.425 | 41.06 | 131.255 | 42.098 | -0.787 | 0.728 |
| Betweenness centrality | Right.superiorfrontal_part12 | 148.002 | 45.737 | 141.466 | 46.843 | -1.493 | 0.517 |
| Betweenness centrality | Right.superiorfrontal_part13 | 138.914 | 43.81 | 140.089 | 48.147 | 0.345 | 0.89 |
| Betweenness centrality | Right.superiorparietal_part1 | 143.809 | 35.716 | 139.581 | 31.766 | -1.145 | 0.626 |
| Betweenness centrality | Right.superiorparietal_part2 | 140.839 | 43.52 | 139.269 | 47.709 | -0.237 | 0.92 |
| Betweenness centrality | Right.superiorparietal_part3 | 126.791 | 32.979 | 134.809 | 35.662 | 2.543 | 0.212 |
| Betweenness centrality | Right.superiorparietal_part4 | 134.173 | 41.99 | 138.36 | 46.675 | 1.103 | 0.634 |
| Betweenness centrality | Right.superiorparietal_part5 | 150.857 | 41.714 | 143.493 | 40.945 | -1.772 | 0.424 |
| Betweenness centrality | Right.superiorparietal_part6 | 174.361 | 53.545 | 172.966 | 49.668 | -0.17 | 0.941 |
| Betweenness centrality | Right.superiorparietal_part7 | 170.596 | 47.055 | 171.634 | 46.386 | 0.353 | 0.885 |
| Betweenness centrality | Right.superiorparietal_part8 | 168.08 | 48.377 | 173.275 | 57.213 | 1.186 | 0.603 |
| Betweenness centrality | Right.superiorparietal_part9 | 149.69 | 50.926 | 147.736 | 48.239 | -0.532 | 0.824 |
| Betweenness centrality | Right.superiorparietal_part10 | 151.175 | 51.367 | 157.519 | 51.51 | 1.456 | 0.522 |
| Betweenness centrality | Right.superiortemporal_part1 | 169.614 | 45.057 | 167.823 | 45.73 | -0.363 | 0.88 |
| Betweenness centrality | Right.superiortemporal_part2 | 174.442 | 53.31 | 172.142 | 52.589 | -0.447 | 0.851 |
| Betweenness centrality | Right.superiortemporal_part3 | 146.074 | 42.942 | 155.919 | 50.381 | 2.188 | 0.313 |
| Betweenness centrality | Right.superiortemporal_part4 | 178.557 | 44.31 | 180.783 | 47.152 | 0.56 | 0.817 |
| Betweenness centrality | Right.superiortemporal_part5 | 157.933 | 44.549 | 153.16 | 41.676 | -0.966 | 0.673 |
| Betweenness centrality | Right.superiortemporal_part6 | 150.591 | 39.158 | 146.388 | 39.463 | -0.96 | 0.673 |
| Betweenness centrality | Right.supramarginal_part1 | 169.953 | 46.502 | 161.97 | 45.243 | -1.719 | 0.439 |
| Betweenness centrality | Right.supramarginal_part2 | 171.031 | 46.961 | 162.376 | 44.081 | -1.869 | 0.407 |
| Betweenness centrality | Right.supramarginal_part3 | 172.098 | 42.43 | 169.337 | 51.136 | -0.637 | 0.786 |
| Betweenness centrality | Right.supramarginal_part4 | 168.873 | 43.718 | 166.869 | 45.247 | -0.314 | 0.902 |
| Betweenness centrality | Right.supramarginal_part5 | 170.471 | 34.312 | 165.888 | 35.366 | -1.281 | 0.575 |
| Betweenness centrality | Right.supramarginal_part6 | 179.765 | 48.38 | 170.138 | 45.672 | -2.248 | 0.291 |
| Betweenness centrality | Right.supramarginal_part7 | 158.7 | 49.504 | 146.669 | 46.29 | -2.724 | 0.188 |
| Betweenness centrality | Right.frontalpole_part1 | 156.586 | 43.295 | 157.118 | 45.742 | 0.169 | 0.941 |
| Betweenness centrality | Right.temporalpole_part1 | 167.714 | 41.289 | 166.413 | 41.723 | -0.276 | 0.914 |
| Betweenness centrality | Right.transversetemporal_part1 | 166.525 | 47.856 | 162.234 | 41.487 | -1.018 | 0.66 |
| Betweenness centrality | Right.insula_part1 | 162.804 | 51.358 | 160.555 | 48.263 | -0.372 | 0.878 |
| Betweenness centrality | Right.insula_part2 | 160.178 | 45.248 | 157.035 | 43.207 | -0.973 | 0.673 |
| Betweenness centrality | Right.insula_part3 | 136.129 | 40.333 | 140.414 | 35.618 | 1.049 | 0.654 |
| Betweenness centrality | Right.insula_part4 | 200.571 | 55.762 | 197.967 | 50.037 | -0.502 | 0.831 |
| Eigenvector centrality | Left.bankssts_part1 | 0.637 | 0.222 | 0.666 | 0.221 | 1.396 | 0.537 |
| Eigenvector centrality | Left.bankssts_part2 | 0.692 | 0.214 | 0.719 | 0.209 | 1.598 | 0.469 |
| Eigenvector centrality | Left.caudalanteriorcingulate_part1 | 0.378 | 0.145 | 0.412 | 0.169 | 2.227 | 0.296 |
| Eigenvector centrality | Left.caudalmiddlefrontal_part1 | 0.671 | 0.181 | 0.668 | 0.173 | -0.092 | 0.964 |
| Eigenvector centrality | Left.caudalmiddlefrontal_part2 | 0.699 | 0.179 | 0.667 | 0.178 | -1.662 | 0.457 |
| Eigenvector centrality | Left.caudalmiddlefrontal_part3 | 0.577 | 0.169 | 0.6 | 0.154 | 1.491 | 0.517 |
| Eigenvector centrality | Left.caudalmiddlefrontal_part4 | 0.677 | 0.19 | 0.684 | 0.185 | 0.392 | 0.876 |
| Eigenvector centrality | Left.cuneus_part1 | 0.335 | 0.104 | 0.353 | 0.124 | 1.65 | 0.457 |
| Eigenvector centrality | Left.cuneus_part2 | 0.313 | 0.097 | 0.331 | 0.117 | 1.684 | 0.455 |
| Eigenvector centrality | Left.entorhinal_part1 | 0.217 | 0.076 | 0.232 | 0.083 | 1.78 | 0.424 |
| Eigenvector centrality | Left.fusiform_part1 | 0.305 | 0.086 | 0.328 | 0.104 | 2.36 | 0.259 |
| Eigenvector centrality | Left.fusiform_part2 | 0.506 | 0.177 | 0.537 | 0.185 | 1.764 | 0.425 |
| Eigenvector centrality | Left.fusiform_part3 | 0.399 | 0.139 | 0.412 | 0.147 | 1.034 | 0.657 |
| Eigenvector centrality | Left.fusiform_part4 | 0.332 | 0.097 | 0.35 | 0.116 | 1.744 | 0.429 |
| Eigenvector centrality | Left.fusiform_part5 | 0.397 | 0.139 | 0.418 | 0.148 | 1.495 | 0.517 |
| Eigenvector centrality | Left.inferiorparietal_part1 | 0.431 | 0.153 | 0.435 | 0.154 | 0.296 | 0.907 |
| Eigenvector centrality | Left.inferiorparietal_part2 | 0.398 | 0.141 | 0.399 | 0.158 | -0.002 | 1 |
| Eigenvector centrality | Left.inferiorparietal_part3 | 0.306 | 0.099 | 0.326 | 0.118 | 1.791 | 0.424 |
| Eigenvector centrality | Left.inferiorparietal_part4 | 0.541 | 0.193 | 0.56 | 0.201 | 0.99 | 0.673 |
| Eigenvector centrality | Left.inferiorparietal_part5 | 0.388 | 0.162 | 0.395 | 0.157 | 0.234 | 0.92 |
| Eigenvector centrality | Left.inferiorparietal_part6 | 0.391 | 0.127 | 0.417 | 0.142 | 2.093 | 0.33 |
| Eigenvector centrality | Left.inferiorparietal_part7 | 0.329 | 0.119 | 0.339 | 0.124 | 0.787 | 0.728 |
| Eigenvector centrality | Left.inferiorparietal_part8 | 0.32 | 0.111 | 0.335 | 0.138 | 1.116 | 0.631 |
| Eigenvector centrality | Left.inferiortemporal_part1 | 0.446 | 0.187 | 0.446 | 0.177 | -0.027 | 0.987 |
| Eigenvector centrality | Left.inferiortemporal_part2 | 0.395 | 0.147 | 0.424 | 0.173 | 1.724 | 0.437 |
| Eigenvector centrality | Left.inferiortemporal_part3 | 0.412 | 0.164 | 0.389 | 0.16 | -1.436 | 0.525 |
| Eigenvector centrality | Left.inferiortemporal_part4 | 0.363 | 0.154 | 0.356 | 0.132 | -0.355 | 0.885 |
| Eigenvector centrality | Left.inferiortemporal_part5 | 0.433 | 0.184 | 0.417 | 0.169 | -0.944 | 0.675 |
| Eigenvector centrality | Left.inferiortemporal_part6 | 0.336 | 0.13 | 0.354 | 0.143 | 1.337 | 0.56 |
| Eigenvector centrality | Left.isthmuscingulate_part1 | 0.355 | 0.138 | 0.363 | 0.135 | 0.736 | 0.742 |
| Eigenvector centrality | Left.isthmuscingulate_part2 | 0.567 | 0.188 | 0.595 | 0.199 | 1.413 | 0.533 |
| Eigenvector centrality | Left.lateraloccipital_part1 | 0.612 | 0.163 | 0.625 | 0.154 | 0.848 | 0.705 |
| Eigenvector centrality | Left.lateraloccipital_part2 | 0.512 | 0.207 | 0.492 | 0.201 | -1.155 | 0.619 |
| Eigenvector centrality | Left.lateraloccipital_part3 | 0.72 | 0.171 | 0.729 | 0.177 | 0.555 | 0.818 |
| Eigenvector centrality | Left.lateraloccipital_part4 | 0.421 | 0.144 | 0.464 | 0.157 | 2.962 | 0.159 |
| Eigenvector centrality | Left.lateraloccipital_part5 | 0.32 | 0.114 | 0.348 | 0.128 | 2.491 | 0.219 |
| Eigenvector centrality | Left.lateraloccipital_part6 | 0.324 | 0.109 | 0.33 | 0.122 | 0.499 | 0.832 |
| Eigenvector centrality | Left.lateraloccipital_part7 | 0.344 | 0.118 | 0.345 | 0.127 | 0.202 | 0.932 |
| Eigenvector centrality | Left.lateraloccipital_part8 | 0.297 | 0.094 | 0.311 | 0.103 | 1.323 | 0.567 |
| Eigenvector centrality | Left.lateraloccipital_part9 | 0.31 | 0.091 | 0.319 | 0.118 | 0.87 | 0.697 |
| Eigenvector centrality | Left.lateralorbitofrontal_part1 | 0.285 | 0.092 | 0.299 | 0.101 | 1.428 | 0.527 |
| Eigenvector centrality | Left.lateralorbitofrontal_part2 | 0.285 | 0.089 | 0.298 | 0.101 | 1.588 | 0.472 |
| Eigenvector centrality | Left.lateralorbitofrontal_part3 | 0.333 | 0.104 | 0.346 | 0.12 | 1.162 | 0.618 |
| Eigenvector centrality | Left.lateralorbitofrontal_part4 | 0.284 | 0.084 | 0.295 | 0.096 | 1.277 | 0.577 |
| Eigenvector centrality | Left.lingual_part1 | 0.291 | 0.092 | 0.301 | 0.099 | 1.133 | 0.63 |
| Eigenvector centrality | Left.lingual_part2 | 0.298 | 0.092 | 0.321 | 0.113 | 2.314 | 0.26 |
| Eigenvector centrality | Left.lingual_part3 | 0.299 | 0.083 | 0.327 | 0.112 | 3.051 | 0.136 |
| Eigenvector centrality | Left.lingual_part4 | 0.346 | 0.112 | 0.355 | 0.123 | 0.783 | 0.729 |
| Eigenvector centrality | Left.lingual_part5 | 0.454 | 0.162 | 0.464 | 0.17 | 0.647 | 0.786 |
| Eigenvector centrality | Left.lingual_part6 | 0.356 | 0.125 | 0.381 | 0.139 | 2.03 | 0.348 |
| Eigenvector centrality | Left.medialorbitofrontal_part1 | 0.299 | 0.096 | 0.313 | 0.108 | 1.367 | 0.546 |
| Eigenvector centrality | Left.medialorbitofrontal_part2 | 0.391 | 0.153 | 0.386 | 0.14 | -0.431 | 0.857 |
| Eigenvector centrality | Left.medialorbitofrontal_part3 | 0.315 | 0.102 | 0.33 | 0.119 | 1.329 | 0.565 |
| Eigenvector centrality | Left.middletemporal_part1 | 0.358 | 0.128 | 0.365 | 0.125 | 0.435 | 0.854 |
| Eigenvector centrality | Left.middletemporal_part2 | 0.31 | 0.097 | 0.324 | 0.107 | 1.373 | 0.546 |
| Eigenvector centrality | Left.middletemporal_part3 | 0.299 | 0.135 | 0.338 | 0.157 | 2.57 | 0.209 |
| Eigenvector centrality | Left.middletemporal_part4 | 0.329 | 0.117 | 0.354 | 0.135 | 2.1 | 0.328 |
| Eigenvector centrality | Left.middletemporal_part5 | 0.291 | 0.099 | 0.316 | 0.12 | 2.445 | 0.232 |
| Eigenvector centrality | Left.parahippocampal_part1 | 0.439 | 0.139 | 0.458 | 0.16 | 1.161 | 0.618 |
| Eigenvector centrality | Left.parahippocampal_part2 | 0.343 | 0.109 | 0.363 | 0.131 | 1.698 | 0.45 |
| Eigenvector centrality | Left.paracentral_part1 | 0.526 | 0.181 | 0.559 | 0.178 | 1.851 | 0.413 |
| Eigenvector centrality | Left.paracentral_part2 | 0.391 | 0.118 | 0.406 | 0.14 | 1.216 | 0.591 |
| Eigenvector centrality | Left.paracentral_part3 | 0.507 | 0.174 | 0.521 | 0.194 | 0.819 | 0.712 |
| Eigenvector centrality | Left.parsopercularis_part1 | 0.69 | 0.165 | 0.654 | 0.174 | -2.139 | 0.32 |
| Eigenvector centrality | Left.parsopercularis_part2 | 0.798 | 0.126 | 0.755 | 0.172 | -2.836 | 0.184 |
| Eigenvector centrality | Left.parsopercularis_part3 | 0.753 | 0.18 | 0.675 | 0.198 | -4.144 | 0.01 |
| Eigenvector centrality | Left.parsorbitalis_part1 | 0.698 | 0.147 | 0.711 | 0.151 | 1.065 | 0.651 |
| Eigenvector centrality | Left.parstriangularis_part1 | 0.687 | 0.142 | 0.707 | 0.144 | 1.3 | 0.57 |
| Eigenvector centrality | Left.parstriangularis_part2 | 0.53 | 0.199 | 0.537 | 0.196 | 0.324 | 0.898 |
| Eigenvector centrality | Left.pericalcarine_part1 | 0.686 | 0.168 | 0.68 | 0.176 | -0.322 | 0.898 |
| Eigenvector centrality | Left.pericalcarine_part2 | 0.775 | 0.167 | 0.755 | 0.162 | -1.148 | 0.625 |
| Eigenvector centrality | Left.postcentral_part1 | 0.33 | 0.092 | 0.348 | 0.113 | 1.786 | 0.424 |
| Eigenvector centrality | Left.postcentral_part2 | 0.412 | 0.144 | 0.43 | 0.167 | 1.235 | 0.591 |
| Eigenvector centrality | Left.postcentral_part3 | 0.494 | 0.165 | 0.509 | 0.185 | 1.065 | 0.651 |
| Eigenvector centrality | Left.postcentral_part4 | 0.319 | 0.111 | 0.329 | 0.114 | 0.876 | 0.696 |
| Eigenvector centrality | Left.postcentral_part5 | 0.307 | 0.095 | 0.328 | 0.119 | 1.98 | 0.36 |
| Eigenvector centrality | Left.postcentral_part6 | 0.49 | 0.142 | 0.449 | 0.139 | -3.229 | 0.084 |
| Eigenvector centrality | Left.postcentral_part7 | 0.415 | 0.139 | 0.465 | 0.163 | 3.257 | 0.084 |
| Eigenvector centrality | Left.postcentral_part8 | 0.591 | 0.152 | 0.577 | 0.156 | -0.894 | 0.696 |
| Eigenvector centrality | Left.posteriorcingulate_part1 | 0.367 | 0.155 | 0.398 | 0.164 | 1.991 | 0.36 |
| Eigenvector centrality | Left.posteriorcingulate_part2 | 0.507 | 0.17 | 0.488 | 0.181 | -1.208 | 0.596 |
| Eigenvector centrality | Left.precentral_part1 | 0.451 | 0.156 | 0.471 | 0.145 | 1.251 | 0.59 |
| Eigenvector centrality | Left.precentral_part2 | 0.58 | 0.19 | 0.607 | 0.19 | 1.482 | 0.521 |
| Eigenvector centrality | Left.precentral_part3 | 0.442 | 0.15 | 0.466 | 0.174 | 1.455 | 0.522 |
| Eigenvector centrality | Left.precentral_part4 | 0.438 | 0.168 | 0.464 | 0.186 | 1.66 | 0.457 |
| Eigenvector centrality | Left.precentral_part5 | 0.306 | 0.107 | 0.305 | 0.1 | 0.086 | 0.966 |
| Eigenvector centrality | Left.precentral_part6 | 0.407 | 0.199 | 0.424 | 0.201 | 1.033 | 0.657 |
| Eigenvector centrality | Left.precentral_part7 | 0.725 | 0.156 | 0.706 | 0.179 | -1.143 | 0.627 |
| Eigenvector centrality | Left.precentral_part8 | 0.673 | 0.208 | 0.603 | 0.222 | -3.303 | 0.08 |
| Eigenvector centrality | Left.precentral_part9 | 0.659 | 0.186 | 0.688 | 0.18 | 1.57 | 0.483 |
| Eigenvector centrality | Left.precuneus_part1 | 0.751 | 0.199 | 0.701 | 0.2 | -2.353 | 0.259 |
| Eigenvector centrality | Left.precuneus_part2 | 0.716 | 0.224 | 0.695 | 0.229 | -0.687 | 0.765 |
| Eigenvector centrality | Left.precuneus_part3 | 0.771 | 0.179 | 0.752 | 0.193 | -0.858 | 0.702 |
| Eigenvector centrality | Left.precuneus_part4 | 0.751 | 0.18 | 0.711 | 0.178 | -2.311 | 0.26 |
| Eigenvector centrality | Left.precuneus_part5 | 0.672 | 0.226 | 0.688 | 0.229 | 0.948 | 0.674 |
| Eigenvector centrality | Left.precuneus_part6 | 0.369 | 0.148 | 0.374 | 0.157 | 0.306 | 0.906 |
| Eigenvector centrality | Left.precuneus_part7 | 0.611 | 0.178 | 0.608 | 0.183 | -0.239 | 0.92 |
| Eigenvector centrality | Left.rostralanteriorcingulate_part1 | 0.501 | 0.186 | 0.55 | 0.192 | 2.657 | 0.188 |
| Eigenvector centrality | Left.rostralmiddlefrontal_part1 | 0.436 | 0.164 | 0.452 | 0.174 | 0.971 | 0.673 |
| Eigenvector centrality | Left.rostralmiddlefrontal_part2 | 0.465 | 0.163 | 0.469 | 0.152 | 0.235 | 0.92 |
| Eigenvector centrality | Left.rostralmiddlefrontal_part3 | 0.441 | 0.158 | 0.46 | 0.17 | 1.078 | 0.644 |
| Eigenvector centrality | Left.rostralmiddlefrontal_part4 | 0.467 | 0.195 | 0.476 | 0.193 | 0.452 | 0.85 |
| Eigenvector centrality | Left.rostralmiddlefrontal_part5 | 0.385 | 0.112 | 0.403 | 0.133 | 1.547 | 0.493 |
| Eigenvector centrality | Left.rostralmiddlefrontal_part6 | 0.38 | 0.133 | 0.405 | 0.152 | 1.776 | 0.424 |
| Eigenvector centrality | Left.rostralmiddlefrontal_part7 | 0.384 | 0.137 | 0.395 | 0.135 | 0.638 | 0.786 |
| Eigenvector centrality | Left.rostralmiddlefrontal_part8 | 0.325 | 0.118 | 0.334 | 0.117 | 0.852 | 0.705 |
| Eigenvector centrality | Left.rostralmiddlefrontal_part9 | 0.479 | 0.19 | 0.494 | 0.197 | 0.865 | 0.698 |
| Eigenvector centrality | Left.rostralmiddlefrontal_part10 | 0.288 | 0.093 | 0.306 | 0.105 | 1.867 | 0.407 |
| Eigenvector centrality | Left.superiorfrontal_part1 | 0.467 | 0.183 | 0.472 | 0.179 | 0.277 | 0.914 |
| Eigenvector centrality | Left.superiorfrontal_part2 | 0.308 | 0.09 | 0.331 | 0.117 | 2.343 | 0.259 |
| Eigenvector centrality | Left.superiorfrontal_part3 | 0.466 | 0.173 | 0.495 | 0.189 | 1.735 | 0.434 |
| Eigenvector centrality | Left.superiorfrontal_part4 | 0.339 | 0.114 | 0.361 | 0.138 | 1.745 | 0.429 |
| Eigenvector centrality | Left.superiorfrontal_part5 | 0.337 | 0.113 | 0.364 | 0.132 | 2.32 | 0.26 |
| Eigenvector centrality | Left.superiorfrontal_part6 | 0.318 | 0.098 | 0.34 | 0.114 | 2.201 | 0.309 |
| Eigenvector centrality | Left.superiorfrontal_part7 | 0.735 | 0.177 | 0.743 | 0.174 | 0.596 | 0.8 |
| Eigenvector centrality | Left.superiorfrontal_part8 | 0.72 | 0.186 | 0.723 | 0.182 | 0.247 | 0.92 |
| Eigenvector centrality | Left.superiorfrontal_part9 | 0.726 | 0.19 | 0.71 | 0.182 | -0.809 | 0.714 |
| Eigenvector centrality | Left.superiorfrontal_part10 | 0.713 | 0.161 | 0.709 | 0.171 | -0.144 | 0.942 |
| Eigenvector centrality | Left.superiorfrontal_part11 | 0.762 | 0.171 | 0.698 | 0.185 | -3.611 | 0.042 |
| Eigenvector centrality | Left.superiorfrontal_part12 | 0.447 | 0.186 | 0.47 | 0.186 | 1.211 | 0.593 |
| Eigenvector centrality | Left.superiorfrontal_part13 | 0.781 | 0.19 | 0.76 | 0.171 | -1.222 | 0.591 |
| Eigenvector centrality | Left.superiorparietal_part1 | 0.476 | 0.208 | 0.502 | 0.197 | 1.432 | 0.525 |
| Eigenvector centrality | Left.superiorparietal_part2 | 0.788 | 0.142 | 0.76 | 0.176 | -1.621 | 0.469 |
| Eigenvector centrality | Left.superiorparietal_part3 | 0.694 | 0.165 | 0.678 | 0.181 | -0.746 | 0.737 |
| Eigenvector centrality | Left.superiorparietal_part4 | 0.708 | 0.194 | 0.687 | 0.19 | -0.983 | 0.673 |
| Eigenvector centrality | Left.superiorparietal_part5 | 0.658 | 0.211 | 0.643 | 0.199 | -0.664 | 0.777 |
| Eigenvector centrality | Left.superiorparietal_part6 | 0.51 | 0.165 | 0.489 | 0.164 | -1.583 | 0.472 |
| Eigenvector centrality | Left.superiorparietal_part7 | 0.415 | 0.162 | 0.396 | 0.154 | -1.584 | 0.472 |
| Eigenvector centrality | Left.superiorparietal_part8 | 0.352 | 0.13 | 0.352 | 0.134 | -0.052 | 0.983 |
| Eigenvector centrality | Left.superiorparietal_part9 | 0.474 | 0.168 | 0.455 | 0.158 | -1.462 | 0.522 |
| Eigenvector centrality | Left.superiorparietal_part10 | 0.537 | 0.166 | 0.554 | 0.163 | 1.007 | 0.667 |
| Eigenvector centrality | Left.superiortemporal_part1 | 0.455 | 0.184 | 0.35 | 0.156 | -6.713 | <0.001 |
| Eigenvector centrality | Left.superiortemporal_part2 | 0.43 | 0.173 | 0.414 | 0.165 | -1.084 | 0.644 |
| Eigenvector centrality | Left.superiortemporal_part3 | 0.487 | 0.184 | 0.44 | 0.171 | -2.907 | 0.163 |
| Eigenvector centrality | Left.superiortemporal_part4 | 0.599 | 0.18 | 0.597 | 0.186 | -0.231 | 0.92 |
| Eigenvector centrality | Left.superiortemporal_part5 | 0.613 | 0.181 | 0.585 | 0.175 | -1.672 | 0.457 |
| Eigenvector centrality | Left.superiortemporal_part6 | 0.758 | 0.162 | 0.769 | 0.162 | 0.961 | 0.673 |
| Eigenvector centrality | Left.superiortemporal_part7 | 0.748 | 0.134 | 0.762 | 0.124 | 1.188 | 0.603 |
| Eigenvector centrality | Left.supramarginal_part1 | 0.444 | 0.166 | 0.434 | 0.14 | -0.836 | 0.705 |
| Eigenvector centrality | Left.supramarginal_part2 | 0.703 | 0.159 | 0.727 | 0.163 | 1.593 | 0.471 |
| Eigenvector centrality | Left.supramarginal_part3 | 0.656 | 0.207 | 0.665 | 0.198 | 0.602 | 0.8 |
| Eigenvector centrality | Left.supramarginal_part4 | 0.687 | 0.197 | 0.687 | 0.213 | 0.044 | 0.985 |
| Eigenvector centrality | Left.supramarginal_part5 | 0.849 | 0.135 | 0.873 | 0.106 | 1.927 | 0.382 |
| Eigenvector centrality | Left.supramarginal_part6 | 0.477 | 0.177 | 0.476 | 0.176 | -0.146 | 0.942 |
| Eigenvector centrality | Left.supramarginal_part7 | 0.442 | 0.159 | 0.476 | 0.164 | 2.152 | 0.317 |
| Eigenvector centrality | Left.frontalpole_part1 | 0.384 | 0.146 | 0.381 | 0.14 | -0.346 | 0.89 |
| Eigenvector centrality | Left.temporalpole_part1 | 0.43 | 0.156 | 0.481 | 0.184 | 2.877 | 0.168 |
| Eigenvector centrality | Left.transversetemporal_part1 | 0.405 | 0.166 | 0.408 | 0.161 | 0.064 | 0.98 |
| Eigenvector centrality | Left.insula_part1 | 0.489 | 0.183 | 0.479 | 0.18 | -0.705 | 0.758 |
| Eigenvector centrality | Left.insula_part2 | 0.58 | 0.216 | 0.612 | 0.195 | 1.61 | 0.469 |
| Eigenvector centrality | Left.insula_part3 | 0.518 | 0.161 | 0.533 | 0.152 | 0.983 | 0.673 |
| Eigenvector centrality | Left.insula_part4 | 0.541 | 0.177 | 0.566 | 0.168 | 1.534 | 0.5 |
| Eigenvector centrality | Right.bankssts_part1 | 0.732 | 0.179 | 0.755 | 0.167 | 1.304 | 0.568 |
| Eigenvector centrality | Right.bankssts_part2 | 0.677 | 0.222 | 0.689 | 0.219 | 0.517 | 0.828 |
| Eigenvector centrality | Right.caudalanteriorcingulate_part1 | 0.341 | 0.111 | 0.371 | 0.136 | 2.624 | 0.191 |
| Eigenvector centrality | Right.caudalmiddlefrontal_part1 | 0.594 | 0.168 | 0.612 | 0.168 | 1.293 | 0.572 |
| Eigenvector centrality | Right.caudalmiddlefrontal_part2 | 0.71 | 0.199 | 0.689 | 0.19 | -1.234 | 0.591 |
| Eigenvector centrality | Right.caudalmiddlefrontal_part3 | 0.604 | 0.21 | 0.611 | 0.217 | 0.24 | 0.92 |
| Eigenvector centrality | Right.caudalmiddlefrontal_part4 | 0.748 | 0.183 | 0.714 | 0.173 | -2.073 | 0.339 |
| Eigenvector centrality | Right.cuneus_part1 | 0.372 | 0.123 | 0.382 | 0.13 | 0.671 | 0.774 |
| Eigenvector centrality | Right.cuneus_part2 | 0.305 | 0.101 | 0.311 | 0.099 | 0.534 | 0.824 |
| Eigenvector centrality | Right.cuneus_part3 | 0.33 | 0.106 | 0.343 | 0.122 | 1.055 | 0.653 |
| Eigenvector centrality | Right.entorhinal_part1 | 0.248 | 0.086 | 0.262 | 0.08 | 1.423 | 0.531 |
| Eigenvector centrality | Right.fusiform_part1 | 0.291 | 0.085 | 0.307 | 0.098 | 1.656 | 0.457 |
| Eigenvector centrality | Right.fusiform_part2 | 0.345 | 0.109 | 0.363 | 0.116 | 1.616 | 0.469 |
| Eigenvector centrality | Right.fusiform_part3 | 0.387 | 0.157 | 0.386 | 0.146 | -0.263 | 0.915 |
| Eigenvector centrality | Right.fusiform_part4 | 0.455 | 0.158 | 0.443 | 0.162 | -0.639 | 0.786 |
| Eigenvector centrality | Right.fusiform_part5 | 0.442 | 0.158 | 0.449 | 0.17 | 0.394 | 0.875 |
| Eigenvector centrality | Right.inferiorparietal_part1 | 0.497 | 0.177 | 0.519 | 0.178 | 1.171 | 0.616 |
| Eigenvector centrality | Right.inferiorparietal_part2 | 0.516 | 0.184 | 0.528 | 0.179 | 0.602 | 0.8 |
| Eigenvector centrality | Right.inferiorparietal_part3 | 0.39 | 0.168 | 0.437 | 0.189 | 2.528 | 0.212 |
| Eigenvector centrality | Right.inferiorparietal_part4 | 0.296 | 0.096 | 0.314 | 0.105 | 1.658 | 0.457 |
| Eigenvector centrality | Right.inferiorparietal_part5 | 0.481 | 0.191 | 0.498 | 0.198 | 0.877 | 0.696 |
| Eigenvector centrality | Right.inferiorparietal_part6 | 0.36 | 0.144 | 0.341 | 0.12 | -1.563 | 0.483 |
| Eigenvector centrality | Right.inferiorparietal_part7 | 0.404 | 0.156 | 0.407 | 0.15 | 0.245 | 0.92 |
| Eigenvector centrality | Right.inferiorparietal_part8 | 0.506 | 0.18 | 0.517 | 0.2 | 0.515 | 0.828 |
| Eigenvector centrality | Right.inferiorparietal_part9 | 0.326 | 0.119 | 0.336 | 0.129 | 0.745 | 0.737 |
| Eigenvector centrality | Right.inferiorparietal_part10 | 0.337 | 0.112 | 0.356 | 0.134 | 1.469 | 0.522 |
| Eigenvector centrality | Right.inferiortemporal_part1 | 0.363 | 0.142 | 0.368 | 0.149 | 0.109 | 0.96 |
| Eigenvector centrality | Right.inferiortemporal_part2 | 0.45 | 0.166 | 0.436 | 0.16 | -1.022 | 0.66 |
| Eigenvector centrality | Right.inferiortemporal_part3 | 0.392 | 0.137 | 0.388 | 0.138 | -0.317 | 0.9 |
| Eigenvector centrality | Right.inferiortemporal_part4 | 0.378 | 0.139 | 0.381 | 0.151 | 0.204 | 0.932 |
| Eigenvector centrality | Right.inferiortemporal_part5 | 0.377 | 0.128 | 0.395 | 0.132 | 1.431 | 0.525 |
| Eigenvector centrality | Right.isthmuscingulate_part1 | 0.328 | 0.103 | 0.345 | 0.117 | 1.546 | 0.493 |
| Eigenvector centrality | Right.isthmuscingulate_part2 | 0.687 | 0.132 | 0.705 | 0.127 | 1.598 | 0.469 |
| Eigenvector centrality | Right.lateraloccipital_part1 | 0.661 | 0.182 | 0.652 | 0.189 | -0.572 | 0.812 |
| Eigenvector centrality | Right.lateraloccipital_part2 | 0.603 | 0.177 | 0.623 | 0.182 | 1.079 | 0.644 |
| Eigenvector centrality | Right.lateraloccipital_part3 | 0.708 | 0.137 | 0.714 | 0.143 | 0.487 | 0.841 |
| Eigenvector centrality | Right.lateraloccipital_part4 | 0.469 | 0.144 | 0.484 | 0.157 | 0.969 | 0.673 |
| Eigenvector centrality | Right.lateraloccipital_part5 | 0.322 | 0.107 | 0.344 | 0.119 | 2.145 | 0.32 |
| Eigenvector centrality | Right.lateraloccipital_part6 | 0.276 | 0.079 | 0.288 | 0.094 | 1.48 | 0.521 |
| Eigenvector centrality | Right.lateraloccipital_part7 | 0.367 | 0.132 | 0.377 | 0.145 | 0.868 | 0.697 |
| Eigenvector centrality | Right.lateraloccipital_part8 | 0.285 | 0.084 | 0.301 | 0.1 | 1.716 | 0.44 |
| Eigenvector centrality | Right.lateraloccipital_part9 | 0.322 | 0.105 | 0.324 | 0.116 | 0.138 | 0.945 |
| Eigenvector centrality | Right.lateralorbitofrontal_part1 | 0.282 | 0.078 | 0.297 | 0.099 | 1.825 | 0.421 |
| Eigenvector centrality | Right.lateralorbitofrontal_part2 | 0.32 | 0.102 | 0.328 | 0.115 | 0.704 | 0.758 |
| Eigenvector centrality | Right.lateralorbitofrontal_part3 | 0.29 | 0.089 | 0.3 | 0.094 | 1.16 | 0.618 |
| Eigenvector centrality | Right.lateralorbitofrontal_part4 | 0.284 | 0.09 | 0.292 | 0.095 | 0.932 | 0.682 |
| Eigenvector centrality | Right.lingual_part1 | 0.285 | 0.09 | 0.291 | 0.094 | 0.641 | 0.786 |
| Eigenvector centrality | Right.lingual_part2 | 0.33 | 0.129 | 0.343 | 0.127 | 0.89 | 0.696 |
| Eigenvector centrality | Right.lingual_part3 | 0.282 | 0.085 | 0.302 | 0.114 | 2.028 | 0.348 |
| Eigenvector centrality | Right.lingual_part4 | 0.537 | 0.203 | 0.551 | 0.216 | 0.823 | 0.712 |
| Eigenvector centrality | Right.lingual_part5 | 0.281 | 0.082 | 0.298 | 0.097 | 1.983 | 0.36 |
| Eigenvector centrality | Right.lingual_part6 | 0.392 | 0.144 | 0.395 | 0.139 | 0.255 | 0.918 |
| Eigenvector centrality | Right.medialorbitofrontal_part1 | 0.292 | 0.091 | 0.307 | 0.103 | 1.683 | 0.455 |
| Eigenvector centrality | Right.medialorbitofrontal_part2 | 0.411 | 0.168 | 0.412 | 0.16 | 0.033 | 0.987 |
| Eigenvector centrality | Right.medialorbitofrontal_part3 | 0.308 | 0.101 | 0.324 | 0.116 | 1.485 | 0.521 |
| Eigenvector centrality | Right.middletemporal_part1 | 0.325 | 0.106 | 0.34 | 0.124 | 1.23 | 0.591 |
| Eigenvector centrality | Right.middletemporal_part2 | 0.306 | 0.097 | 0.326 | 0.113 | 2 | 0.357 |
| Eigenvector centrality | Right.middletemporal_part3 | 0.309 | 0.112 | 0.319 | 0.113 | 0.796 | 0.722 |
| Eigenvector centrality | Right.middletemporal_part4 | 0.276 | 0.115 | 0.305 | 0.132 | 2.324 | 0.26 |
| Eigenvector centrality | Right.middletemporal_part5 | 0.301 | 0.095 | 0.322 | 0.105 | 2.098 | 0.328 |
| Eigenvector centrality | Right.middletemporal_part6 | 0.544 | 0.17 | 0.553 | 0.169 | 0.548 | 0.818 |
| Eigenvector centrality | Right.parahippocampal_part1 | 0.535 | 0.177 | 0.542 | 0.157 | 0.366 | 0.879 |
| Eigenvector centrality | Right.parahippocampal_part2 | 0.559 | 0.191 | 0.558 | 0.191 | -0.188 | 0.935 |
| Eigenvector centrality | Right.paracentral_part1 | 0.401 | 0.149 | 0.411 | 0.159 | 0.552 | 0.818 |
| Eigenvector centrality | Right.paracentral_part2 | 0.361 | 0.111 | 0.372 | 0.125 | 0.835 | 0.706 |
| Eigenvector centrality | Right.paracentral_part3 | 0.599 | 0.185 | 0.608 | 0.198 | 0.528 | 0.825 |
| Eigenvector centrality | Right.parsopercularis_part1 | 0.674 | 0.172 | 0.681 | 0.164 | 0.371 | 0.878 |
| Eigenvector centrality | Right.parsopercularis_part2 | 0.69 | 0.199 | 0.605 | 0.219 | -3.988 | 0.014 |
| Eigenvector centrality | Right.parsopercularis_part3 | 0.73 | 0.17 | 0.675 | 0.204 | -2.99 | 0.151 |
| Eigenvector centrality | Right.parsorbitalis_part1 | 0.681 | 0.136 | 0.676 | 0.144 | -0.502 | 0.831 |
| Eigenvector centrality | Right.parstriangularis_part1 | 0.705 | 0.134 | 0.712 | 0.15 | 0.597 | 0.8 |
| Eigenvector centrality | Right.parstriangularis_part2 | 0.654 | 0.159 | 0.675 | 0.152 | 1.317 | 0.567 |
| Eigenvector centrality | Right.parstriangularis_part3 | 0.559 | 0.18 | 0.538 | 0.187 | -1.231 | 0.591 |
| Eigenvector centrality | Right.pericalcarine_part1 | 0.767 | 0.147 | 0.756 | 0.147 | -0.771 | 0.73 |
| Eigenvector centrality | Right.pericalcarine_part2 | 0.339 | 0.091 | 0.351 | 0.105 | 1.321 | 0.567 |
| Eigenvector centrality | Right.pericalcarine_part3 | 0.333 | 0.12 | 0.365 | 0.136 | 2.557 | 0.212 |
| Eigenvector centrality | Right.postcentral_part1 | 0.703 | 0.17 | 0.711 | 0.169 | 0.758 | 0.735 |
| Eigenvector centrality | Right.postcentral_part2 | 0.424 | 0.176 | 0.433 | 0.15 | 0.565 | 0.815 |
| Eigenvector centrality | Right.postcentral_part3 | 0.316 | 0.108 | 0.333 | 0.122 | 1.444 | 0.525 |
| Eigenvector centrality | Right.postcentral_part4 | 0.327 | 0.119 | 0.332 | 0.119 | 0.377 | 0.878 |
| Eigenvector centrality | Right.postcentral_part5 | 0.322 | 0.118 | 0.337 | 0.126 | 1.122 | 0.63 |
| Eigenvector centrality | Right.postcentral_part6 | 0.449 | 0.131 | 0.422 | 0.134 | -2.281 | 0.278 |
| Eigenvector centrality | Right.postcentral_part7 | 0.505 | 0.181 | 0.539 | 0.186 | 1.894 | 0.399 |
| Eigenvector centrality | Right.postcentral_part8 | 0.62 | 0.157 | 0.605 | 0.163 | -0.979 | 0.673 |
| Eigenvector centrality | Right.posteriorcingulate_part1 | 0.354 | 0.146 | 0.384 | 0.147 | 1.921 | 0.383 |
| Eigenvector centrality | Right.posteriorcingulate_part2 | 0.673 | 0.203 | 0.628 | 0.21 | -2.112 | 0.328 |
| Eigenvector centrality | Right.precentral_part1 | 0.471 | 0.168 | 0.458 | 0.173 | -1.017 | 0.66 |
| Eigenvector centrality | Right.precentral_part2 | 0.576 | 0.171 | 0.561 | 0.179 | -0.891 | 0.696 |
| Eigenvector centrality | Right.precentral_part3 | 0.567 | 0.163 | 0.566 | 0.176 | -0.085 | 0.966 |
| Eigenvector centrality | Right.precentral_part4 | 0.331 | 0.137 | 0.351 | 0.146 | 1.42 | 0.531 |
| Eigenvector centrality | Right.precentral_part5 | 0.289 | 0.105 | 0.316 | 0.109 | 2.806 | 0.188 |
| Eigenvector centrality | Right.precentral_part6 | 0.715 | 0.163 | 0.719 | 0.175 | 0.244 | 0.92 |
| Eigenvector centrality | Right.precentral_part7 | 0.706 | 0.206 | 0.641 | 0.214 | -3.009 | 0.149 |
| Eigenvector centrality | Right.precentral_part8 | 0.661 | 0.193 | 0.678 | 0.187 | 0.894 | 0.696 |
| Eigenvector centrality | Right.precentral_part9 | 0.754 | 0.185 | 0.71 | 0.201 | -2.271 | 0.28 |
| Eigenvector centrality | Right.precuneus_part1 | 0.76 | 0.185 | 0.76 | 0.183 | -0.111 | 0.959 |
| Eigenvector centrality | Right.precuneus_part2 | 0.449 | 0.223 | 0.462 | 0.218 | 0.622 | 0.795 |
| Eigenvector centrality | Right.precuneus_part3 | 0.748 | 0.204 | 0.744 | 0.194 | -0.381 | 0.878 |
| Eigenvector centrality | Right.precuneus_part4 | 0.552 | 0.187 | 0.557 | 0.192 | 0.29 | 0.91 |
| Eigenvector centrality | Right.precuneus_part5 | 0.762 | 0.171 | 0.725 | 0.195 | -1.944 | 0.378 |
| Eigenvector centrality | Right.precuneus_part6 | 0.367 | 0.155 | 0.377 | 0.16 | 0.566 | 0.815 |
| Eigenvector centrality | Right.precuneus_part7 | 0.466 | 0.181 | 0.486 | 0.192 | 1.232 | 0.591 |
| Eigenvector centrality | Right.rostralanteriorcingulate_part1 | 0.599 | 0.181 | 0.597 | 0.187 | -0.32 | 0.899 |
| Eigenvector centrality | Right.rostralmiddlefrontal_part1 | 0.45 | 0.168 | 0.46 | 0.171 | 0.769 | 0.731 |
| Eigenvector centrality | Right.rostralmiddlefrontal_part2 | 0.415 | 0.149 | 0.427 | 0.151 | 0.664 | 0.777 |
| Eigenvector centrality | Right.rostralmiddlefrontal_part3 | 0.461 | 0.167 | 0.463 | 0.171 | 0.053 | 0.983 |
| Eigenvector centrality | Right.rostralmiddlefrontal_part4 | 0.391 | 0.146 | 0.433 | 0.167 | 2.695 | 0.188 |
| Eigenvector centrality | Right.rostralmiddlefrontal_part5 | 0.369 | 0.148 | 0.407 | 0.165 | 2.391 | 0.252 |
| Eigenvector centrality | Right.rostralmiddlefrontal_part6 | 0.279 | 0.088 | 0.294 | 0.1 | 1.473 | 0.522 |
| Eigenvector centrality | Right.rostralmiddlefrontal_part7 | 0.366 | 0.14 | 0.406 | 0.148 | 2.77 | 0.188 |
| Eigenvector centrality | Right.rostralmiddlefrontal_part8 | 0.315 | 0.112 | 0.331 | 0.113 | 1.312 | 0.567 |
| Eigenvector centrality | Right.rostralmiddlefrontal_part9 | 0.464 | 0.178 | 0.477 | 0.175 | 0.751 | 0.736 |
| Eigenvector centrality | Right.rostralmiddlefrontal_part10 | 0.306 | 0.104 | 0.319 | 0.112 | 1.18 | 0.607 |
| Eigenvector centrality | Right.superiorfrontal_part1 | 0.454 | 0.168 | 0.455 | 0.17 | -0.033 | 0.987 |
| Eigenvector centrality | Right.superiorfrontal_part2 | 0.326 | 0.123 | 0.344 | 0.123 | 1.443 | 0.525 |
| Eigenvector centrality | Right.superiorfrontal_part3 | 0.404 | 0.154 | 0.435 | 0.158 | 1.943 | 0.378 |
| Eigenvector centrality | Right.superiorfrontal_part4 | 0.388 | 0.156 | 0.393 | 0.159 | 0.272 | 0.914 |
| Eigenvector centrality | Right.superiorfrontal_part5 | 0.312 | 0.099 | 0.335 | 0.121 | 2.105 | 0.328 |
| Eigenvector centrality | Right.superiorfrontal_part6 | 0.756 | 0.158 | 0.736 | 0.17 | -1.243 | 0.591 |
| Eigenvector centrality | Right.superiorfrontal_part7 | 0.7 | 0.172 | 0.717 | 0.162 | 1.081 | 0.644 |
| Eigenvector centrality | Right.superiorfrontal_part8 | 0.647 | 0.186 | 0.642 | 0.188 | -0.341 | 0.891 |
| Eigenvector centrality | Right.superiorfrontal_part9 | 0.636 | 0.212 | 0.64 | 0.202 | 0.272 | 0.914 |
| Eigenvector centrality | Right.superiorfrontal_part10 | 0.764 | 0.158 | 0.74 | 0.17 | -1.454 | 0.522 |
| Eigenvector centrality | Right.superiorfrontal_part11 | 0.309 | 0.107 | 0.318 | 0.104 | 0.901 | 0.696 |
| Eigenvector centrality | Right.superiorfrontal_part12 | 0.723 | 0.171 | 0.706 | 0.173 | -1.041 | 0.657 |
| Eigenvector centrality | Right.superiorfrontal_part13 | 0.432 | 0.193 | 0.454 | 0.187 | 1.288 | 0.575 |
| Eigenvector centrality | Right.superiorparietal_part1 | 0.794 | 0.158 | 0.779 | 0.158 | -0.976 | 0.673 |
| Eigenvector centrality | Right.superiorparietal_part2 | 0.463 | 0.195 | 0.485 | 0.2 | 1.304 | 0.568 |
| Eigenvector centrality | Right.superiorparietal_part3 | 0.736 | 0.172 | 0.71 | 0.176 | -1.404 | 0.536 |
| Eigenvector centrality | Right.superiorparietal_part4 | 0.604 | 0.213 | 0.597 | 0.221 | -0.269 | 0.914 |
| Eigenvector centrality | Right.superiorparietal_part5 | 0.727 | 0.158 | 0.713 | 0.168 | -0.88 | 0.696 |
| Eigenvector centrality | Right.superiorparietal_part6 | 0.44 | 0.174 | 0.425 | 0.159 | -0.868 | 0.697 |
| Eigenvector centrality | Right.superiorparietal_part7 | 0.532 | 0.172 | 0.516 | 0.182 | -1.062 | 0.651 |
| Eigenvector centrality | Right.superiorparietal_part8 | 0.549 | 0.168 | 0.526 | 0.158 | -1.675 | 0.457 |
| Eigenvector centrality | Right.superiorparietal_part9 | 0.357 | 0.141 | 0.355 | 0.145 | -0.402 | 0.872 |
| Eigenvector centrality | Right.superiorparietal_part10 | 0.428 | 0.158 | 0.419 | 0.152 | -0.71 | 0.757 |
| Eigenvector centrality | Right.superiortemporal_part1 | 0.555 | 0.156 | 0.551 | 0.161 | -0.363 | 0.88 |
| Eigenvector centrality | Right.superiortemporal_part2 | 0.509 | 0.163 | 0.5 | 0.173 | -0.816 | 0.712 |
| Eigenvector centrality | Right.superiortemporal_part3 | 0.446 | 0.17 | 0.459 | 0.188 | 0.597 | 0.8 |
| Eigenvector centrality | Right.superiortemporal_part4 | 0.507 | 0.165 | 0.525 | 0.168 | 1.039 | 0.657 |
| Eigenvector centrality | Right.superiortemporal_part5 | 0.523 | 0.181 | 0.485 | 0.187 | -2.437 | 0.233 |
| Eigenvector centrality | Right.superiortemporal_part6 | 0.702 | 0.155 | 0.725 | 0.134 | 1.713 | 0.442 |
| Eigenvector centrality | Right.supramarginal_part1 | 0.586 | 0.185 | 0.601 | 0.16 | 0.818 | 0.712 |
| Eigenvector centrality | Right.supramarginal_part2 | 0.676 | 0.173 | 0.706 | 0.156 | 1.817 | 0.423 |
| Eigenvector centrality | Right.supramarginal_part3 | 0.738 | 0.164 | 0.742 | 0.186 | 0.184 | 0.936 |
| Eigenvector centrality | Right.supramarginal_part4 | 0.569 | 0.179 | 0.576 | 0.196 | 0.443 | 0.852 |
| Eigenvector centrality | Right.supramarginal_part5 | 0.836 | 0.097 | 0.83 | 0.103 | -0.506 | 0.83 |
| Eigenvector centrality | Right.supramarginal_part6 | 0.565 | 0.176 | 0.554 | 0.182 | -0.75 | 0.736 |
| Eigenvector centrality | Right.supramarginal_part7 | 0.43 | 0.144 | 0.462 | 0.179 | 1.983 | 0.36 |
| Eigenvector centrality | Right.frontalpole_part1 | 0.448 | 0.175 | 0.446 | 0.168 | -0.508 | 0.83 |
| Eigenvector centrality | Right.temporalpole_part1 | 0.503 | 0.174 | 0.53 | 0.185 | 1.355 | 0.55 |
| Eigenvector centrality | Right.transversetemporal_part1 | 0.413 | 0.161 | 0.421 | 0.155 | 0.278 | 0.914 |
| Eigenvector centrality | Right.insula_part1 | 0.486 | 0.195 | 0.482 | 0.181 | -0.601 | 0.8 |
| Eigenvector centrality | Right.insula_part2 | 0.706 | 0.187 | 0.72 | 0.194 | 0.747 | 0.737 |
| Eigenvector centrality | Right.insula_part3 | 0.485 | 0.167 | 0.49 | 0.165 | 0.184 | 0.936 |
| Eigenvector centrality | Right.insula_part4 | 0.581 | 0.154 | 0.613 | 0.137 | 2.154 | 0.317 |
| Nodal efficiency | Left.bankssts_part1 | 0.603 | 0.025 | 0.606 | 0.023 | 1.277 | 0.577 |
| Nodal efficiency | Left.bankssts_part2 | 0.611 | 0.021 | 0.612 | 0.021 | 1.107 | 0.634 |
| Nodal efficiency | Left.caudalanteriorcingulate_part1 | 0.569 | 0.023 | 0.573 | 0.025 | 1.918 | 0.384 |
| Nodal efficiency | Left.caudalmiddlefrontal_part1 | 0.607 | 0.02 | 0.607 | 0.018 | 0.059 | 0.981 |
| Nodal efficiency | Left.caudalmiddlefrontal_part2 | 0.608 | 0.02 | 0.603 | 0.023 | -2.16 | 0.317 |
| Nodal efficiency | Left.caudalmiddlefrontal_part3 | 0.596 | 0.02 | 0.599 | 0.017 | 1.925 | 0.382 |
| Nodal efficiency | Left.caudalmiddlefrontal_part4 | 0.606 | 0.024 | 0.606 | 0.023 | 0.057 | 0.983 |
| Nodal efficiency | Left.cuneus_part1 | 0.59 | 0.011 | 0.593 | 0.01 | 2.915 | 0.163 |
| Nodal efficiency | Left.cuneus_part2 | 0.589 | 0.01 | 0.591 | 0.01 | 1.688 | 0.454 |
| Nodal efficiency | Left.entorhinal_part1 | 0.531 | 0.021 | 0.535 | 0.017 | 2.43 | 0.233 |
| Nodal efficiency | Left.fusiform_part1 | 0.575 | 0.012 | 0.578 | 0.012 | 2.232 | 0.296 |
| Nodal efficiency | Left.fusiform_part2 | 0.593 | 0.021 | 0.597 | 0.02 | 1.965 | 0.367 |
| Nodal efficiency | Left.fusiform_part3 | 0.58 | 0.016 | 0.582 | 0.017 | 1.079 | 0.644 |
| Nodal efficiency | Left.fusiform_part4 | 0.579 | 0.016 | 0.583 | 0.015 | 2.649 | 0.188 |
| Nodal efficiency | Left.fusiform_part5 | 0.584 | 0.016 | 0.586 | 0.016 | 1.394 | 0.538 |
| Nodal efficiency | Left.inferiorparietal_part1 | 0.588 | 0.018 | 0.589 | 0.018 | 0.369 | 0.879 |
| Nodal efficiency | Left.inferiorparietal_part2 | 0.585 | 0.019 | 0.586 | 0.019 | 0.547 | 0.818 |
| Nodal efficiency | Left.inferiorparietal_part3 | 0.579 | 0.015 | 0.578 | 0.016 | -0.165 | 0.941 |
| Nodal efficiency | Left.inferiorparietal_part4 | 0.595 | 0.022 | 0.596 | 0.022 | 0.396 | 0.875 |
| Nodal efficiency | Left.inferiorparietal_part5 | 0.581 | 0.019 | 0.579 | 0.018 | -1.036 | 0.657 |
| Nodal efficiency | Left.inferiorparietal_part6 | 0.59 | 0.014 | 0.59 | 0.015 | 0.574 | 0.812 |
| Nodal efficiency | Left.inferiorparietal_part7 | 0.579 | 0.017 | 0.581 | 0.015 | 1.252 | 0.59 |
| Nodal efficiency | Left.inferiorparietal_part8 | 0.58 | 0.016 | 0.581 | 0.016 | 0.33 | 0.897 |
| Nodal efficiency | Left.inferiortemporal_part1 | 0.588 | 0.021 | 0.588 | 0.021 | 0.149 | 0.941 |
| Nodal efficiency | Left.inferiortemporal_part2 | 0.579 | 0.016 | 0.583 | 0.019 | 1.843 | 0.413 |
| Nodal efficiency | Left.inferiortemporal_part3 | 0.587 | 0.018 | 0.583 | 0.02 | -1.814 | 0.423 |
| Nodal efficiency | Left.inferiortemporal_part4 | 0.575 | 0.018 | 0.576 | 0.016 | 0.623 | 0.795 |
| Nodal efficiency | Left.inferiortemporal_part5 | 0.585 | 0.02 | 0.583 | 0.018 | -1.309 | 0.567 |
| Nodal efficiency | Left.inferiortemporal_part6 | 0.574 | 0.017 | 0.576 | 0.017 | 0.906 | 0.695 |
| Nodal efficiency | Left.isthmuscingulate_part1 | 0.575 | 0.017 | 0.576 | 0.017 | 0.484 | 0.841 |
| Nodal efficiency | Left.isthmuscingulate_part2 | 0.584 | 0.025 | 0.587 | 0.028 | 1.235 | 0.591 |
| Nodal efficiency | Left.lateraloccipital_part1 | 0.592 | 0.02 | 0.595 | 0.018 | 1.255 | 0.589 |
| Nodal efficiency | Left.lateraloccipital_part2 | 0.577 | 0.027 | 0.575 | 0.027 | -0.973 | 0.673 |
| Nodal efficiency | Left.lateraloccipital_part3 | 0.601 | 0.023 | 0.604 | 0.025 | 0.941 | 0.677 |
| Nodal efficiency | Left.lateraloccipital_part4 | 0.576 | 0.018 | 0.581 | 0.019 | 2.532 | 0.212 |
| Nodal efficiency | Left.lateraloccipital_part5 | 0.56 | 0.019 | 0.563 | 0.018 | 2.172 | 0.314 |
| Nodal efficiency | Left.lateraloccipital_part6 | 0.586 | 0.013 | 0.585 | 0.015 | -0.802 | 0.719 |
| Nodal efficiency | Left.lateraloccipital_part7 | 0.583 | 0.015 | 0.582 | 0.016 | -0.048 | 0.984 |
| Nodal efficiency | Left.lateraloccipital_part8 | 0.586 | 0.011 | 0.588 | 0.009 | 2.049 | 0.348 |
| Nodal efficiency | Left.lateraloccipital_part9 | 0.58 | 0.014 | 0.581 | 0.015 | 0.845 | 0.705 |
| Nodal efficiency | Left.lateralorbitofrontal_part1 | 0.582 | 0.01 | 0.584 | 0.011 | 2.044 | 0.348 |
| Nodal efficiency | Left.lateralorbitofrontal_part2 | 0.577 | 0.016 | 0.579 | 0.014 | 1.353 | 0.55 |
| Nodal efficiency | Left.lateralorbitofrontal_part3 | 0.587 | 0.012 | 0.588 | 0.011 | 1.016 | 0.66 |
| Nodal efficiency | Left.lateralorbitofrontal_part4 | 0.581 | 0.012 | 0.582 | 0.011 | 1.744 | 0.429 |
| Nodal efficiency | Left.lingual_part1 | 0.58 | 0.014 | 0.58 | 0.013 | 0.646 | 0.786 |
| Nodal efficiency | Left.lingual_part2 | 0.579 | 0.014 | 0.582 | 0.013 | 1.835 | 0.418 |
| Nodal efficiency | Left.lingual_part3 | 0.573 | 0.015 | 0.577 | 0.015 | 2.876 | 0.168 |
| Nodal efficiency | Left.lingual_part4 | 0.581 | 0.015 | 0.581 | 0.016 | 0.007 | 0.999 |
| Nodal efficiency | Left.lingual_part5 | 0.583 | 0.02 | 0.585 | 0.021 | 0.845 | 0.705 |
| Nodal efficiency | Left.lingual_part6 | 0.584 | 0.016 | 0.585 | 0.016 | 0.921 | 0.686 |
| Nodal efficiency | Left.medialorbitofrontal_part1 | 0.583 | 0.011 | 0.585 | 0.01 | 1.637 | 0.46 |
| Nodal efficiency | Left.medialorbitofrontal_part2 | 0.582 | 0.018 | 0.58 | 0.019 | -0.84 | 0.705 |
| Nodal efficiency | Left.medialorbitofrontal_part3 | 0.586 | 0.01 | 0.588 | 0.01 | 1.665 | 0.457 |
| Nodal efficiency | Left.middletemporal_part1 | 0.58 | 0.015 | 0.582 | 0.013 | 1.755 | 0.429 |
| Nodal efficiency | Left.middletemporal_part2 | 0.584 | 0.012 | 0.585 | 0.011 | 0.649 | 0.786 |
| Nodal efficiency | Left.middletemporal_part3 | 0.554 | 0.025 | 0.561 | 0.025 | 2.705 | 0.188 |
| Nodal efficiency | Left.middletemporal_part4 | 0.579 | 0.017 | 0.58 | 0.018 | 0.947 | 0.674 |
| Nodal efficiency | Left.middletemporal_part5 | 0.566 | 0.021 | 0.571 | 0.02 | 2.742 | 0.188 |
| Nodal efficiency | Left.parahippocampal_part1 | 0.59 | 0.014 | 0.591 | 0.016 | 0.63 | 0.789 |
| Nodal efficiency | Left.parahippocampal_part2 | 0.585 | 0.014 | 0.586 | 0.013 | 1.13 | 0.63 |
| Nodal efficiency | Left.paracentral_part1 | 0.592 | 0.02 | 0.595 | 0.019 | 1.524 | 0.503 |
| Nodal efficiency | Left.paracentral_part2 | 0.588 | 0.012 | 0.59 | 0.014 | 0.973 | 0.673 |
| Nodal efficiency | Left.paracentral_part3 | 0.596 | 0.018 | 0.595 | 0.019 | -0.508 | 0.83 |
| Nodal efficiency | Left.parsopercularis_part1 | 0.611 | 0.016 | 0.608 | 0.018 | -2.04 | 0.348 |
| Nodal efficiency | Left.parsopercularis_part2 | 0.615 | 0.015 | 0.61 | 0.02 | -2.668 | 0.188 |
| Nodal efficiency | Left.parsopercularis_part3 | 0.609 | 0.021 | 0.601 | 0.025 | -3.499 | 0.053 |
| Nodal efficiency | Left.parsorbitalis_part1 | 0.604 | 0.017 | 0.604 | 0.024 | 0.405 | 0.872 |
| Nodal efficiency | Left.parstriangularis_part1 | 0.604 | 0.016 | 0.606 | 0.017 | 1.257 | 0.589 |
| Nodal efficiency | Left.parstriangularis_part2 | 0.597 | 0.022 | 0.597 | 0.022 | -0.353 | 0.885 |
| Nodal efficiency | Left.pericalcarine_part1 | 0.608 | 0.019 | 0.606 | 0.02 | -0.729 | 0.744 |
| Nodal efficiency | Left.pericalcarine_part2 | 0.619 | 0.018 | 0.617 | 0.017 | -1.039 | 0.657 |
| Nodal efficiency | Left.postcentral_part1 | 0.585 | 0.011 | 0.584 | 0.012 | -0.976 | 0.673 |
| Nodal efficiency | Left.postcentral_part2 | 0.59 | 0.015 | 0.592 | 0.015 | 1.382 | 0.541 |
| Nodal efficiency | Left.postcentral_part3 | 0.592 | 0.018 | 0.594 | 0.019 | 1.341 | 0.559 |
| Nodal efficiency | Left.postcentral_part4 | 0.585 | 0.012 | 0.586 | 0.01 | 1.519 | 0.505 |
| Nodal efficiency | Left.postcentral_part5 | 0.584 | 0.011 | 0.587 | 0.01 | 2.659 | 0.188 |
| Nodal efficiency | Left.postcentral_part6 | 0.59 | 0.014 | 0.586 | 0.015 | -3.225 | 0.084 |
| Nodal efficiency | Left.postcentral_part7 | 0.584 | 0.017 | 0.589 | 0.018 | 2.711 | 0.188 |
| Nodal efficiency | Left.postcentral_part8 | 0.598 | 0.017 | 0.597 | 0.015 | -0.878 | 0.696 |
| Nodal efficiency | Left.posteriorcingulate_part1 | 0.577 | 0.019 | 0.58 | 0.02 | 1.532 | 0.501 |
| Nodal efficiency | Left.posteriorcingulate_part2 | 0.583 | 0.021 | 0.581 | 0.022 | -1.254 | 0.589 |
| Nodal efficiency | Left.precentral_part1 | 0.585 | 0.018 | 0.588 | 0.016 | 1.219 | 0.591 |
| Nodal efficiency | Left.precentral_part2 | 0.599 | 0.019 | 0.601 | 0.019 | 1.224 | 0.591 |
| Nodal efficiency | Left.precentral_part3 | 0.583 | 0.017 | 0.585 | 0.019 | 1.234 | 0.591 |
| Nodal efficiency | Left.precentral_part4 | 0.589 | 0.02 | 0.591 | 0.02 | 1.022 | 0.66 |
| Nodal efficiency | Left.precentral_part5 | 0.555 | 0.018 | 0.556 | 0.017 | 0.632 | 0.789 |
| Nodal efficiency | Left.precentral_part6 | 0.565 | 0.027 | 0.567 | 0.028 | 1.223 | 0.591 |
| Nodal efficiency | Left.precentral_part7 | 0.614 | 0.017 | 0.612 | 0.019 | -1.161 | 0.618 |
| Nodal efficiency | Left.precentral_part8 | 0.599 | 0.025 | 0.591 | 0.028 | -2.943 | 0.163 |
| Nodal efficiency | Left.precentral_part9 | 0.608 | 0.019 | 0.609 | 0.02 | 0.831 | 0.706 |
| Nodal efficiency | Left.precuneus_part1 | 0.611 | 0.024 | 0.604 | 0.024 | -2.465 | 0.223 |
| Nodal efficiency | Left.precuneus_part2 | 0.603 | 0.03 | 0.601 | 0.03 | -0.429 | 0.857 |
| Nodal efficiency | Left.precuneus_part3 | 0.615 | 0.022 | 0.614 | 0.022 | -0.469 | 0.847 |
| Nodal efficiency | Left.precuneus_part4 | 0.614 | 0.022 | 0.608 | 0.022 | -2.466 | 0.223 |
| Nodal efficiency | Left.precuneus_part5 | 0.6 | 0.029 | 0.602 | 0.03 | 0.993 | 0.673 |
| Nodal efficiency | Left.precuneus_part6 | 0.576 | 0.021 | 0.575 | 0.021 | -0.414 | 0.864 |
| Nodal efficiency | Left.precuneus_part7 | 0.604 | 0.019 | 0.603 | 0.02 | -0.343 | 0.891 |
| Nodal efficiency | Left.rostralanteriorcingulate_part1 | 0.593 | 0.019 | 0.598 | 0.021 | 2.59 | 0.201 |
| Nodal efficiency | Left.rostralmiddlefrontal_part1 | 0.593 | 0.015 | 0.592 | 0.019 | -0.512 | 0.829 |
| Nodal efficiency | Left.rostralmiddlefrontal_part2 | 0.59 | 0.018 | 0.593 | 0.015 | 1.358 | 0.55 |
| Nodal efficiency | Left.rostralmiddlefrontal_part3 | 0.589 | 0.018 | 0.593 | 0.015 | 2.04 | 0.348 |
| Nodal efficiency | Left.rostralmiddlefrontal_part4 | 0.59 | 0.022 | 0.593 | 0.019 | 1.494 | 0.517 |
| Nodal efficiency | Left.rostralmiddlefrontal_part5 | 0.586 | 0.016 | 0.588 | 0.016 | 1.038 | 0.657 |
| Nodal efficiency | Left.rostralmiddlefrontal_part6 | 0.585 | 0.016 | 0.589 | 0.014 | 2.54 | 0.212 |
| Nodal efficiency | Left.rostralmiddlefrontal_part7 | 0.586 | 0.017 | 0.589 | 0.015 | 1.322 | 0.567 |
| Nodal efficiency | Left.rostralmiddlefrontal_part8 | 0.583 | 0.018 | 0.583 | 0.018 | -0.383 | 0.878 |
| Nodal efficiency | Left.rostralmiddlefrontal_part9 | 0.591 | 0.021 | 0.593 | 0.021 | 0.951 | 0.674 |
| Nodal efficiency | Left.rostralmiddlefrontal_part10 | 0.58 | 0.015 | 0.581 | 0.014 | 0.93 | 0.682 |
| Nodal efficiency | Left.superiorfrontal_part1 | 0.59 | 0.02 | 0.59 | 0.021 | -0.159 | 0.941 |
| Nodal efficiency | Left.superiorfrontal_part2 | 0.587 | 0.014 | 0.589 | 0.013 | 1.772 | 0.424 |
| Nodal efficiency | Left.superiorfrontal_part3 | 0.59 | 0.021 | 0.592 | 0.023 | 1.032 | 0.657 |
| Nodal efficiency | Left.superiorfrontal_part4 | 0.585 | 0.014 | 0.588 | 0.014 | 2.356 | 0.259 |
| Nodal efficiency | Left.superiorfrontal_part5 | 0.583 | 0.016 | 0.588 | 0.015 | 2.923 | 0.163 |
| Nodal efficiency | Left.superiorfrontal_part6 | 0.584 | 0.017 | 0.585 | 0.014 | 0.744 | 0.737 |
| Nodal efficiency | Left.superiorfrontal_part7 | 0.609 | 0.022 | 0.61 | 0.02 | 0.692 | 0.762 |
| Nodal efficiency | Left.superiorfrontal_part8 | 0.61 | 0.022 | 0.611 | 0.019 | 0.637 | 0.786 |
| Nodal efficiency | Left.superiorfrontal_part9 | 0.609 | 0.022 | 0.607 | 0.022 | -0.886 | 0.696 |
| Nodal efficiency | Left.superiorfrontal_part10 | 0.607 | 0.018 | 0.606 | 0.021 | -0.511 | 0.829 |
| Nodal efficiency | Left.superiorfrontal_part11 | 0.612 | 0.021 | 0.605 | 0.024 | -3.362 | 0.074 |
| Nodal efficiency | Left.superiorfrontal_part12 | 0.584 | 0.024 | 0.589 | 0.021 | 2.036 | 0.348 |
| Nodal efficiency | Left.superiorfrontal_part13 | 0.615 | 0.023 | 0.614 | 0.021 | -0.559 | 0.817 |
| Nodal efficiency | Left.superiorparietal_part1 | 0.583 | 0.025 | 0.585 | 0.026 | 0.879 | 0.696 |
| Nodal efficiency | Left.superiorparietal_part2 | 0.613 | 0.018 | 0.61 | 0.023 | -1.37 | 0.546 |
| Nodal efficiency | Left.superiorparietal_part3 | 0.608 | 0.019 | 0.606 | 0.02 | -1.204 | 0.596 |
| Nodal efficiency | Left.superiorparietal_part4 | 0.605 | 0.023 | 0.603 | 0.023 | -0.458 | 0.848 |
| Nodal efficiency | Left.superiorparietal_part5 | 0.603 | 0.026 | 0.603 | 0.022 | 0.167 | 0.941 |
| Nodal efficiency | Left.superiorparietal_part6 | 0.596 | 0.017 | 0.593 | 0.018 | -1.77 | 0.424 |
| Nodal efficiency | Left.superiorparietal_part7 | 0.582 | 0.021 | 0.582 | 0.019 | -0.132 | 0.948 |
| Nodal efficiency | Left.superiorparietal_part8 | 0.584 | 0.016 | 0.585 | 0.016 | 0.674 | 0.774 |
| Nodal efficiency | Left.superiorparietal_part9 | 0.594 | 0.018 | 0.592 | 0.018 | -0.971 | 0.673 |
| Nodal efficiency | Left.superiorparietal_part10 | 0.601 | 0.016 | 0.602 | 0.015 | 0.588 | 0.803 |
| Nodal efficiency | Left.superiortemporal_part1 | 0.578 | 0.028 | 0.559 | 0.031 | -6.87 | <0.001 |
| Nodal efficiency | Left.superiortemporal_part2 | 0.588 | 0.019 | 0.589 | 0.017 | 0.461 | 0.848 |
| Nodal efficiency | Left.superiortemporal_part3 | 0.59 | 0.021 | 0.587 | 0.019 | -1.511 | 0.511 |
| Nodal efficiency | Left.superiortemporal_part4 | 0.604 | 0.017 | 0.605 | 0.018 | 0.037 | 0.986 |
| Nodal efficiency | Left.superiortemporal_part5 | 0.606 | 0.018 | 0.603 | 0.016 | -1.86 | 0.408 |
| Nodal efficiency | Left.superiortemporal_part6 | 0.615 | 0.018 | 0.617 | 0.018 | 0.912 | 0.691 |
| Nodal efficiency | Left.superiortemporal_part7 | 0.612 | 0.014 | 0.614 | 0.014 | 1.218 | 0.591 |
| Nodal efficiency | Left.supramarginal_part1 | 0.588 | 0.016 | 0.585 | 0.015 | -1.728 | 0.436 |
| Nodal efficiency | Left.supramarginal_part2 | 0.608 | 0.017 | 0.611 | 0.016 | 2.141 | 0.32 |
| Nodal efficiency | Left.supramarginal_part3 | 0.607 | 0.022 | 0.609 | 0.019 | 0.988 | 0.673 |
| Nodal efficiency | Left.supramarginal_part4 | 0.608 | 0.021 | 0.609 | 0.02 | 0.619 | 0.796 |
| Nodal efficiency | Left.supramarginal_part5 | 0.623 | 0.016 | 0.627 | 0.013 | 2.1 | 0.328 |
| Nodal efficiency | Left.supramarginal_part6 | 0.595 | 0.019 | 0.595 | 0.015 | -0.042 | 0.985 |
| Nodal efficiency | Left.supramarginal_part7 | 0.586 | 0.017 | 0.589 | 0.018 | 1.873 | 0.407 |
| Nodal efficiency | Left.frontalpole_part1 | 0.582 | 0.018 | 0.584 | 0.015 | 1.048 | 0.654 |
| Nodal efficiency | Left.temporalpole_part1 | 0.587 | 0.021 | 0.592 | 0.019 | 2.339 | 0.259 |
| Nodal efficiency | Left.transversetemporal_part1 | 0.585 | 0.018 | 0.586 | 0.017 | 0.279 | 0.914 |
| Nodal efficiency | Left.insula_part1 | 0.595 | 0.019 | 0.592 | 0.022 | -1.733 | 0.434 |
| Nodal efficiency | Left.insula_part2 | 0.596 | 0.027 | 0.599 | 0.024 | 1.156 | 0.619 |
| Nodal efficiency | Left.insula_part3 | 0.585 | 0.02 | 0.587 | 0.018 | 1.358 | 0.55 |
| Nodal efficiency | Left.insula_part4 | 0.6 | 0.016 | 0.602 | 0.017 | 0.924 | 0.685 |
| Nodal efficiency | Right.bankssts_part1 | 0.612 | 0.019 | 0.613 | 0.019 | 0.673 | 0.774 |
| Nodal efficiency | Right.bankssts_part2 | 0.605 | 0.024 | 0.607 | 0.024 | 0.811 | 0.714 |
| Nodal efficiency | Right.caudalanteriorcingulate_part1 | 0.57 | 0.019 | 0.575 | 0.019 | 2.735 | 0.188 |
| Nodal efficiency | Right.caudalmiddlefrontal_part1 | 0.596 | 0.02 | 0.598 | 0.019 | 1.727 | 0.436 |
| Nodal efficiency | Right.caudalmiddlefrontal_part2 | 0.607 | 0.026 | 0.604 | 0.027 | -1.314 | 0.567 |
| Nodal efficiency | Right.caudalmiddlefrontal_part3 | 0.598 | 0.025 | 0.598 | 0.025 | 0.152 | 0.941 |
| Nodal efficiency | Right.caudalmiddlefrontal_part4 | 0.612 | 0.022 | 0.61 | 0.02 | -1.52 | 0.505 |
| Nodal efficiency | Right.cuneus_part1 | 0.591 | 0.012 | 0.591 | 0.012 | -0.288 | 0.911 |
| Nodal efficiency | Right.cuneus_part2 | 0.585 | 0.01 | 0.585 | 0.009 | 0.102 | 0.962 |
| Nodal efficiency | Right.cuneus_part3 | 0.588 | 0.011 | 0.59 | 0.01 | 1.848 | 0.413 |
| Nodal efficiency | Right.entorhinal_part1 | 0.538 | 0.017 | 0.542 | 0.016 | 2.125 | 0.324 |
| Nodal efficiency | Right.fusiform_part1 | 0.575 | 0.012 | 0.577 | 0.012 | 1.384 | 0.541 |
| Nodal efficiency | Right.fusiform_part2 | 0.582 | 0.015 | 0.585 | 0.014 | 1.969 | 0.366 |
| Nodal efficiency | Right.fusiform_part3 | 0.577 | 0.018 | 0.577 | 0.018 | -0.375 | 0.878 |
| Nodal efficiency | Right.fusiform_part4 | 0.59 | 0.018 | 0.588 | 0.018 | -0.98 | 0.673 |
| Nodal efficiency | Right.fusiform_part5 | 0.586 | 0.019 | 0.587 | 0.019 | 0.1 | 0.962 |
| Nodal efficiency | Right.inferiorparietal_part1 | 0.591 | 0.024 | 0.593 | 0.023 | 1.02 | 0.66 |
| Nodal efficiency | Right.inferiorparietal_part2 | 0.596 | 0.021 | 0.595 | 0.021 | -0.218 | 0.927 |
| Nodal efficiency | Right.inferiorparietal_part3 | 0.585 | 0.019 | 0.588 | 0.018 | 1.979 | 0.36 |
| Nodal efficiency | Right.inferiorparietal_part4 | 0.575 | 0.014 | 0.577 | 0.014 | 0.888 | 0.696 |
| Nodal efficiency | Right.inferiorparietal_part5 | 0.594 | 0.02 | 0.594 | 0.024 | 0.053 | 0.983 |
| Nodal efficiency | Right.inferiorparietal_part6 | 0.578 | 0.017 | 0.577 | 0.015 | -0.893 | 0.696 |
| Nodal efficiency | Right.inferiorparietal_part7 | 0.586 | 0.017 | 0.586 | 0.016 | -0.132 | 0.948 |
| Nodal efficiency | Right.inferiorparietal_part8 | 0.595 | 0.018 | 0.597 | 0.019 | 0.597 | 0.8 |
| Nodal efficiency | Right.inferiorparietal_part9 | 0.578 | 0.017 | 0.58 | 0.016 | 1.235 | 0.591 |
| Nodal efficiency | Right.inferiorparietal_part10 | 0.581 | 0.015 | 0.583 | 0.015 | 1.651 | 0.457 |
| Nodal efficiency | Right.inferiortemporal_part1 | 0.578 | 0.019 | 0.579 | 0.019 | 0.559 | 0.817 |
| Nodal efficiency | Right.inferiortemporal_part2 | 0.584 | 0.018 | 0.584 | 0.017 | -0.449 | 0.851 |
| Nodal efficiency | Right.inferiortemporal_part3 | 0.588 | 0.014 | 0.587 | 0.015 | -1.104 | 0.634 |
| Nodal efficiency | Right.inferiortemporal_part4 | 0.58 | 0.018 | 0.581 | 0.018 | 0.469 | 0.847 |
| Nodal efficiency | Right.inferiortemporal_part5 | 0.582 | 0.016 | 0.582 | 0.016 | 0.439 | 0.854 |
| Nodal efficiency | Right.isthmuscingulate_part1 | 0.576 | 0.013 | 0.578 | 0.014 | 1.781 | 0.424 |
| Nodal efficiency | Right.isthmuscingulate_part2 | 0.602 | 0.015 | 0.605 | 0.014 | 2.258 | 0.287 |
| Nodal efficiency | Right.lateraloccipital_part1 | 0.595 | 0.024 | 0.594 | 0.025 | -0.242 | 0.92 |
| Nodal efficiency | Right.lateraloccipital_part2 | 0.591 | 0.021 | 0.593 | 0.022 | 0.968 | 0.673 |
| Nodal efficiency | Right.lateraloccipital_part3 | 0.603 | 0.015 | 0.604 | 0.017 | 0.93 | 0.682 |
| Nodal efficiency | Right.lateraloccipital_part4 | 0.582 | 0.016 | 0.585 | 0.017 | 1.355 | 0.55 |
| Nodal efficiency | Right.lateraloccipital_part5 | 0.564 | 0.018 | 0.567 | 0.019 | 1.405 | 0.536 |
| Nodal efficiency | Right.lateraloccipital_part6 | 0.58 | 0.01 | 0.581 | 0.012 | 1.11 | 0.634 |
| Nodal efficiency | Right.lateraloccipital_part7 | 0.58 | 0.016 | 0.582 | 0.016 | 1.362 | 0.55 |
| Nodal efficiency | Right.lateraloccipital_part8 | 0.582 | 0.011 | 0.584 | 0.01 | 1.809 | 0.424 |
| Nodal efficiency | Right.lateraloccipital_part9 | 0.585 | 0.014 | 0.584 | 0.013 | -0.201 | 0.932 |
| Nodal efficiency | Right.lateralorbitofrontal_part1 | 0.574 | 0.012 | 0.575 | 0.013 | 1.435 | 0.525 |
| Nodal efficiency | Right.lateralorbitofrontal_part2 | 0.583 | 0.013 | 0.584 | 0.013 | 0.375 | 0.878 |
| Nodal efficiency | Right.lateralorbitofrontal_part3 | 0.579 | 0.012 | 0.58 | 0.013 | 0.59 | 0.803 |
| Nodal efficiency | Right.lateralorbitofrontal_part4 | 0.581 | 0.012 | 0.581 | 0.012 | 0.388 | 0.876 |
| Nodal efficiency | Right.lingual_part1 | 0.575 | 0.013 | 0.576 | 0.013 | 0.783 | 0.729 |
| Nodal efficiency | Right.lingual_part2 | 0.568 | 0.019 | 0.57 | 0.02 | 0.837 | 0.705 |
| Nodal efficiency | Right.lingual_part3 | 0.573 | 0.014 | 0.577 | 0.015 | 2.657 | 0.188 |
| Nodal efficiency | Right.lingual_part4 | 0.591 | 0.023 | 0.591 | 0.025 | 0.03 | 0.987 |
| Nodal efficiency | Right.lingual_part5 | 0.574 | 0.014 | 0.575 | 0.014 | 1.012 | 0.663 |
| Nodal efficiency | Right.lingual_part6 | 0.578 | 0.017 | 0.58 | 0.018 | 0.697 | 0.761 |
| Nodal efficiency | Right.medialorbitofrontal_part1 | 0.581 | 0.011 | 0.583 | 0.011 | 2.618 | 0.191 |
| Nodal efficiency | Right.medialorbitofrontal_part2 | 0.584 | 0.018 | 0.586 | 0.017 | 1.117 | 0.631 |
| Nodal efficiency | Right.medialorbitofrontal_part3 | 0.585 | 0.011 | 0.587 | 0.01 | 1.911 | 0.388 |
| Nodal efficiency | Right.middletemporal_part1 | 0.581 | 0.013 | 0.585 | 0.012 | 2.665 | 0.188 |
| Nodal efficiency | Right.middletemporal_part2 | 0.582 | 0.012 | 0.585 | 0.011 | 3.223 | 0.084 |
| Nodal efficiency | Right.middletemporal_part3 | 0.577 | 0.018 | 0.575 | 0.02 | -1.112 | 0.634 |
| Nodal efficiency | Right.middletemporal_part4 | 0.553 | 0.022 | 0.558 | 0.023 | 2.314 | 0.26 |
| Nodal efficiency | Right.middletemporal_part5 | 0.576 | 0.015 | 0.578 | 0.015 | 1.604 | 0.469 |
| Nodal efficiency | Right.middletemporal_part6 | 0.599 | 0.018 | 0.599 | 0.016 | 0.252 | 0.919 |
| Nodal efficiency | Right.parahippocampal_part1 | 0.591 | 0.018 | 0.592 | 0.016 | 0.73 | 0.744 |
| Nodal efficiency | Right.parahippocampal_part2 | 0.596 | 0.02 | 0.596 | 0.019 | -0.191 | 0.935 |
| Nodal efficiency | Right.paracentral_part1 | 0.588 | 0.016 | 0.59 | 0.015 | 0.895 | 0.696 |
| Nodal efficiency | Right.paracentral_part2 | 0.583 | 0.013 | 0.584 | 0.013 | 0.474 | 0.847 |
| Nodal efficiency | Right.paracentral_part3 | 0.602 | 0.018 | 0.601 | 0.02 | -0.725 | 0.747 |
| Nodal efficiency | Right.parsopercularis_part1 | 0.607 | 0.018 | 0.608 | 0.018 | 0.672 | 0.774 |
| Nodal efficiency | Right.parsopercularis_part2 | 0.599 | 0.026 | 0.588 | 0.032 | -3.408 | 0.068 |
| Nodal efficiency | Right.parsopercularis_part3 | 0.61 | 0.021 | 0.604 | 0.026 | -2.323 | 0.26 |
| Nodal efficiency | Right.parsorbitalis_part1 | 0.603 | 0.016 | 0.603 | 0.017 | -0.128 | 0.949 |
| Nodal efficiency | Right.parstriangularis_part1 | 0.604 | 0.015 | 0.606 | 0.017 | 0.848 | 0.705 |
| Nodal efficiency | Right.parstriangularis_part2 | 0.603 | 0.018 | 0.606 | 0.017 | 1.655 | 0.457 |
| Nodal efficiency | Right.parstriangularis_part3 | 0.599 | 0.018 | 0.596 | 0.021 | -1.647 | 0.457 |
| Nodal efficiency | Right.pericalcarine_part1 | 0.617 | 0.016 | 0.616 | 0.017 | -0.473 | 0.847 |
| Nodal efficiency | Right.pericalcarine_part2 | 0.588 | 0.011 | 0.59 | 0.009 | 1.197 | 0.601 |
| Nodal efficiency | Right.pericalcarine_part3 | 0.58 | 0.017 | 0.584 | 0.015 | 2.622 | 0.191 |
| Nodal efficiency | Right.postcentral_part1 | 0.607 | 0.019 | 0.61 | 0.018 | 1.605 | 0.469 |
| Nodal efficiency | Right.postcentral_part2 | 0.585 | 0.02 | 0.587 | 0.017 | 1.185 | 0.603 |
| Nodal efficiency | Right.postcentral_part3 | 0.583 | 0.012 | 0.584 | 0.011 | 1.297 | 0.57 |
| Nodal efficiency | Right.postcentral_part4 | 0.585 | 0.012 | 0.585 | 0.011 | 0.191 | 0.935 |
| Nodal efficiency | Right.postcentral_part5 | 0.583 | 0.013 | 0.585 | 0.012 | 1.709 | 0.444 |
| Nodal efficiency | Right.postcentral_part6 | 0.589 | 0.013 | 0.586 | 0.016 | -2.105 | 0.328 |
| Nodal efficiency | Right.postcentral_part7 | 0.593 | 0.02 | 0.596 | 0.019 | 1.68 | 0.456 |
| Nodal efficiency | Right.postcentral_part8 | 0.602 | 0.016 | 0.599 | 0.018 | -1.537 | 0.499 |
| Nodal efficiency | Right.posteriorcingulate_part1 | 0.574 | 0.019 | 0.577 | 0.019 | 1.611 | 0.469 |
| Nodal efficiency | Right.posteriorcingulate_part2 | 0.604 | 0.023 | 0.6 | 0.022 | -1.746 | 0.429 |
| Nodal efficiency | Right.precentral_part1 | 0.586 | 0.021 | 0.584 | 0.02 | -0.73 | 0.744 |
| Nodal efficiency | Right.precentral_part2 | 0.593 | 0.02 | 0.59 | 0.021 | -1.206 | 0.596 |
| Nodal efficiency | Right.precentral_part3 | 0.594 | 0.018 | 0.593 | 0.02 | -0.106 | 0.96 |
| Nodal efficiency | Right.precentral_part4 | 0.572 | 0.02 | 0.574 | 0.021 | 1.124 | 0.63 |
| Nodal efficiency | Right.precentral_part5 | 0.543 | 0.02 | 0.549 | 0.019 | 3.169 | 0.096 |
| Nodal efficiency | Right.precentral_part6 | 0.614 | 0.017 | 0.614 | 0.019 | -0.06 | 0.981 |
| Nodal efficiency | Right.precentral_part7 | 0.603 | 0.026 | 0.596 | 0.029 | -2.359 | 0.259 |
| Nodal efficiency | Right.precentral_part8 | 0.606 | 0.021 | 0.609 | 0.019 | 1.433 | 0.525 |
| Nodal efficiency | Right.precentral_part9 | 0.611 | 0.023 | 0.606 | 0.023 | -1.961 | 0.367 |
| Nodal efficiency | Right.precuneus_part1 | 0.613 | 0.023 | 0.614 | 0.02 | 0.599 | 0.8 |
| Nodal efficiency | Right.precuneus_part2 | 0.565 | 0.034 | 0.571 | 0.03 | 1.782 | 0.424 |
| Nodal efficiency | Right.precuneus_part3 | 0.61 | 0.027 | 0.61 | 0.024 | -0.295 | 0.907 |
| Nodal efficiency | Right.precuneus_part4 | 0.587 | 0.023 | 0.589 | 0.023 | 0.818 | 0.712 |
| Nodal efficiency | Right.precuneus_part5 | 0.612 | 0.022 | 0.607 | 0.029 | -2.112 | 0.328 |
| Nodal efficiency | Right.precuneus_part6 | 0.573 | 0.021 | 0.574 | 0.021 | 0.208 | 0.93 |
| Nodal efficiency | Right.precuneus_part7 | 0.592 | 0.021 | 0.594 | 0.019 | 1.048 | 0.654 |
| Nodal efficiency | Right.rostralanteriorcingulate_part1 | 0.604 | 0.018 | 0.604 | 0.018 | -0.181 | 0.937 |
| Nodal efficiency | Right.rostralmiddlefrontal_part1 | 0.586 | 0.019 | 0.586 | 0.018 | 0.142 | 0.943 |
| Nodal efficiency | Right.rostralmiddlefrontal_part2 | 0.587 | 0.017 | 0.59 | 0.015 | 1.564 | 0.483 |
| Nodal efficiency | Right.rostralmiddlefrontal_part3 | 0.59 | 0.018 | 0.591 | 0.019 | 0.383 | 0.878 |
| Nodal efficiency | Right.rostralmiddlefrontal_part4 | 0.59 | 0.014 | 0.592 | 0.016 | 1.792 | 0.424 |
| Nodal efficiency | Right.rostralmiddlefrontal_part5 | 0.577 | 0.022 | 0.583 | 0.021 | 2.693 | 0.188 |
| Nodal efficiency | Right.rostralmiddlefrontal_part6 | 0.576 | 0.015 | 0.578 | 0.013 | 1.308 | 0.567 |
| Nodal efficiency | Right.rostralmiddlefrontal_part7 | 0.582 | 0.019 | 0.586 | 0.018 | 2.169 | 0.314 |
| Nodal efficiency | Right.rostralmiddlefrontal_part8 | 0.582 | 0.014 | 0.585 | 0.013 | 1.794 | 0.424 |
| Nodal efficiency | Right.rostralmiddlefrontal_part9 | 0.588 | 0.023 | 0.591 | 0.018 | 1.42 | 0.531 |
| Nodal efficiency | Right.rostralmiddlefrontal_part10 | 0.583 | 0.016 | 0.584 | 0.015 | 0.845 | 0.705 |
| Nodal efficiency | Right.superiorfrontal_part1 | 0.589 | 0.022 | 0.588 | 0.019 | -0.445 | 0.851 |
| Nodal efficiency | Right.superiorfrontal_part2 | 0.582 | 0.016 | 0.584 | 0.015 | 0.881 | 0.696 |
| Nodal efficiency | Right.superiorfrontal_part3 | 0.587 | 0.017 | 0.589 | 0.016 | 1.256 | 0.589 |
| Nodal efficiency | Right.superiorfrontal_part4 | 0.583 | 0.021 | 0.585 | 0.017 | 1.217 | 0.591 |
| Nodal efficiency | Right.superiorfrontal_part5 | 0.581 | 0.017 | 0.583 | 0.015 | 1.125 | 0.63 |
| Nodal efficiency | Right.superiorfrontal_part6 | 0.61 | 0.02 | 0.608 | 0.02 | -0.897 | 0.696 |
| Nodal efficiency | Right.superiorfrontal_part7 | 0.606 | 0.021 | 0.608 | 0.018 | 1.187 | 0.603 |
| Nodal efficiency | Right.superiorfrontal_part8 | 0.598 | 0.021 | 0.598 | 0.023 | -0.165 | 0.941 |
| Nodal efficiency | Right.superiorfrontal_part9 | 0.6 | 0.026 | 0.602 | 0.024 | 0.782 | 0.729 |
| Nodal efficiency | Right.superiorfrontal_part10 | 0.611 | 0.02 | 0.609 | 0.021 | -0.551 | 0.818 |
| Nodal efficiency | Right.superiorfrontal_part11 | 0.576 | 0.02 | 0.577 | 0.016 | 0.689 | 0.764 |
| Nodal efficiency | Right.superiorfrontal_part12 | 0.609 | 0.02 | 0.607 | 0.021 | -1.053 | 0.653 |
| Nodal efficiency | Right.superiorfrontal_part13 | 0.582 | 0.025 | 0.585 | 0.023 | 1.664 | 0.457 |
| Nodal efficiency | Right.superiorparietal_part1 | 0.618 | 0.018 | 0.616 | 0.019 | -1.159 | 0.618 |
| Nodal efficiency | Right.superiorparietal_part2 | 0.582 | 0.022 | 0.584 | 0.027 | 1.443 | 0.525 |
| Nodal efficiency | Right.superiorparietal_part3 | 0.607 | 0.022 | 0.606 | 0.022 | -0.286 | 0.912 |
| Nodal efficiency | Right.superiorparietal_part4 | 0.595 | 0.026 | 0.596 | 0.026 | 0.381 | 0.878 |
| Nodal efficiency | Right.superiorparietal_part5 | 0.611 | 0.018 | 0.608 | 0.02 | -1.166 | 0.618 |
| Nodal efficiency | Right.superiorparietal_part6 | 0.591 | 0.019 | 0.59 | 0.016 | -0.617 | 0.796 |
| Nodal efficiency | Right.superiorparietal_part7 | 0.598 | 0.017 | 0.597 | 0.018 | -0.367 | 0.879 |
| Nodal efficiency | Right.superiorparietal_part8 | 0.6 | 0.017 | 0.596 | 0.017 | -2.242 | 0.294 |
| Nodal efficiency | Right.superiorparietal_part9 | 0.579 | 0.017 | 0.579 | 0.017 | 0.199 | 0.932 |
| Nodal efficiency | Right.superiorparietal_part10 | 0.585 | 0.02 | 0.585 | 0.018 | 0.31 | 0.904 |
| Nodal efficiency | Right.superiortemporal_part1 | 0.598 | 0.017 | 0.598 | 0.017 | -0.525 | 0.826 |
| Nodal efficiency | Right.superiortemporal_part2 | 0.597 | 0.017 | 0.596 | 0.02 | -0.899 | 0.696 |
| Nodal efficiency | Right.superiortemporal_part3 | 0.587 | 0.02 | 0.589 | 0.02 | 1.313 | 0.567 |
| Nodal efficiency | Right.superiortemporal_part4 | 0.597 | 0.016 | 0.599 | 0.015 | 0.915 | 0.69 |
| Nodal efficiency | Right.superiortemporal_part5 | 0.594 | 0.021 | 0.591 | 0.02 | -1.618 | 0.469 |
| Nodal efficiency | Right.superiortemporal_part6 | 0.607 | 0.017 | 0.61 | 0.014 | 1.645 | 0.457 |
| Nodal efficiency | Right.supramarginal_part1 | 0.601 | 0.018 | 0.601 | 0.017 | -0.032 | 0.987 |
| Nodal efficiency | Right.supramarginal_part2 | 0.609 | 0.016 | 0.611 | 0.016 | 1.076 | 0.644 |
| Nodal efficiency | Right.supramarginal_part3 | 0.616 | 0.016 | 0.616 | 0.019 | -0.087 | 0.966 |
| Nodal efficiency | Right.supramarginal_part4 | 0.601 | 0.018 | 0.602 | 0.019 | 0.547 | 0.818 |
| Nodal efficiency | Right.supramarginal_part5 | 0.626 | 0.012 | 0.624 | 0.012 | -1.12 | 0.63 |
| Nodal efficiency | Right.supramarginal_part6 | 0.602 | 0.017 | 0.601 | 0.017 | -1.028 | 0.657 |
| Nodal efficiency | Right.supramarginal_part7 | 0.583 | 0.016 | 0.586 | 0.019 | 1.229 | 0.591 |
| Nodal efficiency | Right.frontalpole_part1 | 0.59 | 0.019 | 0.59 | 0.018 | -0.15 | 0.941 |
| Nodal efficiency | Right.temporalpole_part1 | 0.593 | 0.018 | 0.595 | 0.02 | 0.831 | 0.706 |
| Nodal efficiency | Right.transversetemporal_part1 | 0.584 | 0.018 | 0.585 | 0.019 | 0.226 | 0.922 |
| Nodal efficiency | Right.insula_part1 | 0.587 | 0.023 | 0.586 | 0.021 | -0.619 | 0.796 |
| Nodal efficiency | Right.insula_part2 | 0.612 | 0.021 | 0.613 | 0.021 | 0.818 | 0.712 |
| Nodal efficiency | Right.insula_part3 | 0.578 | 0.022 | 0.58 | 0.022 | 0.839 | 0.705 |
| Nodal efficiency | Right.insula_part4 | 0.603 | 0.016 | 0.606 | 0.014 | 2.272 | 0.28 |

FDR: false discovery rate, SD: standard deviation

# **Supplementary Table 5. Associations of the mean morphometric similarity of cortical regions with cognitive functions in healthy controls**

|  | FSIQ | | CTT 1 | | Word fluency | |
| --- | --- | --- | --- | --- | --- | --- |
| Structure | rho | FDR adjusted p | rho | FDR adjusted p | rho | FDR adjusted p |
| Left.caudalmiddlefrontal_part2 | -0.051 | 0.868 | 0.202 | 0.531 | -0.014 | 0.962 |
| Left.paracentral_part3 | -0.125 | 0.701 | -0.06 | 0.868 | -0.058 | 0.868 |
| Left.postcentral_part1 | -0.124 | 0.701 | -0.05 | 0.868 | -0.026 | 0.939 |
| Left.postcentral_part5 | -0.084 | 0.868 | 0.006 | 0.962 | -0.08 | 0.868 |
| Left.precentral_part5 | 0.036 | 0.907 | 0.15 | 0.669 | 0.128 | 0.726 |
| Left.precentral_part8 | -0.181 | 0.531 | 0.18 | 0.531 | -0.076 | 0.868 |
| Left.superiorfrontal_part2 | -0.216 | 0.531 | 0.16 | 0.627 | 0.076 | 0.868 |
| Left.superiorfrontal_part6 | -0.037 | 0.907 | 0.248 | 0.531 | -0.018 | 0.962 |
| Left.superiorparietal_part5 | -0.101 | 0.781 | 0.175 | 0.531 | 0.074 | 0.868 |
| Left.superiorparietal_part7 | -0.194 | 0.531 | 0.137 | 0.701 | -0.128 | 0.726 |
| Right.fusiform_part3 | -0.055 | 0.868 | 0.005 | 0.962 | -0.215 | 0.531 |
| Right.paracentral_part2 | 0.037 | 0.907 | 0.029 | 0.917 | 0.098 | 0.868 |
| Right.precentral_part2 | -0.141 | 0.669 | 0.007 | 0.962 | -0.091 | 0.868 |
| Right.superiorparietal_part9 | -0.109 | 0.726 | 0.062 | 0.868 | -0.037 | 0.913 |

FDR: false discovery rate, FSIQ: full-scale intelligence quotient, CTT 1: color trials test 1

# **Supplementary Table 6. Associations of the mean morphometric similarity of cortical regions with clinical symptoms, illness duration, antipsychotic dose, and cognitive function in patients with schizophrenia**

|  | PANSS positive | | PANSS negative | | Illness duration | | Antipsychotic dose | | FSIQ | | CTT 1 | | Word fluency | |
| --- | --- | --- | --- | --- | --- | --- | --- | --- | --- | --- | --- | --- | --- | --- |
|  | rho | p^a^ | rho | p^a^ | rho | p^a^ | rho | p^a^ | rho | p^a^ | rho | p^a^ | rho | p^a^ |
| Left.caudalmiddlefrontal_part2 | -0.09 | 0.867 | -0.038 | 0.945 | 0.183 | 0.145 | 0.091 | 0.689 | 0.011 | 0.941 | 0.106 | 0.772 | -0.046 | 0.929 |
| Left.paracentral_part3 | -0.147 | 0.602 | -0.044 | 0.945 | -0.2 | 0.145 | -0.089 | 0.689 | 0.041 | 0.898 | 0.181 | 0.772 | 0.118 | 0.772 |
| Left.postcentral_part1 | 0.074 | 0.867 | 0.024 | 0.984 | -0.044 | 0.801 | 0.033 | 0.809 | -0.069 | 0.772 | 0.011 | 0.941 | -0.187 | 0.772 |
| Left.postcentral_part5 | 0.043 | 0.945 | -0.002 | 0.984 | -0.038 | 0.801 | 0.112 | 0.689 | 0.019 | 0.941 | 0.082 | 0.772 | 0.13 | 0.772 |
| Left.precentral_part5 | 0.004 | 0.984 | 0.075 | 0.867 | 0.054 | 0.801 | -0.05 | 0.776 | -0.099 | 0.772 | 0.116 | 0.772 | 0.099 | 0.772 |
| Left.precentral_part8 | 0.008 | 0.984 | 0.009 | 0.984 | -0.112 | 0.456 | 0.003 | 0.967 | 0.12 | 0.772 | 0.151 | 0.772 | 0.3 | 0.391 |
| Left.superiorfrontal_part2 | -0.04 | 0.945 | -0.052 | 0.945 | 0.106 | 0.456 | -0.102 | 0.689 | 0.128 | 0.772 | 0.082 | 0.772 | 0.294 | 0.391 |
| Left.superiorfrontal_part6 | -0.074 | 0.867 | -0.097 | 0.867 | -0.031 | 0.801 | -0.089 | 0.689 | 0.045 | 0.898 | 0.072 | 0.772 | 0.163 | 0.772 |
| Left.superiorparietal_part5 | 0.012 | 0.984 | -0.081 | 0.867 | -0.277 | 0.040 | 0.08 | 0.689 | 0.047 | 0.898 | 0.28 | 0.276 | -0.189 | 0.772 |
| Left.superiorparietal_part7 | -0.022 | 0.984 | 0.008 | 0.984 | 0.009 | 0.922 | -0.079 | 0.689 | -0.068 | 0.772 | -0.023 | 0.941 | 0.001 | 0.993 |
| Right.fusiform_part3 | -0.043 | 0.945 | -0.009 | 0.984 | -0.04 | 0.801 | -0.006 | 0.967 | 0.09 | 0.772 | 0.022 | 0.941 | -0.056 | 0.898 |
| Right.paracentral_part2 | -0.05 | 0.945 | -0.017 | 0.984 | -0.196 | 0.145 | -0.054 | 0.776 | 0.012 | 0.941 | 0.137 | 0.772 | 0.025 | 0.941 |
| Right.precentral_part2 | 0.045 | 0.945 | -0.097 | 0.867 | -0.113 | 0.456 | -0.053 | 0.776 | -0.068 | 0.772 | 0.033 | 0.929 | -0.077 | 0.859 |
| Right.superiorparietal_part9 | 0.154 | 0.602 | 0.107 | 0.867 | 0.189 | 0.145 | -0.034 | 0.809 | -0.15 | 0.772 | -0.09 | 0.772 | -0.137 | 0.772 |

FSIQ: full-scale intelligence quotient, CTT 1: color trials test 1, PANSS: positive and negative syndrome scale

The ^a^P value was adjusted for multiple testing using the false discovery rate (FDR) correction.

# **Supplementary Table 7. Associations of the mean morphometric similarity of subnetworks with cognitive functions in healthy controls**

|  |  | FSIQ | | CTT 1 | | Word fluency | |
| --- | --- | --- | --- | --- | --- | --- | --- |
| Atlas | Subnetwork | rho | FDR adjusted p | rho | FDR adjusted p | rho | FDR adjusted p |
| Yeo | Somatosensory | -0.106 | 0.443 | 0.133 | 0.840 | -0.193 | 0.266 |
|  | Dorsal attention | -0.123 | 0.443 | 0.046 | 0.840 | -0.224 | 0.266 |
|  | Limbic | 0.081 | 0.443 | 0.022 | 0.840 | -0.098 | 0.520 |
| Von economo | Primary motor cortex | -0.091 | 0.443 | -0.024 | 0.840 | -0.13 | 0.469 |
|  | Association cortex 1 | -0.080 | 0.443 | 0.074 | 0.840 | 0.077 | 0.525 |

FDR: false discovery rate, FSIQ: full-scale intelligence quotient, CTT 1: color trials test 1

# **Supplementary Table 8. Associations of the mean morphometric similarity of subnetworks with clinical symptoms, illness duration, antipsychotic dose, and cognitive functions in patients with schizophrenia**

|  |  | PANSS positive | | PANSS negative | | Illness duration | | Antipsychotic dose | | FSIQ | | CTT 1 | | Word fluency | |
| --- | --- | --- | --- | --- | --- | --- | --- | --- | --- | --- | --- | --- | --- | --- | --- |
| Atlas | Subnetwork | rho | p^a^ | rho | p^a^ | rho | p^a^ | rho | p^a^ | rho | p^a^ | rho | p^a^ | rho | p^a^ |
| Yeo | Somatosensory | -0.045 | 0.669 | -0.115 | 0.300 | 0.046 | 0.629 | 0.077 | 0.595 | 0.018 | 0.859 | 0.129 | 0.641 | 0.067 | 0.905 |
|  | Dorsal attention | 0.071 | 0.669 | -0.044 | 0.543 | 0.143 | 0.299 | -0.089 | 0.595 | 0.063 | 0.859 | 0.071 | 0.788 | 0.043 | 0.905 |
|  | Limbic | 0.081 | 0.669 | -0.086 | 0.300 | 0.151 | 0.299 | -0.205 | 0.070 | -0.089 | 0.859 | -0.051 | 0.788 | 0.016 | 0.905 |
| Von economo | Primary motor cortex | -0.047 | 0.669 | -0.093 | 0.300 | -0.1 | 0.362 | -0.011 | 0.897 | 0.168 | 0.462 | 0.119 | 0.641 | 0.124 | 0.905 |
|  | Association cortex 1 | 0.001 | 0.985 | 0.088 | 0.300 | 0.127 | 0.299 | -0.043 | 0.758 | -0.027 | 0.859 | 0.006 | 0.958 | -0.02 | 0.905 |

FSIQ: full-scale intelligence quotient, CTT 1: color trials test 1, PANSS: positive and negative syndrome scale

The ^a^P value was adjusted for multiple testing using the false discovery rate (FDR) correction.

# **Supplementary Table 9. Associations of the global measures of the morphometric similarity network with cognitive functions in healthy controls**

|  | FSIQ | | CTT 1 | | | Word fluency | | |
| --- | --- | --- | --- | --- | --- | --- | --- | --- |
| Global measure | rho | FDR adjusted p | rho | FDR adjusted p | | rho | FDR adjusted p | |
| Global efficiency | 0.016 | 0.88 | -0.109 | | 0.872 | 0.042 | | 0.872 |
| Modularity | 0.134 | 0.872 | 0.147 | | 0.872 | -0.021 | | 0.88 |
| Transivity | 0.048 | 0.872 | 0.098 | | 0.872 | 0.044 | | 0.872 |
| Local efficiency | 0.047 | 0.872 | 0.065 | | 0.872 | -0.043 | | 0.872 |

FDR: false discovery rate

# **Supplementary Table 10. Associations of the global measures of the morphometric similarity network with clinical symptoms, illness duration, antipsychotic dose, and cognitive function in patients with schizophrenia**

|  | PANSS positive | | PANSS negative | | Illness duration | | Antipsychotic dose | | FSIQ | | CTT 1 | | Word fluency | |
| --- | --- | --- | --- | --- | --- | --- | --- | --- | --- | --- | --- | --- | --- | --- |
| Global measure | rho | p^a^ | rho | p^a^ | rho | p^a^ | rho | p^a^ | rho | p^a^ | rho | p^a^ | rho | p^a^ |
| Global efficiency | -0.018 | 0.9 | 0.016 | 0.9 | -0.014 | 0.882 | -0.071 | 0.717 | -0.108 | 0.684 | -0.126 | 0.684 | 0.035 | 0.866 |
| Local efficiency | 0.013 | 0.9 | -0.009 | 0.9 | -0.022 | 0.882 | 0.072 | 0.717 | 0.091 | 0.684 | 0.111 | 0.684 | -0.036 | 0.866 |
| Modularity | -0.019 | 0.9 | -0.079 | 0.9 | -0.03 | 0.882 | 0.042 | 0.717 | 0.096 | 0.684 | 0.082 | 0.684 | -0.078 | 0.756 |
| Transivity | -0.017 | 0.9 | -0.042 | 0.9 | -0.05 | 0.882 | 0.03 | 0.717 | 0.079 | 0.684 | 0.078 | 0.684 | -0.023 | 0.866 |

FSIQ: full-scale intelligence quotient, CTT 1: color trials test 1, PANSS: positive and negative syndrome scale

^a^ P value is adjusted for multiple testing using the false discovery rate (FDR) correction.

# **Supplementary Table 11. Associations of the nodal measures of the morphometric similarity network with cognitive functions in healthy controls**

| Cognitive test | Nodal measure | Structure | rho | FDR adjusted p |
| --- | --- | --- | --- | --- |
| CTT 1 | Betweenness centrality | Left.superiortemporal_part1 | 0.047 | 0.985 |
| CTT 1 | Degree | Left.parsopercularis_part3 | -0.119 | 0.985 |
| CTT 1 | Degree | Left.postcentral_part6 | -0.019 | 0.985 |
| CTT 1 | Degree | Left.superiorfrontal_part11 | 0.06 | 0.985 |
| CTT 1 | Degree | Left.superiortemporal_part1 | 0.132 | 0.985 |
| CTT 1 | Degree | Right.parsopercularis_part2 | 0.066 | 0.985 |
| CTT 1 | Eigenvector centrality | Left.parsopercularis_part3 | -0.14 | 0.985 |
| CTT 1 | Eigenvector centrality | Left.superiorfrontal_part11 | 0.023 | 0.985 |
| CTT 1 | Eigenvector centrality | Left.superiortemporal_part1 | 0.082 | 0.985 |
| CTT 1 | Eigenvector centrality | Right.parsopercularis_part2 | 0.066 | 0.985 |
| CTT 1 | Nodal efficiency | Left.superiortemporal_part1 | 0.117 | 0.985 |
| FSIQ | Betweenness centrality | Left.superiortemporal_part1 | -0.002 | 0.985 |
| FSIQ | Degree | Left.parsopercularis_part3 | 0.047 | 0.985 |
| FSIQ | Degree | Left.postcentral_part6 | -0.041 | 0.985 |
| FSIQ | Degree | Left.superiorfrontal_part11 | -0.297 | 0.061 |
| FSIQ | Degree | Left.superiortemporal_part1 | -0.047 | 0.985 |
| FSIQ | Degree | Right.parsopercularis_part2 | 0.141 | 0.985 |
| FSIQ | Eigenvector centrality | Left.parsopercularis_part3 | 0.083 | 0.985 |
| FSIQ | Eigenvector centrality | Left.superiorfrontal_part11 | -0.314 | 0.061 |
| FSIQ | Eigenvector centrality | Left.superiortemporal_part1 | -0.03 | 0.985 |
| FSIQ | Eigenvector centrality | Right.parsopercularis_part2 | 0.189 | 0.564 |
| FSIQ | Nodal efficiency | Left.superiortemporal_part1 | -0.057 | 0.985 |
| Word fluency | Betweenness centrality | Left.superiortemporal_part1 | 0.004 | 0.985 |
| Word fluency | Degree | Left.parsopercularis_part3 | -0.049 | 0.985 |
| Word fluency | Degree | Left.postcentral_part6 | -0.25 | 0.389 |
| Word fluency | Degree | Left.superiorfrontal_part11 | -0.024 | 0.985 |
| Word fluency | Degree | Left.superiortemporal_part1 | 0.014 | 0.985 |
| Word fluency | Degree | Right.parsopercularis_part2 | 0.034 | 0.985 |
| Word fluency | Eigenvector centrality | Left.parsopercularis_part3 | 0.004 | 0.985 |
| Word fluency | Eigenvector centrality | Left.superiorfrontal_part11 | -0.048 | 0.985 |
| Word fluency | Eigenvector centrality | Left.superiortemporal_part1 | 0.093 | 0.985 |
| Word fluency | Eigenvector centrality | Right.parsopercularis_part2 | 0.055 | 0.985 |
| Word fluency | Nodal efficiency | Left.superiortemporal_part1 | 0.017 | 0.985 |

FDR: false discovery rate, FSIQ: full-scale intelligence quotient, CTT 1: color trials test 1

# **Supplementary Table 12. Associations of the nodal measures of the morphometric similarity network with clinical symptoms, illness duration, antipsychotic dose, and cognitive function in patients with schizophrenia**

| Category | Subcategory | Nodal measure | Structure | rho | FDR adjusted p |
| --- | --- | --- | --- | --- | --- |
| Clinical symptom | PANSS positive | Betweenness centrality | Left.superiortemporal_part1 | 0.076 | 0.724 |
|  | PANSS positive | Degree | Left.parsopercularis_part3 | -0.137 | 0.522 |
|  | PANSS positive | Degree | Left.postcentral_part6 | -0.003 | 0.965 |
|  | PANSS positive | Degree | Left.superiorfrontal_part11 | -0.008 | 0.965 |
|  | PANSS positive | Degree | Left.superiortemporal_part1 | 0.117 | 0.522 |
|  | PANSS positive | Degree | Right.parsopercularis_part2 | -0.107 | 0.522 |
|  | PANSS positive | Eigenvector centrality | Left.parsopercularis_part3 | -0.119 | 0.522 |
|  | PANSS positive | Eigenvector centrality | Left.superiorfrontal_part11 | -0.038 | 0.821 |
|  | PANSS positive | Eigenvector centrality | Left.superiortemporal_part1 | 0.077 | 0.724 |
|  | PANSS positive | Eigenvector centrality | Right.parsopercularis_part2 | -0.114 | 0.522 |
|  | PANSS positive | Nodal efficiency | Left.superiortemporal_part1 | 0.084 | 0.724 |
|  | PANSS negative | Betweenness centrality | Left.superiortemporal_part1 | -0.054 | 0.821 |
|  | PANSS negative | Degree | Left.parsopercularis_part3 | -0.009 | 0.965 |
|  | PANSS negative | Degree | Left.postcentral_part6 | 0.107 | 0.522 |
|  | PANSS negative | Degree | Left.superiorfrontal_part11 | 0.035 | 0.821 |
|  | PANSS negative | Degree | Left.superiortemporal_part1 | -0.031 | 0.821 |
|  | PANSS negative | Degree | Right.parsopercularis_part2 | -0.071 | 0.732 |
|  | PANSS negative | Eigenvector centrality | Left.parsopercularis_part3 | 0.005 | 0.965 |
|  | PANSS negative | Eigenvector centrality | Left.superiorfrontal_part11 | 0.032 | 0.821 |
|  | PANSS negative | Eigenvector centrality | Left.superiortemporal_part1 | -0.031 | 0.821 |
|  | PANSS negative | Eigenvector centrality | Right.parsopercularis_part2 | -0.043 | 0.821 |
|  | PANSS negative | Nodal efficiency | Left.superiortemporal_part1 | -0.039 | 0.821 |
| Illness duration | Illness duration | Betweenness centrality | Left.superiortemporal_part1 | -0.123 | 0.263 |
|  | Illness duration | Degree | Left.parsopercularis_part3 | -0.154 | 0.187 |
|  | Illness duration | Degree | Left.postcentral_part6 | -0.041 | 0.668 |
|  | Illness duration | Degree | Left.superiorfrontal_part11 | 0.088 | 0.433 |
|  | Illness duration | Degree | Left.superiortemporal_part1 | -0.223 | 0.037 |
|  | Illness duration | Degree | Right.parsopercularis_part2 | -0.255 | 0.034 |
|  | Illness duration | Eigenvector centrality | Left.parsopercularis_part3 | -0.138 | 0.224 |
|  | Illness duration | Eigenvector centrality | Left.superiorfrontal_part11 | 0.079 | 0.442 |
|  | Illness duration | Eigenvector centrality | Left.superiortemporal_part1 | -0.227 | 0.037 |
|  | Illness duration | Eigenvector centrality | Right.parsopercularis_part2 | -0.239 | 0.037 |
|  | Illness duration | Nodal efficiency | Left.superiortemporal_part1 | -0.263 | 0.034 |
| Antipsychotic dose | Antipsychotic dose | Betweenness centrality | Left.superiortemporal_part1 | 0.088 | 0.568 |
|  | Antipsychotic dose | Degree | Left.parsopercularis_part3 | -0.203 | 0.164 |
|  | Antipsychotic dose | Degree | Left.postcentral_part6 | 0.017 | 0.836 |
|  | Antipsychotic dose | Degree | Left.superiorfrontal_part11 | -0.083 | 0.568 |
|  | Antipsychotic dose | Degree | Left.superiortemporal_part1 | 0.046 | 0.718 |
|  | Antipsychotic dose | Degree | Right.parsopercularis_part2 | -0.077 | 0.568 |
|  | Antipsychotic dose | Eigenvector centrality | Left.parsopercularis_part3 | -0.168 | 0.24 |
|  | Antipsychotic dose | Eigenvector centrality | Left.superiorfrontal_part11 | -0.107 | 0.568 |
|  | Antipsychotic dose | Eigenvector centrality | Left.superiortemporal_part1 | 0.038 | 0.718 |
|  | Antipsychotic dose | Eigenvector centrality | Right.parsopercularis_part2 | -0.099 | 0.568 |
|  | Antipsychotic dose | Nodal efficiency | Left.superiortemporal_part1 | 0.041 | 0.718 |
| Cognitive test | FSIQ | Betweenness centrality | Left.superiortemporal_part1 | 0.038 | 0.922 |
|  | FSIQ | Degree | Left.parsopercularis_part3 | 0.011 | 0.922 |
|  | FSIQ | Degree | Left.postcentral_part6 | -0.053 | 0.922 |
|  | FSIQ | Degree | Left.superiorfrontal_part11 | 0.098 | 0.827 |
|  | FSIQ | Degree | Left.superiortemporal_part1 | -0.029 | 0.922 |
|  | FSIQ | Degree | Right.parsopercularis_part2 | 0.122 | 0.746 |
|  | FSIQ | Eigenvector centrality | Left.parsopercularis_part3 | 0.024 | 0.922 |
|  | FSIQ | Eigenvector centrality | Left.superiorfrontal_part11 | 0.103 | 0.827 |
|  | FSIQ | Eigenvector centrality | Left.superiortemporal_part1 | 0.021 | 0.922 |
|  | FSIQ | Eigenvector centrality | Right.parsopercularis_part2 | 0.125 | 0.746 |
|  | FSIQ | Nodal efficiency | Left.superiortemporal_part1 | -0.015 | 0.922 |
|  | CTT 1 | Betweenness centrality | Left.superiortemporal_part1 | 0.051 | 0.922 |
|  | CTT 1 | Degree | Left.parsopercularis_part3 | 0.085 | 0.865 |
|  | CTT 1 | Degree | Left.postcentral_part6 | -0.01 | 0.922 |
|  | CTT 1 | Degree | Left.superiorfrontal_part11 | 0.06 | 0.922 |
|  | CTT 1 | Degree | Left.superiortemporal_part1 | 0.114 | 0.825 |
|  | CTT 1 | Degree | Right.parsopercularis_part2 | 0.179 | 0.438 |
|  | CTT 1 | Eigenvector centrality | Left.parsopercularis_part3 | 0.085 | 0.865 |
|  | CTT 1 | Eigenvector centrality | Left.superiorfrontal_part11 | 0.095 | 0.864 |
|  | CTT 1 | Eigenvector centrality | Left.superiortemporal_part1 | 0.193 | 0.421 |
|  | CTT 1 | Eigenvector centrality | Right.parsopercularis_part2 | 0.204 | 0.414 |
|  | CTT 1 | Nodal efficiency | Left.superiortemporal_part1 | 0.127 | 0.746 |
|  | Word fluency | Betweenness centrality | Left.superiortemporal_part1 | -0.091 | 0.922 |
|  | Word fluency | Degree | Left.parsopercularis_part3 | 0.041 | 0.922 |
|  | Word fluency | Degree | Left.postcentral_part6 | -0.364 | 0.097 |
|  | Word fluency | Degree | Left.superiorfrontal_part11 | 0.368 | 0.097 |
|  | Word fluency | Degree | Left.superiortemporal_part1 | 0.09 | 0.922 |
|  | Word fluency | Degree | Right.parsopercularis_part2 | 0.028 | 0.922 |
|  | Word fluency | Eigenvector centrality | Left.parsopercularis_part3 | 0.072 | 0.922 |
|  | Word fluency | Eigenvector centrality | Left.superiorfrontal_part11 | 0.319 | 0.181 |
|  | Word fluency | Eigenvector centrality | Left.superiortemporal_part1 | -0.227 | 0.438 |
|  | Word fluency | Eigenvector centrality | Right.parsopercularis_part2 | 0.032 | 0.922 |
|  | Word fluency | Nodal efficiency | Left.superiortemporal_part1 | 0.027 | 0.922 |

FSIQ: full-scale intelligence quotient, CTT 1: color trials test 1, PANSS: positive and negative syndrome scale

# **Supplementary Table 13. Associations of the nodal measures of the morphometric similarity network with illness duration adjusted for age**

| Structure | Nodal measure | rho | FDR adjusted p |
| --- | --- | --- | --- |
| Left.superiortemporal_part1 | Degree | -0.115 | 0.312 |
| Left.superiortemporal_part1 | Eigenvector centrality | -0.195 | 0.229 |
| Left.superiortemporal_part1 | Nodal efficiency | -0.184 | 0.229 |
| Right.parsopercularis_part2 | Degree | -0.156 | 0.229 |
| Right.parsopercularis_part2 | Eigenvector centrality | -0.138 | 0.229 |

**
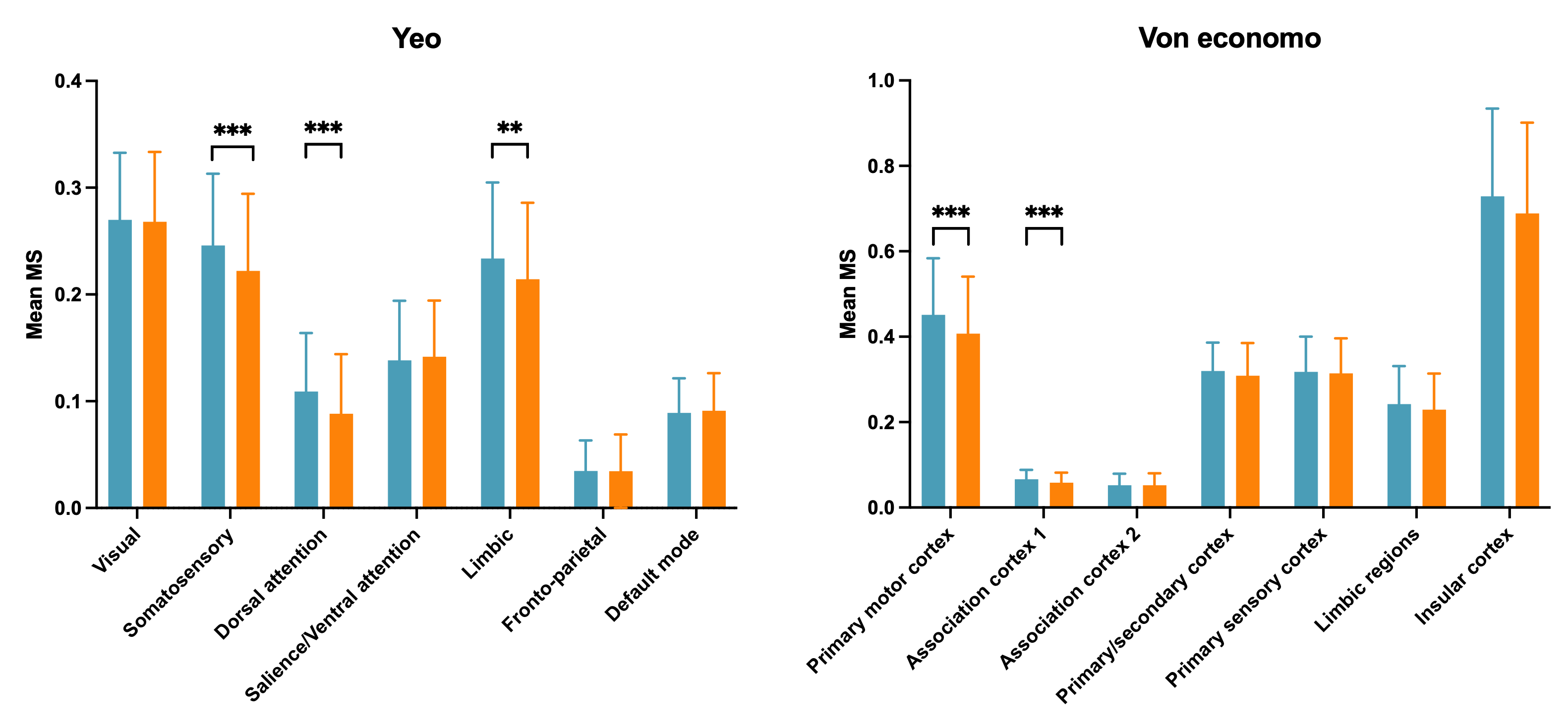
**

**Supplementary Figure 1. Group comparisons of the mean morphometric similarity of subnetworks.** The blue and orange bars indicate the healthy control and schizophrenia patient groups, respectively. MS, morphometric similarity. ** FDR p < 0.01, *** FDR p < 0.005.

# **References**

1 Rosen, A. F. G. *et al.* Quantitative assessment of structural image quality. *Neuroimage* **169**, 407-418 (2018). <https://doi.org:10.1016/j.neuroimage.2017.12.059>

2 Cetin Karayumak, S. *et al.* Retrospective harmonization of multi-site diffusion MRI data acquired with different acquisition parameters. *Neuroimage* **184**, 180-200 (2019). <https://doi.org:10.1016/j.neuroimage.2018.08.073>

3 Mirzaalian, H. *et al.* Multi-site harmonization of diffusion MRI data in a registration framework. *Brain Imaging Behav* **12**, 284-295 (2018). <https://doi.org:10.1007/s11682-016-9670-y>

4 Zhang, S. & Arfanakis, K. Evaluation of standardized and study-specific diffusion tensor imaging templates of the adult human brain: Template characteristics, spatial normalization accuracy, and detection of small inter-group FA differences. *Neuroimage* **172**, 40-50 (2018). <https://doi.org:10.1016/j.neuroimage.2018.01.046>
